# Systematics of Spiny Predatory Katydids (Tettigoniidae: Listroscelidinae) from the Brazilian Atlantic Forest Based on Morphology and Molecular Data

**DOI:** 10.1371/journal.pone.0103758

**Published:** 2014-08-13

**Authors:** Verônica Saraiva Fialho, Juliana Chamorro-Rengifo, Cristiano Lopes-Andrade, Karla Suemy Clemente Yotoko

**Affiliations:** 1 Programa de Pós-Graduação em Entomologia, Departamento de Entomologia, Universidade Federal de Viçosa, Viçosa, Minas Gerais, Brasil; 2 Laboratório de Sistemática e Biologia de Coleoptera, Departamento de Biologia Animal, Universidade Federal de Viçosa, Viçosa, Minas Gerais, Brasil; 3 Laboratório de Bioinformática e Evolução, Departamento de Biologia Geral, Universidade Federal de Viçosa, Viçosa, Minas Gerais, Brasil; Sars International Centre for Marine Molecular Biology, Norway

## Abstract

Listroscelidinae (Orthoptera: Tettigoniidae) are insectivorous Pantropical katydids whose taxonomy presents a long history of controversy, with several genera incertae sedis. This work focused on species occurring in the Brazilian Atlantic Forest, one of the world's most threatened biomes. We examined material deposited in scientific collections and visited 15 conservation units from Rio de Janeiro to southern Bahia between November 2011 and January 2012, catching 104 specimens from 10 conservation units. Based on morphological and molecular data we redefined Listroscelidini, adding a new tribe, new genus and eight new species to the subfamily. Using morphological analysis, we redescribed and added new geographic records for six species, synonymized two species and built a provisional identification key for the Atlantic Forest Listroscelidinae. Molecular results suggest two new species and a new genus to be described, possibly by the fission of the genus *Hamayulus*. We also proposed a 500 bp region in the final portion of the COI to be used as a molecular barcode. Our data suggest that the Atlantic Forest Listroscelidinae are seriously endangered, because they occur in highly preserved forest remnants, show high rates of endemism and have a narrow geographic distribution. Based on our results, we suggest future collection efforts must take into account the molecular barcode data to accelerate species recognition.

## Introduction

Listroscelidinae (Orthoptera: Tettigoniidae) are carnivorous (insectivorous) katydids [1] with a long and controversial taxonomic history. In 1891, Redtenbacher [2] placed these species in the tribe “Listroscelini”, which he included in Conocephalinae, with *Listroscelis* Serville as the type genus. This tribe included a few genera now classified in two other subfamilies, Meconematinae and Hexacentrinae. In 1898, Saussure & Pictet [3] separated *Listroscelis* into “Listroscelites”, but examined only *L. arachnoides* Redtenbacher, the type-species of *Arachnoscelis*, further described by Karny [4]. In 1906, Kirby [5] raised the tribe to the rank of subfamily and in 1915 Bruner [1] treated the group as a family and provided a taxonomic key for tropical American genera. In 1924, Karny [6] transferred a few genera to Meconematinae and in 1936 Zeuner [7], [8] reevaluated Tettigoniidae, examining traditional characteristics of the head, wing venation and prothoracic tracheal apparatus. He proposed two closely related taxa: “Conocephaloids”, including Listroscelidinae (treated as a subfamily), Salomoninae (including Agraeciinae) and Copiphorinae; and “Tettigonioids”, including Tettigoniinae, Decticinae, Saginae, Mecopodinae and Phyllophorinae. In 1840, Zeuner [9] transferred *Xiphidiopsis* Redtenbacher, *Phlugis* Stål and *Phlugiola* Karny to Meconematinae but some doubts remained as he considered the differences between Meconematinae and Listroscelidinae to be only gradual. In 1979, Rentz [10] corrected the suprageneric name to Listroscelidinae, redefining it to include species with fastigium of vertex narrow and usually sulcated, fore and mid femora with robust spines and fore tibia with five to seven long spines.

The first column of Table 1 lists the current taxonomic classification of Listroscelidinae. Of the 22 known genera, 12 are organized into four tribes, while the remaining are incertae sedis. Of these, *Arachnoscelis* (incertae sedis in Table 1) was previously considered in Meconematinae [11], [12]. Except for *Neobarrettia* Rehn that occurs in the Holarctic kingdom (southern North America) [13], the subfamily is Pantropical, with most species described in Neotropical, Oriental and Australotropical regions [14]. Listroscelidinae is highly diversified in Brazil with fourteen species in eight genera, six of which occur in the Atlantic Forest. The last taxonomic study was performed by Piza [15] in 1982. Nothing is known about the natural history or distribution of the Brazilian species.

**Table 1:** Taxonomic classification of Listroscelidinae.

Tribe	Before our work	After our work
**Conocephalomimini**	*Conocephalomima*	*Conocephalomima*
**Hamayulini trib. nov.**		*Hamayulus* **gen. nov.** (1)*
**Listroscelidini**	*Listroscelis* (4)	*Listroscelis* (11)**
		*Carliella*
		*Cerberodon* (2)***
		*Isocarliella*
		*Macrometopon*
		*Monocerophora* (3)†
**Requenini**	*Requena*	*Requena*
	*Thumelinia*	*Thumelinia*
	*Xingbaoia*	*Xingbaoia*
**Terpandrini**	*Burnuia*	*Burnuia*
	*Chlorobalius*	*Chlorobalius*
	*Megatympanon* (1)	*Megatympanon* (1)
	*Neobarrettia*	*Neobarrettia*
	*Terpandrus*	*Terpandrus*
	*Yullandria*	*Yullandria*
	*Yutjuwalia*	*Yutjuwalia*
**Incertae sedis**	*Alinjarria*	*Alinjarria*
	*Arachnoscelis*	*Arachnoscelis*
	*Carliella* (1)	*Liostethomimus*
	*Cerberodon* (3)	*Paralistroscelis*
	*Isocarliella* (1)	*Poecilomerus*
	*Liostethomimus* (1)	
	*Macrometopon* (1)	
	*Monocerophora* (2)	
	*Paralistroscelis*	
	*Poecilomerus*	

Classification of Listroscelidinae genera in tribes before and after the taxonomic review performed in this work. The numbers between parentheses are of described Brazilian species for each genus.

* Plus one possible new species, recognized in the molecular analyses but not described here.

** Six species described here plus *L. angustifrons* **comb. nov.**

*** *Cerberodon cuiabensis* was synonymized with *Carliella mandibularis*, and *Cerberodon angustifrons* was transferred to *Listroscelis*.

† Plus one possible new species, *Monocerophora* sp.; and a taxon reinstated: *M. minax* was formerly synonymized with *M. longispina* [2], but we consider them as being distinct species.

Regarding phylogenetics, few molecular studies have focused on relationships within Tettigoniidae [12], [16]–[20]. Only two works have included more than one species of Listroscelidinae [12], [18], with no studies focusing on the Brazilian species. Jost and Shaw [18] included two Australian Listroscelidinae: *Requena verticalis* Walker (Requenini) and *Yutjuwalia* sp. (Terpandrini) and suggested the subfamily is paraphyletic. Mugleston et al. [12] included three species: *Arachnoscelis rehni* Randell from Costa Rica, *Meiophisis micropennis* Jin from Pappua New Guinea and *Requena* sp. from Australia, also suggesting paraphyly for this subfamily (but Eades et al. [14] considered both *A. rehni* and *M. micropennis* as Meconematinae). The only morphology-based phylogenetic analysis of listroscelidines indicates the subfamily is polyphyletic and that *Carliella* Karny, *Cerberodon* Perty, *Listroscelis* and *Monocerophora* Walker form a clade [21].

In this work, we aimed to revisit the classification of Listroscelidinae, focusing on species occurring in the Atlantic Forest, one of the world's most diverse and threatened biomes [22]–[26]. We did an extensive field expedition, visiting 15 protected forest areas to search for specimens of Listroscelidinae, which we identified, described or redescribed. We revised the taxonomy of Listroscelidinae and proposed phylogenetic hypotheses based on molecular markers for the studied species, in addition to proposing the use of a small mitochondrial sequence for molecular barcoding to accelerate future species recognition.

## Materials and Methods

### Abbreviations and depositories

In most cases, we identified the components of the phallus with the terminology and abbreviations of Snodgrass [27], and the components of the male postabdomen with the abbreviations of Ingrisch [28].

Table 2 shows the abbreviations for measurements (in mm) and counting parts used in this work. In our descriptions, we provided measurements and ratios of holotype and allotype, and the range of variation for males and females of the whole type series or examined specimens. Some measurements were obtained from literature.

**Table 2:** Abbreviations for measures and counting parts used to describe and redescribe Listroscelidinae species.

Abbreviation	Meaning
EyeW	Minimum Eye Width
FF	Length of Fore Femur
FT	Length of Fore Tibia
HF	Length of Hind Femur
HT	Length of Hind Tibia
maxT	Maximum Tooth length
minT	Minimum Tooth length
NT	Number of Teeth in the stridulatory file
OL	Length of the Ovipositor
PL	Pronotal Length at midline
PW	Maximum Pronotal Width
sFF*	Number of spines on Fore Femur
SL	Length of the Stridulatory file of male tegmen
sHF*	Number of spines on Hind Femur
sHTd	Number of spines on Hind Tibia, dorsally
sHTv	Number of spines on Hind Tibia, ventrally
sMF*	Number of spines on Mid Femur
sMTld	Number of spines on Mid Tibia, dorsally
TegL	Maximum Tegmina Length
TL	Total body Length

* Number of spines on inner and outer margins of left and right femora and tibiae.

Specimens were deposited in or belong to the following institutional collections (each preceded by its respective acronym):

**CELC** Coleção Entomológica do Laboratório de Sistemática e Biologia de Coleoptera, Universidade Federal de Viçosa (Viçosa, Minas Gerais, Brasil)

**ESALQ** Museu de Entomologia da Escola Superior de Agricultura Luiz de Queiroz (Piracicaba, São Paulo, Brasil)

**IBB** Coleção de Insetos do Departamento de Zoologia do Instituto de Biociências de Botucatu, Universidade Estadual Paulista (Botucatu, São Paulo, Brasil)

**MNRJ** Museu Nacional do Rio de Janeiro (Rio de Janeiro, Rio de Janeiro, Brasil)

**NMW** Naturhistorisches Museum Wien (Wien, Austria)

**UFES** Coleção Entomológica da Universidade Federal do Espírito Santo (Vitória, Espírito Santo, Brasil)

### Specimens sampling, preservation and photographing

**Figure 1:**
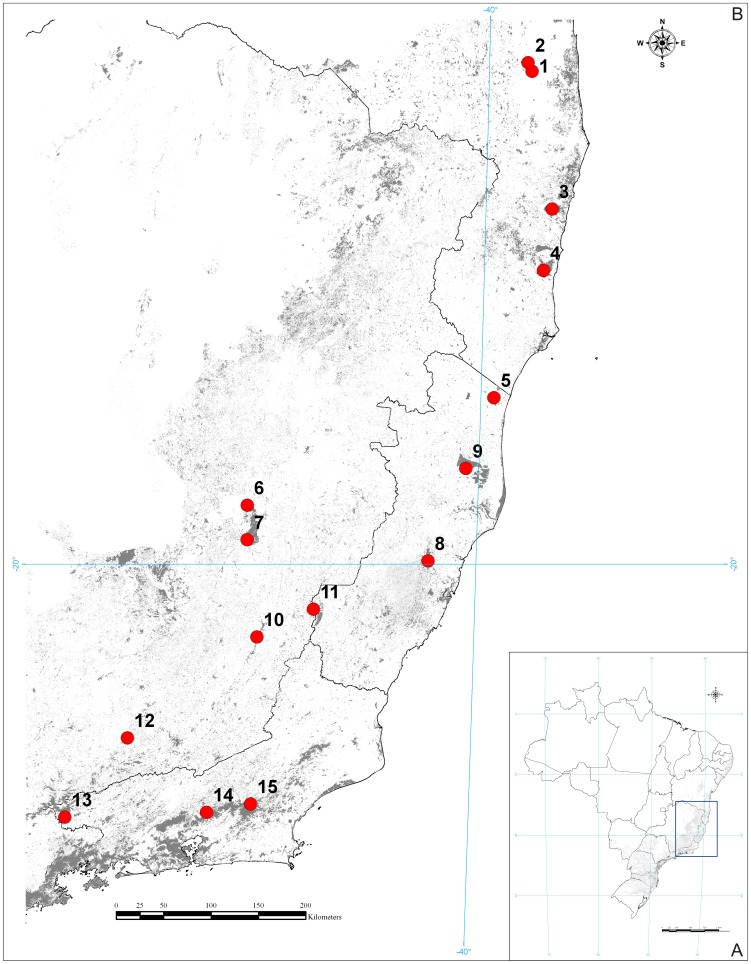
Maps showing the Atlantic Forest and sampled conservation units. Areas in light gray represent the supposed forest cover in 1500 AC (the age of Portuguese Discoveries). Areas in dark grey represent the remaining fragments of the Atlantic Forest. (A) Map of Brazil showing the AF, with the sampled area delimited by a blue rectangle (the area shown in B). (B) The 15 conservation units visited for sampling katydids (red circles). Numbers indicate the conservation units, as follows: 1 = Reserva Particular do Patrimônio Natural Serra do Teimoso, 2 = Reserva Particular do Patrimônio Natural Serra Bonita, 3 = Parque Nacional do Pau Brasil, 4 = Parque Nacional do Descobrimento, 5 = Floresta Nacional do Rio Preto, 6 = Reserva Particular do Patrimônio Natural Sítio do Zaca, 7 = Parque Estadual do Rio Doce, 8 = Estação Biológica Santa Lúcia, 9 = Reserva Biológica de Sooretama, 10 = Parque Estadual Serra do Brigadeiro, 11 = Parque Nacional do Caparaó, 12 = Parque Estadual do Ibitipoca, 13 = Parque Nacional do Itatiaia, 14 = Parque Nacional da Serra dos Órgãos, 15 = Reserva Particular do Patrimônio Natural Bacchus. Geographic coordinates of the conservation units are listed in Table S2.

From November 2011 to January 2012, we sampled specimens of Listroscelidinae in 15 conservation units of the Atlantic Forest from northern Rio de Janeiro to southern Bahia (Fig. 1A–B, Table S2). Field collections in federal conservation units were authorized by the Instituto Chico Mendes de Conservação da Biodiversidade (ICMBio) through Sistema de Autorização e Informação em Biodiversidade (SISBIO; authorization number 31135-2). Collections in conservation units administrated by the state of Minas Gerais were authorized by Instituto Estadual de Florestas (IEF; authorization numbers 093/11, 094/11, 095/11, 096/11, 097/11).

As with most katydids, Listroscelidinae are active at night; thus we collected them by active capture between 7:00 pm and 1:00 am with the aid of flashlights for at least three consecutive nights in each conservation unit. The specimens were photographed while alive and captured in transparent polyethylene vials of 500 ml with lids (pictures shown in Figs. S1, S2, S3, S4). Except for the individuals of *Hamayulus* **gen. nov.**, which were completely preserved in alcohol, we dry-preserved the specimens in field and removed their intestines to better preserve their original surface color. For molecular procedures, we preserved one mid or hind leg of each individual in absolute ethanol and kept samples below −20°C.

Photographs of preserved specimens in lateral, dorsal and frontal views were taken using a Nikon D90 digital camera equipped with a Nikon AF-S 105 mm f/2.8 VR Micro-NIKKOR lens, except for specimens provided by the staff of the NMW. Specimens immersed in alcohol were photographed under a Zeiss Discovery V8 stereomicroscope equipped with a Zeiss MRc digital camera. Images of live specimens provided in the Supporting Information (Figs. S1, S2, S3, S4) were taken with a Nikon 4500.

### Descriptions of new species and taxonomic review

For morphological analyses, we used specimens deposited in scientific collections and field-collected specimens that had been dried, pinned and labeled at the laboratory.

**Figure 2:**
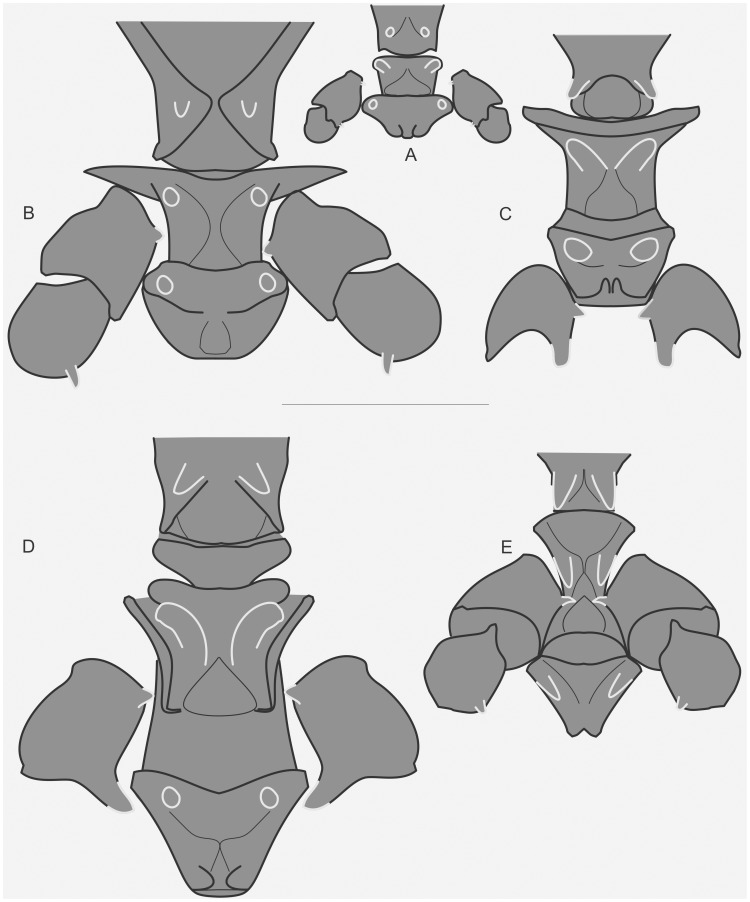
Spines and tubercles on thorax and legs. Diagrams in ventral view showing the sternum, coxae or trochanters. For each diagram, only coxa or trochanter of one leg were drawn. Spines and tubercles are indicated by light grey lines. Hamayulini **trib. nov.**: (A) *Hamayulus rufomaculatus* **sp. nov.**, spines on sternum and mid coxae. B–D Listroscelidini: (B) *Cerberodon viridis* Perty, spines on sternum, mid coxae and mid trochanters, (C) *Listroscelis carinata* Karny, spines on sternum and spine and tubercle in mid coxae, (D) *Monocerophora minax* Walker, **reinstated status**, spines on sternum and spine and tubercle in mid coxae. Terpandrini: (E) *Megatympanon speculatum* Piza, spines on sternum, mid coxae and mid trochanters. All diagrams are in the same scale. Scale bars = 0.5 mm.

**Figure 3:**
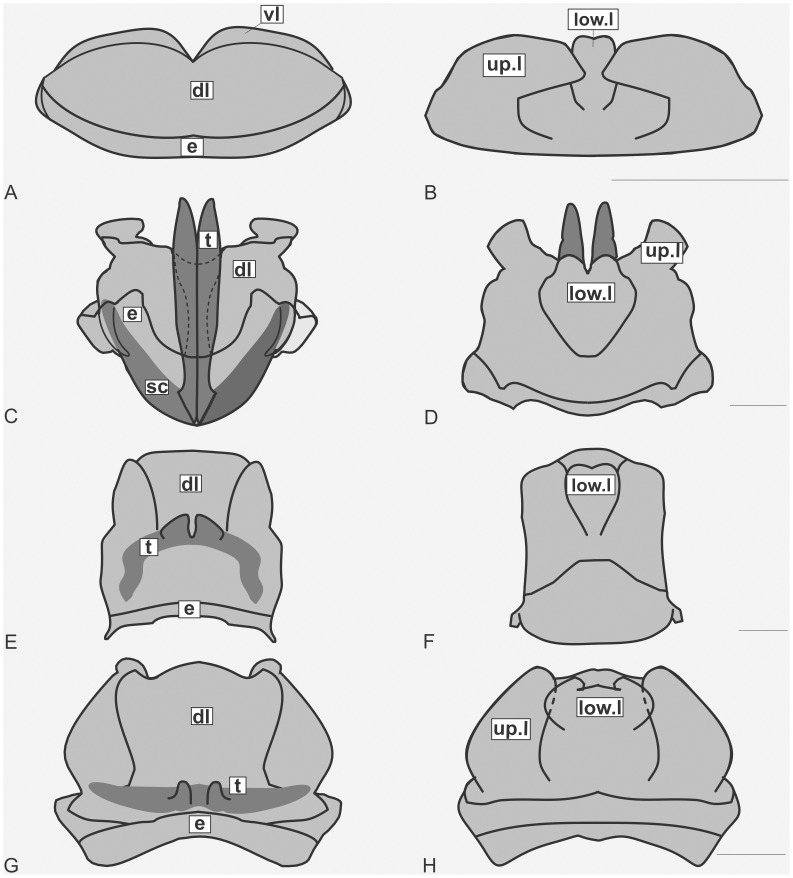
Gross morphology of the phallus. Diagrams of dorsal and ventral views of the external components of the phallus. Hamayulini **trib. nov.**: A–B *Hamayulus rufomaculatus* **sp. nov.**, (A) dorsal view, (B) ventral view. Listroscelidinae: C–D *Cerberodon viridis* Perty, (C) dorsal view, (D) ventral view. E–F *Listroscelis magnomaculata* **sp. nov**., (E) dorsal view, (F) ventral view. G–H *Monocerophora minax* Walker, **reinstated status** (G) dorsal view, (H) ventral view. Scale bars = 1.0 mm. Components of the phallus colored in light grey are membranous, and in dark grey sclerotized. Edges of membranous and sclerotized components devoid of membranes are in black line, and edges of portions or entire sclerotized components that are contiguous to membranes are in light grey. **Abbreviations** (mainly based on Snodgrass [27]): **dl**: dorsal lobe, **e**: anterior margin, **low.l**: lower lobe, **sc**: sclerite, **t**: titillator, **up.l**: upper lobe, and **vl**: ventral lobe.

**Figure 4:**
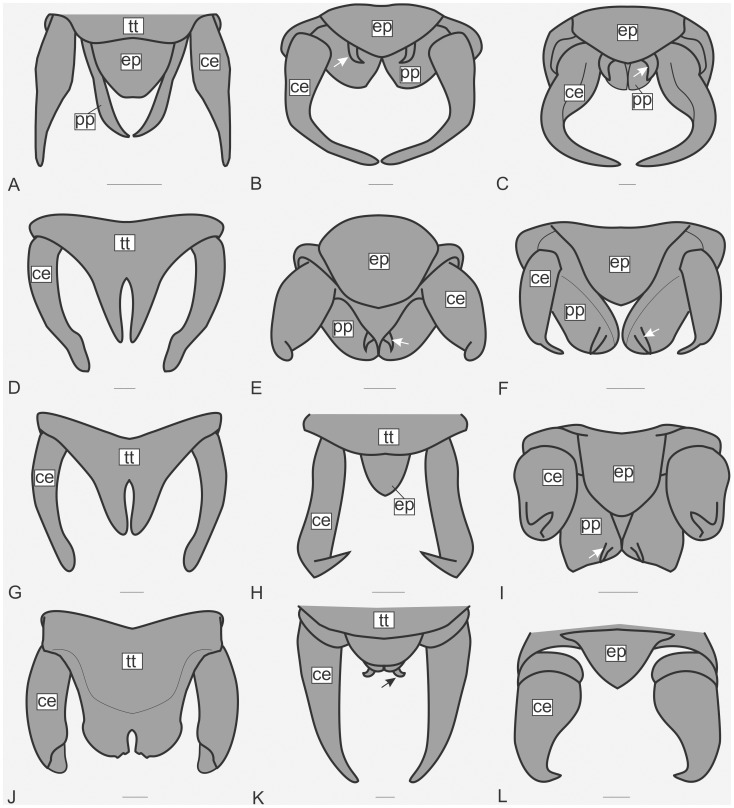
Male postabdomen. Diagrams in dorsal view showing the form of the epiproct, cerci, and sometimes the tergite X and paraprocts. Hamayulini **trib. nov.**: (A) *Hamayulus rufomaculatus* **sp. nov.**: B–K Listroscelidini: (B) *Cerberodon viridis* Perty, (C) *Cerberodon portokalipes* **sp. nov.**, (D) *Listroscelis carinata* Karny, (E) *Listroscelis angustifrons* **comb. nov.**, (F) *Listroscelis magnomaculata* **sp. nov.**, (G) *Listroscelis sooretama* **sp. nov.**, (H) *Listroscelis cohni* **sp. nov.**, (I) *Listroscelis fusca* **sp. nov.**, (J) *Listroscelis monnei* **sp. nov.**, (K) *Monocerophora minax* Walker, **reinstated status**. Terpandrini: (L) *Megatympanon speculatum* Piza. Scale bars = 0.5 mm. **Abbreviations** (manly based on Ingrisch [28]): **ce**: cercus, **ep**: epiproct, **pp**: paraproct, **tt**: tergite X. Arrows indicate the curved spine on **pp**.

**Figure 5:**
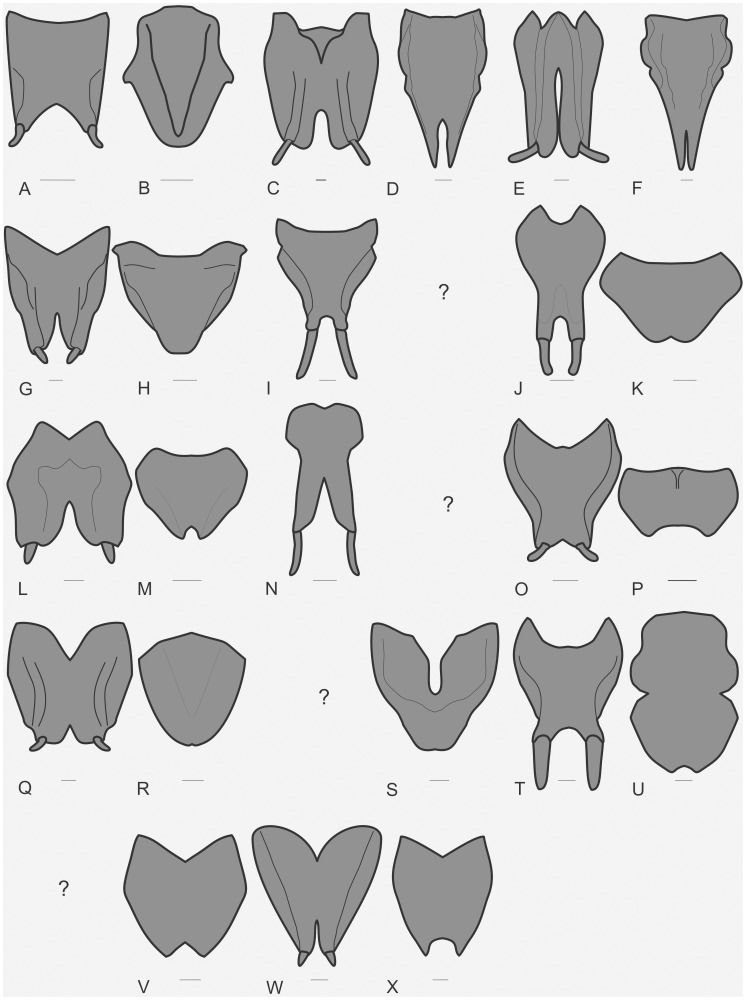
Male and female subgenital plates. Diagrams in ventral view. Hamayulini **trib. nov.**: A–B *Hamayulus rufomaculatus* **sp. nov.**, (A) male, (B) female. Listroscelidini: C–D *Cerberodon viridis* Perty, (C) male, (D) female. E–F *Cerberodon portokalipes* **sp. nov.**, (E) male, (F) female. G–H *Listroscelis carinata* Karny, (G) male, (H) female. (I) *Listroscelis angustifrons* **comb. nov.**, male. J–K *Listroscelis magnomaculata* **sp. nov.**, (J) male, (K) female. L–M *Listroscelis sooretama* **sp. nov.**, (L) male, (M) female. (N) *Listroscelis cohni* **sp. nov.**, male. O–P *Listroscelis fusca* **sp. nov.**, (O) male, (P) female. Q–R *Listroscelis monnei* **sp. nov.**, (Q) male, (R) female. (S) *Listroscelis itatiaia* **sp. nov.**, female. T–U *Monocerophora minax* Walker, **reinstated status**, (T) male, (U) female. (V) *Monocerophora spinosa* (Karny), female. Terpandrini: W–X *Megatympanon speculatum* Piza, (W) male, (X) female. Scale bars = 0.5 mm.

**Figure 6:**
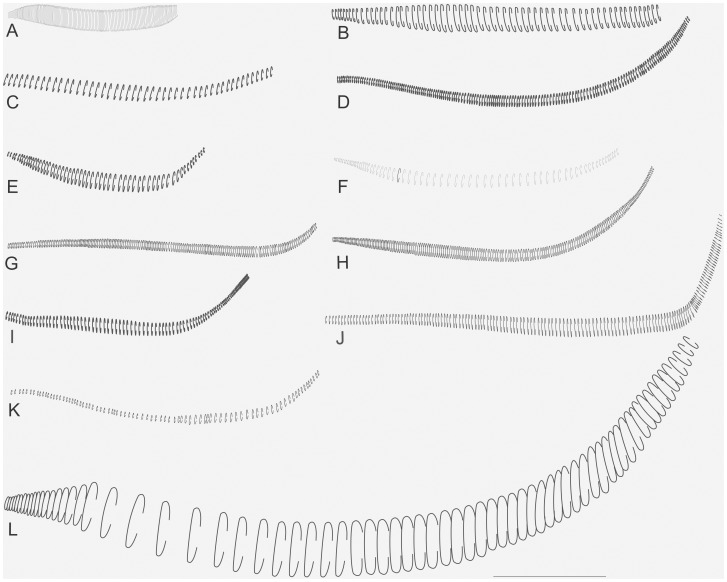
Stridulatory file. Diagrams showing the arrangement of the teeth. Hamayulini **trib. nov.**: (A) *Hamayulus rufomaculatus* **sp. nov.** B–K Listroscelidini: (B) *Cerberodon viridis* Perty, (C) *Cerberodon portokalipes* **sp. nov.**, (D) *Listroscelis carinata* Karny, (E) *Listroscelis angustifrons* **comb. nov.**, (F) *Listroscelis magnomaculata* **sp. nov.**, (G) *Listroscelis sooretama* **sp. nov.**, (H) *Listroscelis cohni* **sp. nov.**, (I) *Listroscelis fusca* **sp. nov.**, (J) *Listroscelis monnei* **sp. nov.**, (K) *Monocerophora minax* Walker, **reinstated status.** Terpandrini: (L) *Megatympanon speculatum* Piza. All diagrams are in the same scale = 0.5 mm.

The specimens were examined and compared under a Zeiss Stemi 2000-C stereomicroscope or a Zeiss Discovery V8. Whenever possible, we dissected the phallus of at least one male of each genus. We created diagrams arranged for pictorial documentation and comparison between genera and species: one plate showing spines and tubercles on sternum, coxae and trochanters (Fig. 2); one showing external components of the phallus (Fig. 3) for genera comparisons; a plate of male postabdomens (Fig. 4); male and female subgenital plates (Fig. 5); and stridulatory files (Fig. 6) for species comparisons. For each examined species, we elaborated a plate showing habitus of male and female in dorsal, lateral and frontal views (in some cases we had individuals of only one gender). On each plate, images of lateral and dorsal views are shown at the same scale, but the scale varies between plates. Images of frontal view on all plates are at the same scale for purposes of comparison. Diagrams and plates were edited using Corel Draw ×6. Descriptions and redescriptions include details of color patterns, important features for recognizing and characterizing Listroscelidinae [12].

We also examined specimens deposited in scientific collections, used to describe new species and for taxonomic revisions of this subfamily. Specimens were borrowed from UFES (one *Listroscelis angustifrons* (Piza) **comb. nov.**), MNRJ (three *Megatympanon speculatum* Piza, three *L. carinata* Karny and four *Cerberodon viridis* Perty) and IBB (*M. speculatum*). Type material of *L. angustifrons* **comb. nov.** and *M. speculatum* deposited in ESALQ were examined in a previous work [29]. We also had access to photographs of *Listroscelis* and *Monocerophora* species deposited at the NMW.

We created four maps showing the distribution of species studied in the present work. These records were the result of our own field collections (Fig. 1A–B) or obtained from literature.

### Nomenclatural acts

The electronic edition of this article conforms to the requirements of the amended International Code of Zoological Nomenclature, and the new names contained herein are available under that Code from the electronic edition of this article. This published work and the nomenclatural acts were registered in ZooBank, the online registration system for the ICZN. The ZooBank LSIDs (Life Science Identifiers) can be accessed through any standard web browser by appending the LSID to the prefix “http://zoobank.org/”. The LSID for this publication is: urn:lsid:zoobank.org:pub:FDC64475-A7BB-4B31-8D20-FE47224FE490*. The electronic edition of this work has been archived and is available from the following digital repositories: PubMed Central, LOCKSS and Orthoptera Species File Online (OSF).

### DNA preparation, amplification and sequencing

We extracted the total DNA of mid or hind leg muscles of each individual using an adjusted standard phenol–chloroform protocol [30]. A 1300 bp fragment of the COI gene and a 1600 bp fragment of the rRNA 18S were amplified by touchdown PCR [31]. PCR was performed using 25 µl reactions with 1 unit GoTaq Flexi DNA Polymerase (Promega), 1× GoTaq Flexi Buffer (Promega), 0.1 mM dNTP, 1.5 mM MgCl_2_, 0.2 mM of each primer (Table S1), 20 to 100 ng DNA, and ultra-pure water. For COI gene amplification, the following parameters were used: 5 min at 94°C initial denaturation; 10 cycles of 1 min at 94°C, 1 min 45 s at 55 to 45°C (touchdown), and 1 min 45 s at 72°C; 30 cycles of 1 min at 94°C, 1 min 45 s at 45°C, and 1 min 45 s at 72°C; with a final extension at 72°C for 5 min. For 18S gene amplification parameters were as follows: 5 min at 94°C initial denaturation; 15 cycles of 1 min at 94°C, 1 min 45 s at 60 to 45°C (touchdown), and 1 min 45 s at 72°C; 25 cycles of 1 min at 94°C, 1 min 45 s at 45°C, and 1 min 45 s at 72°C; with a final extension at 72°C for 5 min. PCR products were inspected with 1.5% agarose gel electrophoresis using GelRed (Biotium) to confirm amplification and verify contamination. Amplicons were sequenced with DNA sequencer Applied Biosystems 3730×l at the Macrogen Inc. (South Korea).

### Sequence evaluation and alignment

The resulting chromatograms were evaluated with the program Consed [32]. We aligned both sequences using Muscle [33], implemented in Mega 5.0 [34]. Since COI is coding DNA, we aligned their putative amino acid sequences and reversed them to get the nucleotide sequences. Sequences showing double peaks in the chromatogram and unexpected stop codons or gaps of one or two base pairs into the aligned putative amino acid sequence were eliminated. It is possible that these sequences are fragments of mitochondrial DNA that migrated to the nucleus (numts) [35]–[37], a very common phenomenon in Orthoptera [38]. Sequences were submitted to GenBank after these analyses (Table 3).

**Table 3:** Species treated in this work.

Locality	Species	M	F	I	COI	18S
1	*Monocerophora minax*	1	4	5	KJ524869	KJ420162–KJ420166
2	*Listroscelis monnei*		1		-	-
	*Listroscelis magnomaculata*	8	2	8	-	KJ420184–KJ420186
	*Monocerophora minax*	1	3	2	KJ524870–KJ524872	KJ420160
3	*Hamayulus rufomaculatus*	2	4	1	-	-
	*Monocerophora minax*	1	1	3	-	KJ420161
	*Hamayulus* sp.		1		-	KJ420191
4	*Listroscelis cohni*	1			-	KJ420183
	*Listroscelis monnei*	1	2		KJ524873–KJ524874	KJ420167–KJ420169
	*Hamayulus rufomaculatus*	3	5		KJ524886–KJ524893	KJ420187–KJ420190
	*Hamayulus* sp.			10	KJ524894–KJ524901	KJ420192–KJ420194
	*Monocerophora minax*			4	KJ524868	KJ420157–KJ420159
7	*Listroscelis carinata*		5	2	KJ524877–KJ524882	KJ420172–KJ420177
9	*Monocerophora* sp.			5	KJ524865–KJ524867	KJ420151–KJ420154
	*Listroscelis sooretama*	1	1		KJ524875–KJ524876	KJ420170–KJ420171
10	*Listroscelis fusca*	1	1		KJ524884–KJ524885	KJ420181–KJ420182
11	*Cerberodon portokalipes*	1	1		KJ524854–KJ524855	KJ420147
13	*Listroscelis itatiaia*		3	1	KJ524883	KJ420178–KJ420180
	*Monocerophora spinosa*		3	2	KJ524860–KJ524864	KJ420155–KJ420156
15	*Cerberodon viridis*		1	3	KJ524856–KJ524859	KJ420148–KJ420150
CELC	*Listroscelis angustifrons*	1			-	-
ESALQ	*Carliella mandibularis* *		1†		-	-
	*Listroscelis angustifrons*	1**			-	-
	*Megatympanon speculatum*	1***			-	-
IBB	*Megatympanon speculatum*	1			-	-
MNJR	*Cerberodon viridis*	3	3		-	-
	*Listroscelis carinata*	1	2		-	-
	*Megatympanon speculatum*	2	1		-	-
UFES	*Listroscelis angustifrons*	1			-	-

**Figure 7:**
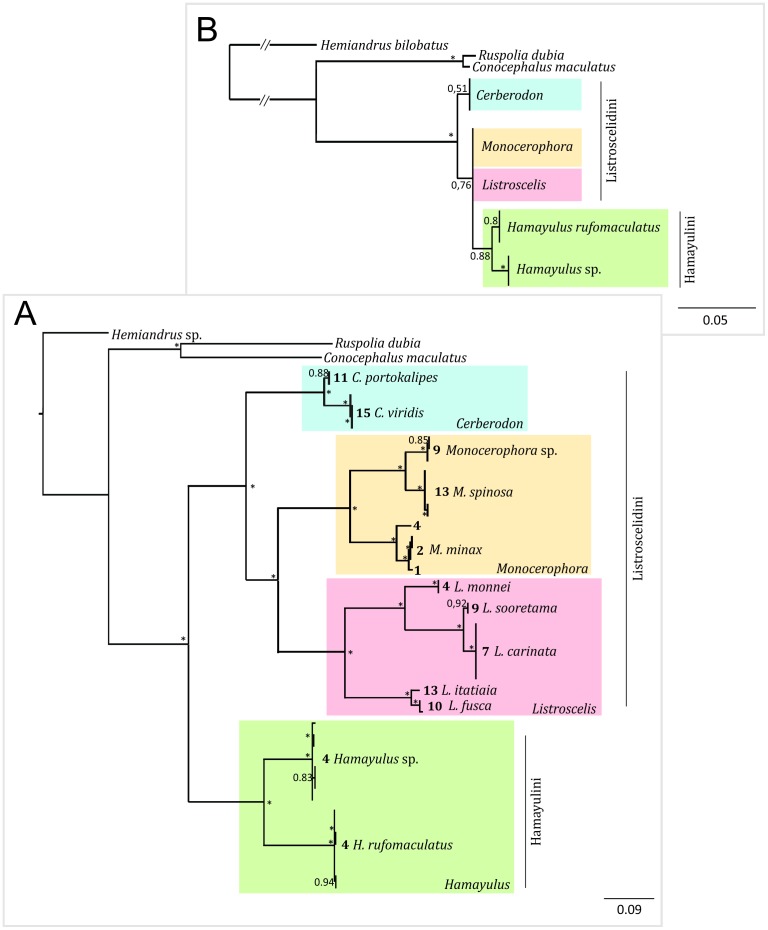
Listroscelidinae Bayesian phylogenetic inferences based on COI and 18S sequences. A) Bayesian consensus tree of 1290 aligned sites of 48 mitochondrial cytochrome oxidase I (COI) sequences (Table 3). Bold numbers on the left side of each species name correspond to the collection site of each specimen (Fig. 1). Outgroups and GenBank accession numbers (following classification in Eades et al. [14]): EU676747 = *Hemiandrus* sp. Ander (Stenopelmatoidea: Anostostomatidae); EF583824 = *Ruspolia dubia* (Redtenbacher) (Tettigonioidea: Conocephalinae: Copiphorini); NC016696 = *Conocephalus maculatus* (Le Guillou) (Tettigonioidea: Conocephalinae: Conocephalini). B) Simplified Bayesian consensus tree of 1615 aligned sites of 48 18S nuclear sequences. All *Listroscelis* and *Monocerophora* sequences were identical and were represented by few OTUs in the simplified tree (see the sequences numbers included in the complete tree in Table 3). Outgroups and GenBank accession numbers (following classification in Eades et al. [14]): EU676714 = *Hemiandrus bilobatus* Ander (Stenopelmatoidea: Anostostomatidae); JF792563 = *Ruspolia dubia* (Redtenbacher) (Tettigonioidea: Conocephalinae: Copiphorini); JF792565 = *Conocephalus maculatus* (Le Guillou) (Tettigonioidea: Conocephalinae: Conocephalini). In A and B, besides each ancestral node is a fraction number, representing its posterior probability; values >0.95 were represented by an asterisk. Listroscelidini and Hamayulini were represented in red and blue, respectively.

Number of males (M), females (F) and immatures (I) of each species captured in each conservation unit (the localities numbers refers to Fig. 1) or borrowed from each museum (acronyms). The columns COI and 18S contain the GenBank accession numbers of these sequences used in the molecular analyses (Fig. 7A–B), when applicable.

*Type material – examined by Juliana Chamorro-Rengifo for a previous work;

** holotype;

*** holotype and 2 paratypes;

† allotype.

### Analysis of molecular data

We analyzed both genes independently through Bayesian inference using the Markov Chain Monte Carlo (MCMC) method as implemented in MrBayes 3.1 [39], [40]. We ran MrModeltest version 2.0 [41] with PAUP* version 4.0b10 [42] to choose the model parameters to be estimated by Bayesian analyses using the Akaike Information Criterion (AIC) [43]. Two simultaneous and independent runs, each containing one cold and three heated chains, were processed for 10^7^ generations, each starting from a random tree (random topology and branch lengths) with random parameters. These parameters (topology, branch lengths and model parameters) were sampled every 1000 generations, resulting in 10^4^ samples. After running the analyses, we burned-out 25% of the initial generations, checked for chain convergence (<0.01) and used the remaining topologies (7500) with their respective branch lengths to build a majority-rule consensus tree (Fig. 7A–B).

### DNA Barcodes

Sequences suitable for DNA Barcoding should be short, have low intraspecific divergence and high interspecific divergence [44]. Thus, we examined our COI alignment for a region that could fulfill these requirements and built a distance tree using Neighbor-Joining [45] with Kimura 2-parameters model [46]. We compared this tree to the tree generated by Bayesian Analysis based on complete COI sequences.

## Results

### Morphological review

**Figure 8:**
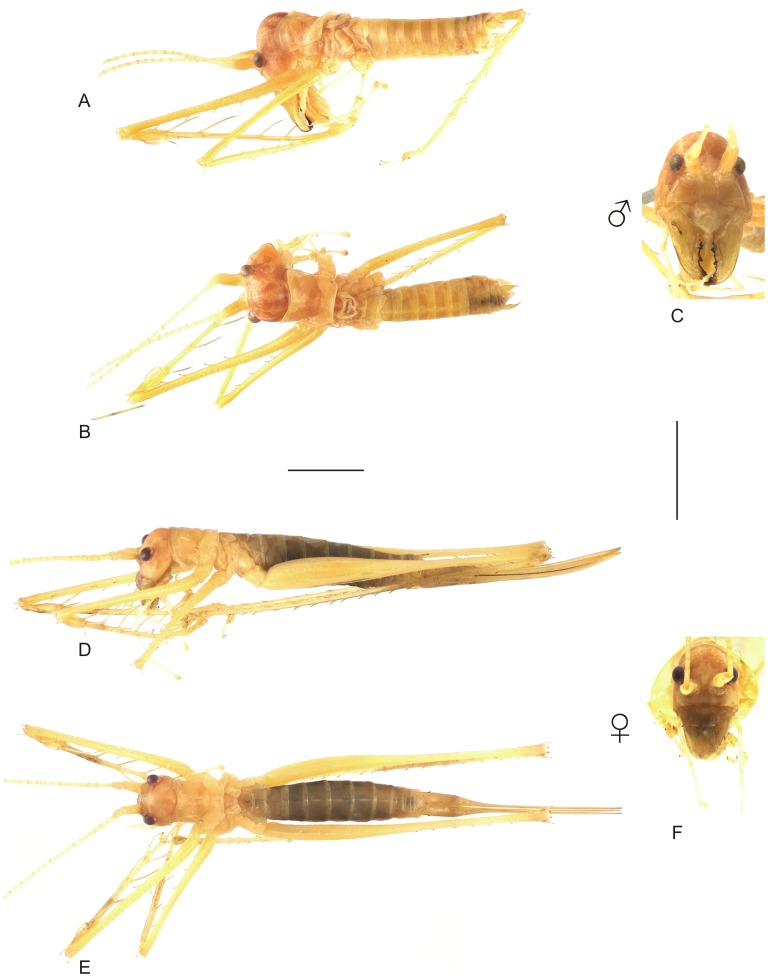
Habitus of *Hamayulus rufomaculatus* sp. nov. A–C Holotype male, (A) lateral view, (B) dorsal view, (C) frontal view. D–F Allotype female, (D) lateral view, (E) dorsal view, (F) frontal view. Scale bars for dorsal and lateral views (horizontal bar) and frontal views (vertical bar) = 0.5 mm.

**Figure 9:**
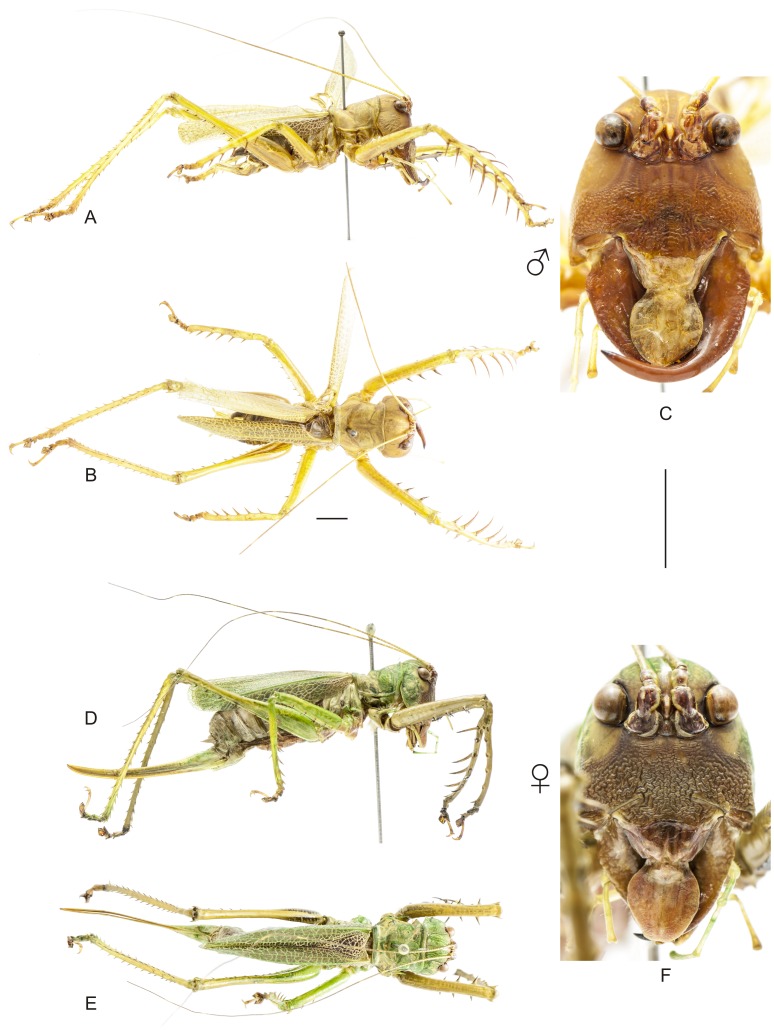
Habitus of *Cerberodon viridis* Perty. A–C Male (MNRJ) from Petrópolis, in the state of Rio de Janeiro, (A) lateral view, (B) dorsal view, (C) frontal view. D–F Female (CELC) from RPPN Bacchus, in the state of Rio de Janeiro, (D) lateral view, (E) dorsal view, (F) frontal view. Scale bars for dorsal and lateral views (horizontal bar) and frontal views (vertical bar) = 0.5 mm.

**Figure 10:**
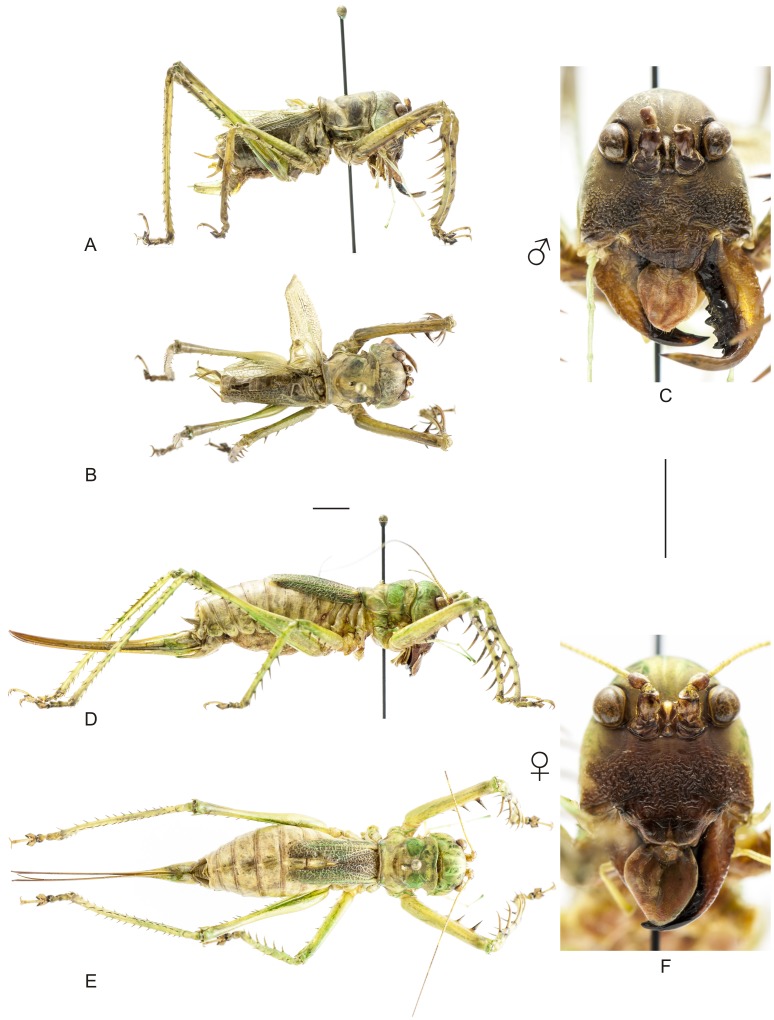
Habitus of *Cerberodon portokalipes* sp. nov. A–C Holotype male, (A) lateral view, (B) dorsal view, (C) frontal view. D–F Allotype female, (D) lateral view, (E) dorsal view, (F) frontal view. Scale bars for dorsal and lateral views (horizontal bar) and frontal views (vertical bar) = 0.5 mm.

**Figure 11:**
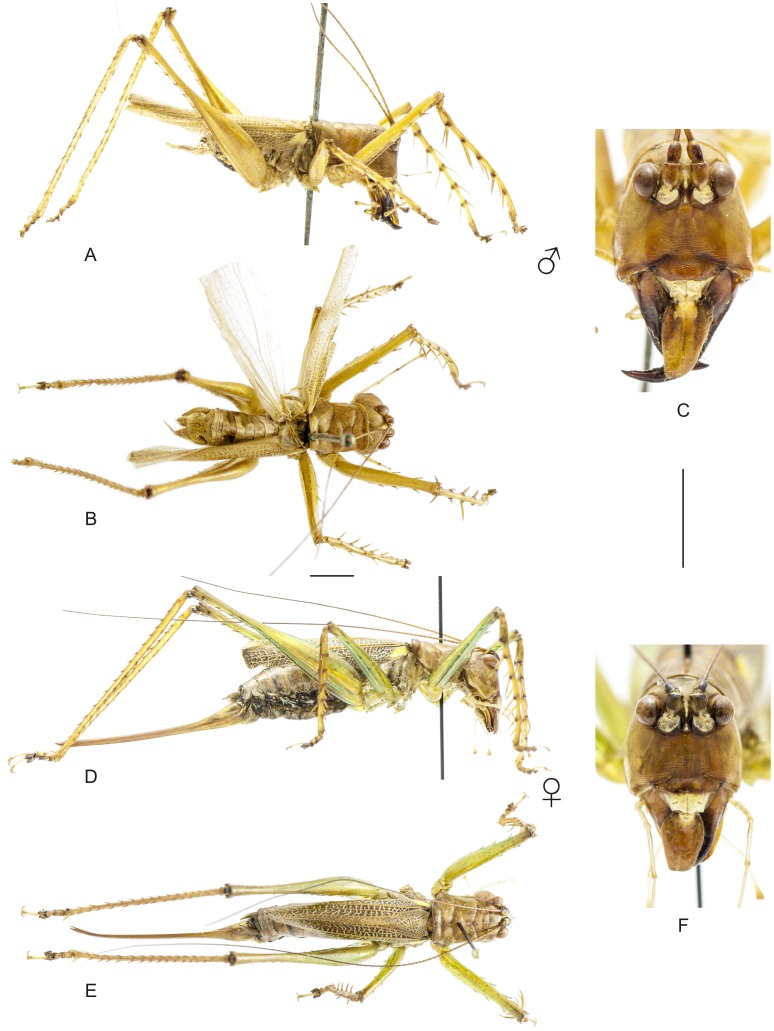
Habitus of *Listroscelis carinata* Karny. A–C Male (MNRJ) from Colatina, in the state of Espírito Santo, (A) lateral view, (B) dorsal view, (C) frontal view. D–F Female (CELC) from Parque Estadual do Rio Doce, in the state of Minas Gerais, (D) lateral view, (E) dorsal view, (F) frontal view. Scale bars for dorsal and lateral views (horizontal bar) and frontal views (vertical bar) = 0.5 mm.

**Figure 12:**
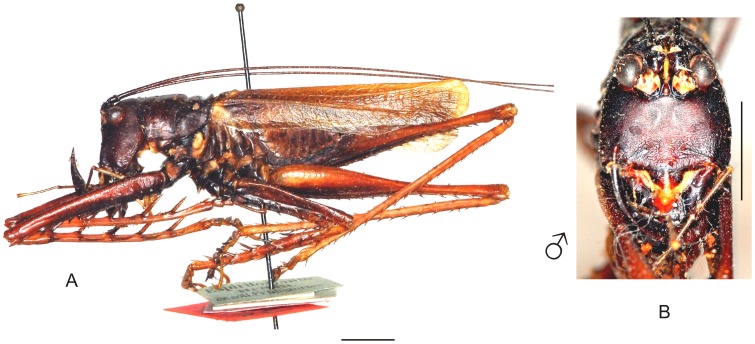
Habitus of *Listroscelis angustifrons* (Piza) comb. nov. A–C Male (NMW) from unknown locality in the state of Espírito Santo, (A) lateral view, (B) frontal view. Scale bars = 0.5 mm.

**Figure 13:**
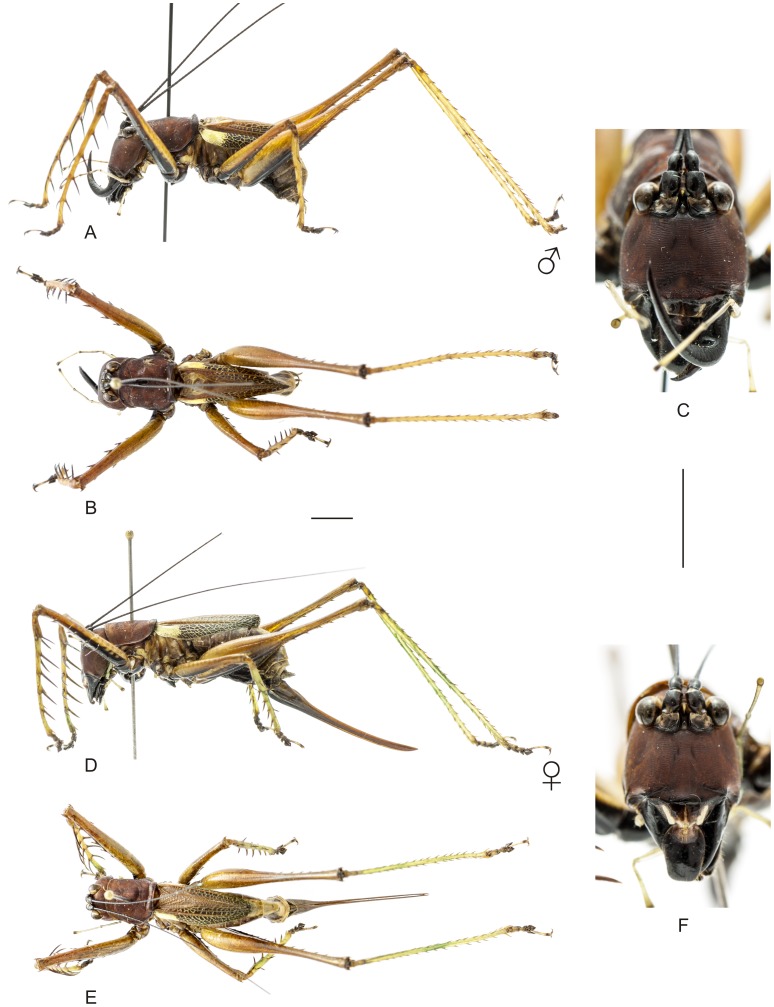
Habitus of *Listroscelis magnomaculata* sp. nov. A–C Holotype male, (A) lateral view, (B) dorsal view, (C) frontal view. D–F Allotype female, (D) lateral view, (E) dorsal view, (F) frontal view. Scale bars for dorsal and lateral views (horizontal bar) and frontal views (vertical bar) = 0.5 mm.

**Figure 14:**
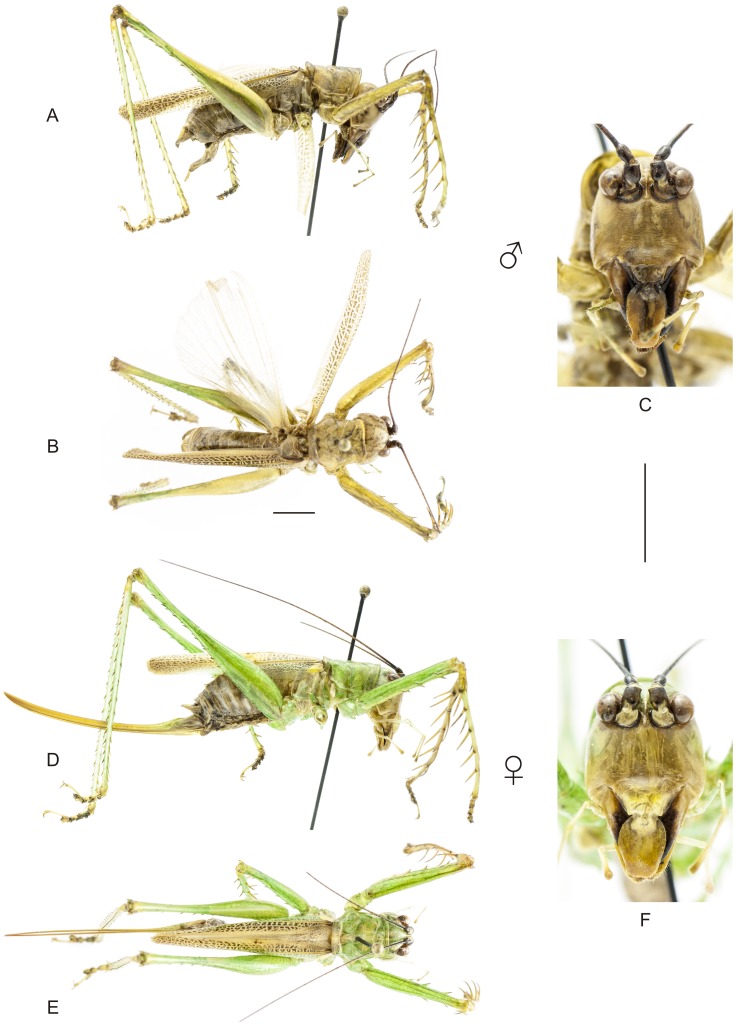
Habitus of *Listroscelis sooretama* sp. nov. A–C Holotype male, (A) lateral view, (B) dorsal view, (C) frontal view. D–F Allotype female, (D) lateral view, (E) dorsal view, (F) frontal view. Scale bars for dorsal and lateral views (horizontal bar) and frontal views (vertical bar) = 0.5 mm.

**Figure 15:**
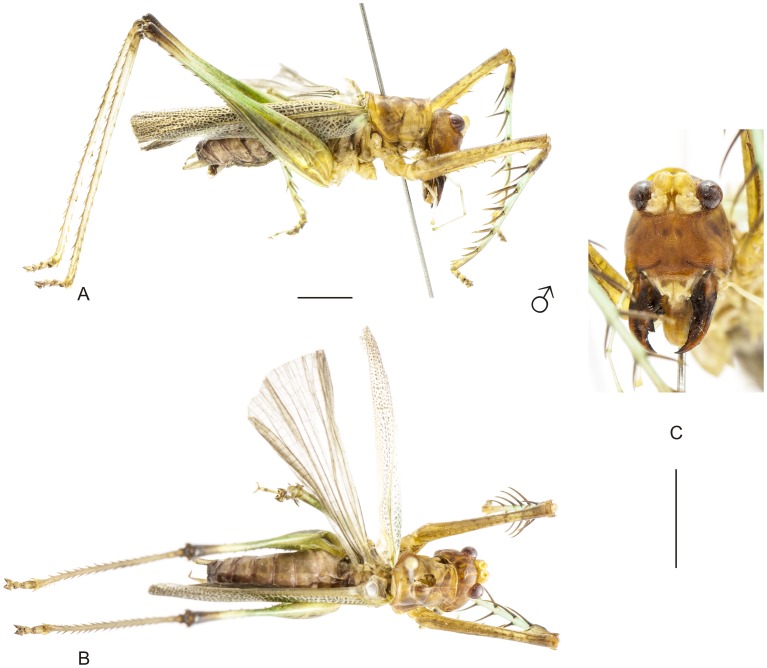
Habitus of *Listroscelis cohni* sp. nov. A–C Holotype male, (A) lateral view, (B) dorsal view, (C) frontal view. Body images in the same scale = 0.5 mm.

**Figure 16:**
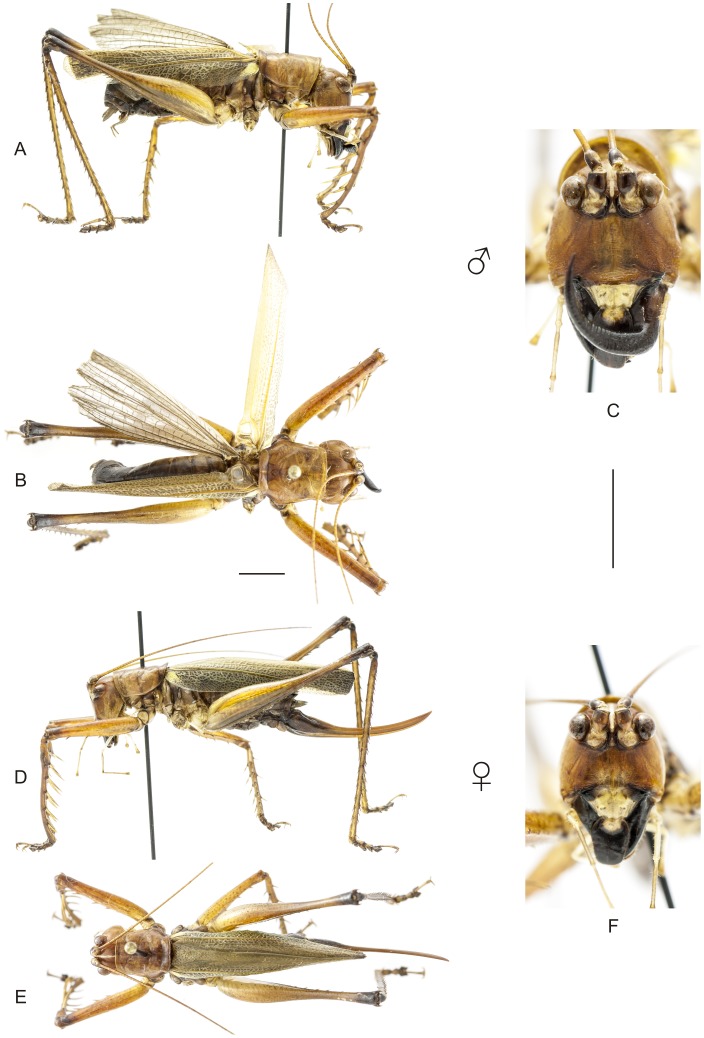
Habitus of *Listroscelis fusca* sp. nov. A–C Holotype male, (A) lateral view, (B) dorsal view, (C) frontal view. D–F Allotype female, (D) lateral view, (E) dorsal view, (F) frontal view. Scale bars for dorsal and lateral views (horizontal bar) and frontal views (vertical bar) = 0.5 mm.

**Figure 17:**
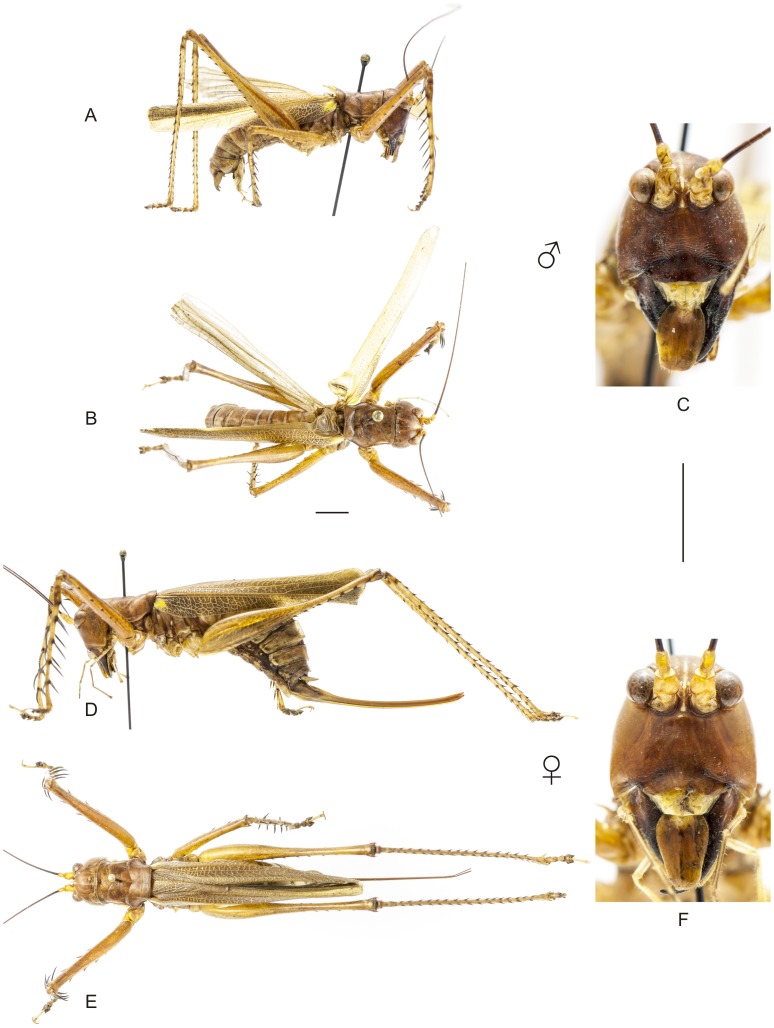
Habitus of *Listroselis monnei* sp. nov. A–C Holotype male, (A) lateral view, (B) dorsal view, (C) frontal view. D–F Allotype female, (D) lateral view, (E) dorsal view, (F) frontal view. Scale bars for dorsal and lateral views (horizontal bar) and frontal views (vertical bar) = 0.5 mm.

**Figure 18:**
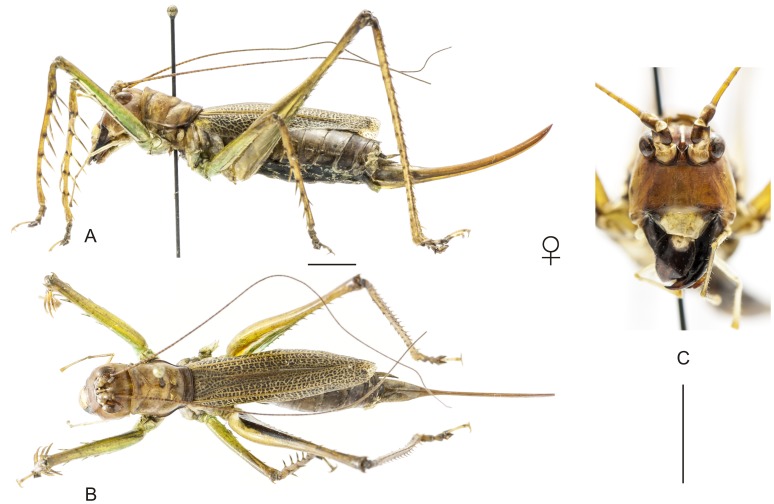
Habitus of *Listroscelis itatiaia* sp. nov. A–C Holotype female, (A) lateral view, (B) dorsal view, (C) frontal view. Scale bars = 0.5 mm.

**Figure 19:**
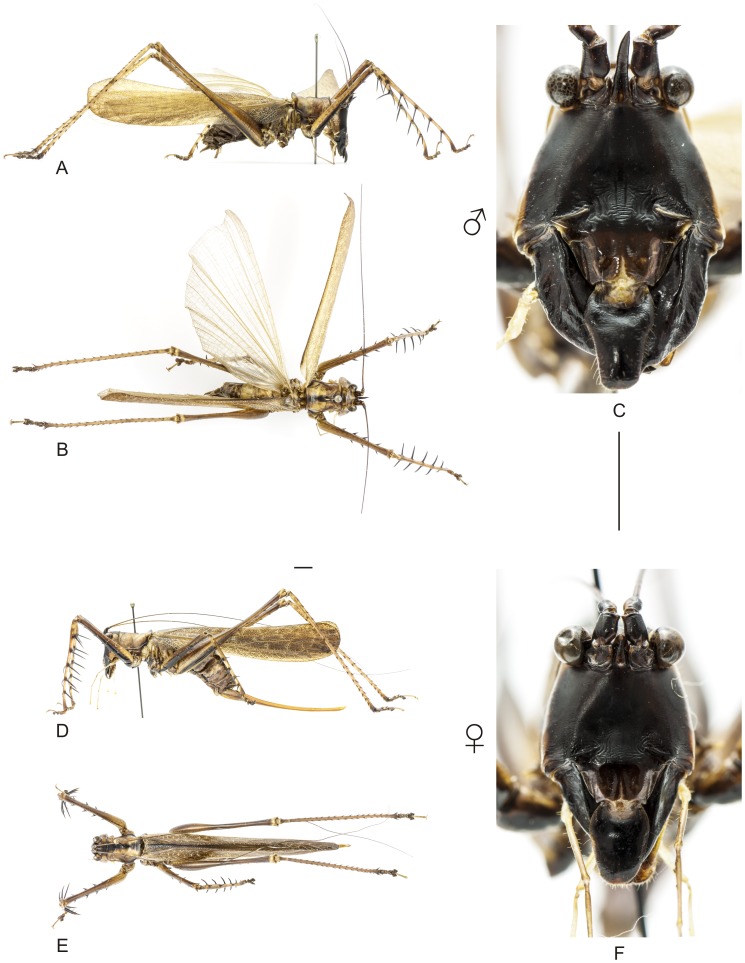
Habitus of *Monocerophora minax* Walker, reinstated status. A–C Male (CELC) from RPPN Serra do Teimoso, in the state of Bahia, (A) lateral view, (B) dorsal view, (C) frontal view. D–F Female (CELC) from RPPN Serra do Teimoso, in the state of Bahia, (D) lateral view, (E) dorsal view, (F) frontal view. Scale bars for dorsal and lateral views (horizontal bar) and frontal views (vertical bar) = 0.5 mm.

**Figure 20:**
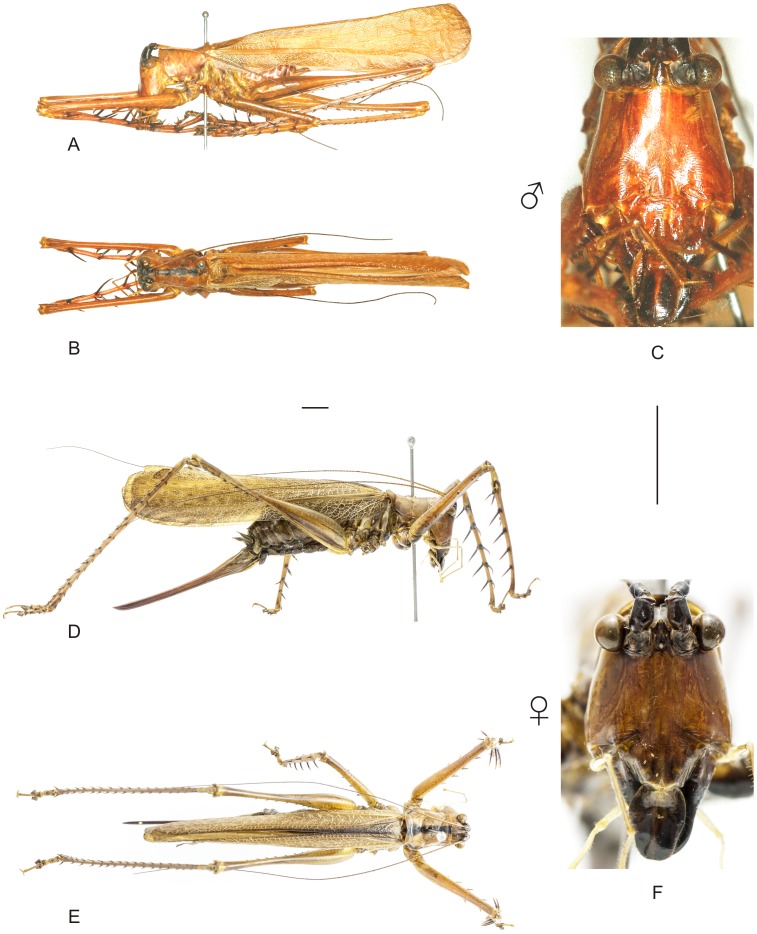
Habitus of *Monocerophora spinosa* (Karny). A–C Holotype male (NMW), (A) lateral view, (B) dorsal view, (C) frontal view. D–F Female (CELC) from Parque Nacional do Itatiaia, in the state of Rio de Janeiro, (D) lateral view, (E) dorsal view, (F) frontal view. Scale bars for dorsal and lateral views (horizontal bar) and frontal views (vertical bar) = 0.5 mm.

**Figure 21:**
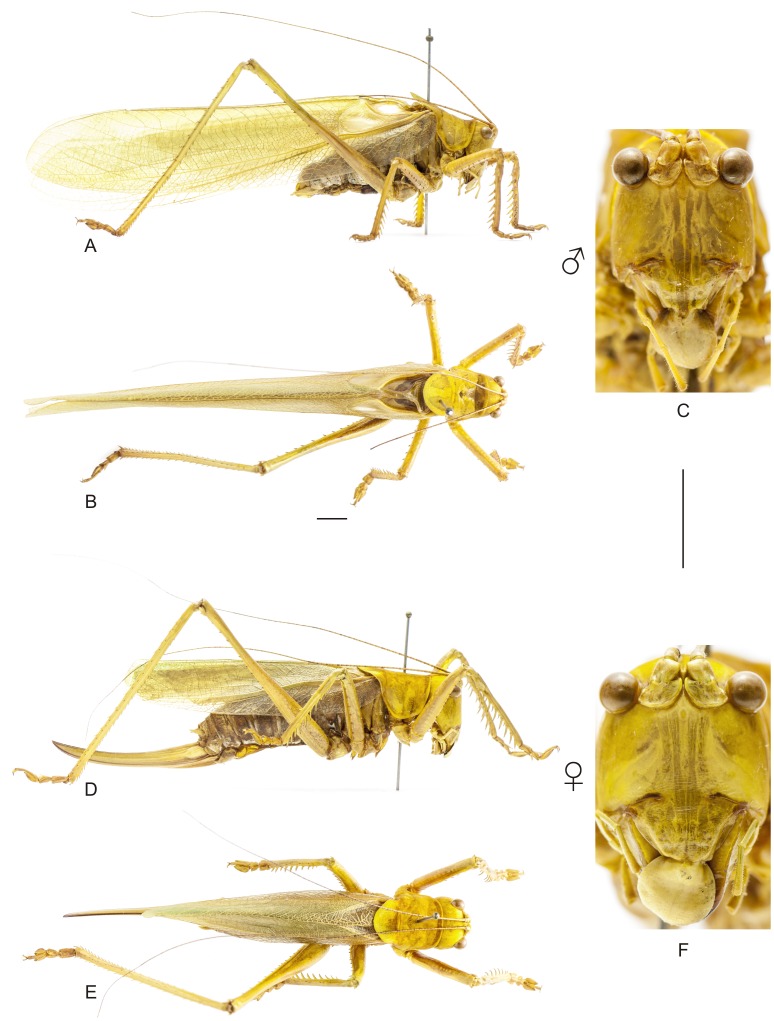
Habitus of *Megatympanon speculatum* Piza. A–C Male (MNRJ) from Petrópolis, in the state of Rio de Janeiro, (A) lateral view, (B) dorsal view, (C) frontal view. D–F Female (MNRJ) from Itaguaí, in the state of Rio de janeiro, (D) lateral view, (E) dorsal view, (F) frontal view. Scale bars for dorsal and lateral views (horizontal bar) and frontal views (vertical bar) = 0.5 mm.

**Figure 22:**
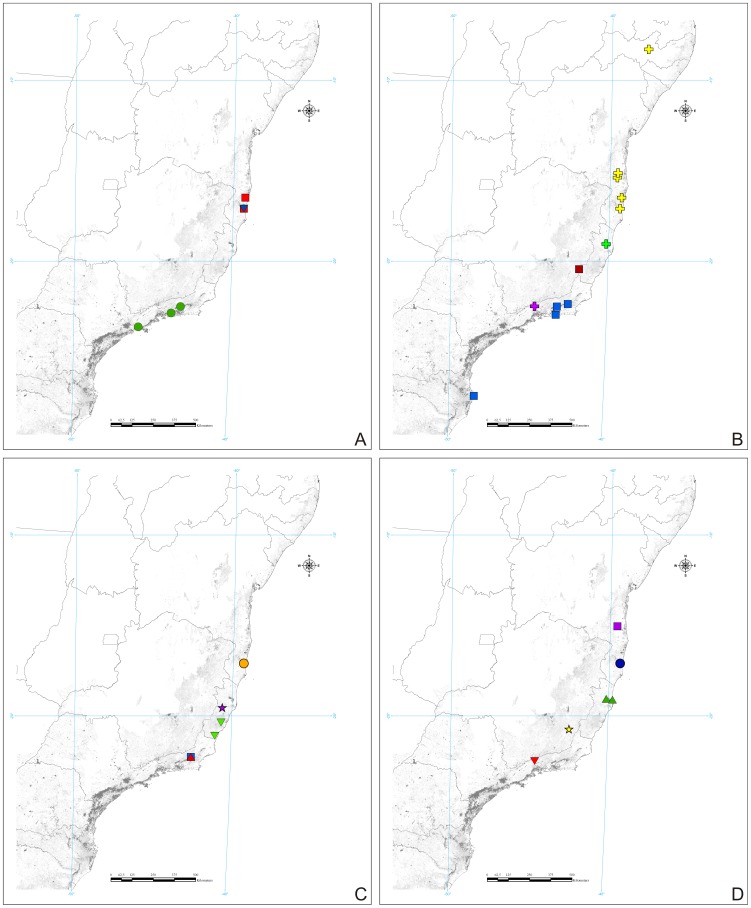
Geographic distribution of the species of Listroscelidinae in the Brazilian Atlantic Forest. (A) Registers of *Hamayulus rufomaculatus* **sp. nov.** (red square), *Hamayulus* sp. (blue inverted triangle) and *Megatympanon speculatum* Piza (green circle). (B) Registers of *Monocerophora minax* Walker, **reinstated status** (yellow plus), *M. spinosa* (Karny) (purple plus), *Monocerophora* sp. (green plus), *Cerberodon portokalipes* **sp. nov.** (dark red square) and *C. viridis* Perty (blue square). (C) Registers of *Listroscelis angustifrons* (Piza) (inverted green triangle), *L. atrata* Redtenbacher (blue square), *L. carinata* Karny (purple star), *L. cohni* **sp. nov.** (orange circle) and *L. ferruginea* Redtenbacher (red triangle). (D) Registers of *Listroscelis fusca* **sp. nov.** (yellow star), *L. itatiaia* **sp. nov.** (inverted red triangle), *L. magnomaculata* **sp. nov.** (purple square), *L. monnei* **sp. nov.** (dark blue circle) and *L. sooretama* **sp. nov.** (green triangle).

Table 3 summarizes all species studied in this work (Figs. 8–21), with the GenBank accession numbers for each gene, when applicable. We captured 104 individuals of Listroscelidinae in ten conservation units (Table 3, Fig. 1) in the Brazilian Atlantic Forest. Figure 22A–B shows records of species of all studied genera, except for *Listroscelis* species, which are shown in Fig. 22C–D. Using this material, we proposed a new tribe, recognized four genera (one new) and 14 morphospecies: eight new, four redescribed and two, *Hamayulus* sp. and *Monocerophora* sp., possibly new, but not described here due to the lack of adult males. In addition, we redescribed *Listroscelis angustifrons* **comb. nov.** and *Megatympanon speculatum* based on museum specimens. The second column of Table 1 summarizes the taxonomy proposed in this work.

### Molecular analysis

Figure 7A–B shows the gene trees based on COI and rRNA 18S, respectively.

#### Mitochondrial COI

The complete alignment of 48 COI gene sequences (Table 3) resulted in 1290 aligned sites, of which 540 are variable. The best fit model of sequence evolution using AIC was GTR + I + G. In Fig. 7A, after separating the outgroup (composed of other Tettigoniidae species) all sequences showed more differences between species, genera and tribes than within these taxa and all ancestor nodes were tightly defined (PP = 1.00), i.e., each taxon was represented by a well-defined cluster (Fig. 7A).

#### rRNA 18S

The complete alignment of 48 18S gene sequences (Table 3) resulted in 1615 aligned sites, of which only 11 were variable. The best fit model of sequence evolution using AIC was GTR + I. Among these sequences, several were identical, thus we chose to show a simplified 18S tree in Figure 7B. Unlike with COI, most taxa within Listroscelidinae were not well defined in this tree. After the separation of the outgroup, Listroscelidinae split into two poorly defined groups: the first containing the sequences of two species of *Cerberodon* (PP = 0.51) and the second containing the remaining sequences (PP = 0.76): a total of 36 identical sequences from four species of *Monocerophora* and six species of *Listroscelis* and a poorly defined cluster containing two well-separated species of *Hamayulus* **gen. nov.** (four sequences of *Hamayulus* sp. and four sequences of *H. rufomaculatus* **sp. nov.**).

### DNA Barcodes

Through visual analysis, we determined the 5′ portion of COI has higher interspecific than intraspecific variation. Thus, we delimited a fragment of 500 bp between sites 2394 and 2890 of the complete mitochondrial genome of *Oxya chinensis* (Thunberg) (Orthoptera: Acrididae, GenBank accession number: NC010219) (Fig. S5). The dendrogram obtained is similar to the one based on complete COI (Fig. 7A) and addresses the need to distinguish between species (Fig. S6).

### Taxonomy

**Hamayulini Fialho, Chamorro-Rengifo & Lopes-Andrade, trib. nov.**

#### Type-genus

*Hamayulus* Fialho, Chamorro-Rengifo & Lopes-Andrade, **gen. nov.**

#### Diagnosis

Species of this new tribe share the following combination of features: (i) body comparatively delicate, (ii) fastigium sulcated at the longitudinal midline, (iii) sclerites of antennal sockets not in contact at midline, (iv) thoracic auditory spiracle completely exposed, oval and small, free from pronotum, (v) spines on each sternite stout, and spines on meso- and metasternum flattened, (vi) wings short, partially exposing the second abdominal tergite, (vii) femora with ventral spines devoid of minute spines between them, (viii) fore tibiae weakly curved, (ix) tympana with rounded openings, (x) outer edge of fore tibiae with one spur below the tympanum, (xi) male paraprocts comparatively modified, enlarged, (xii) phallus completely membranous.

#### Included taxa

*Hamayulus* **gen. nov.**

This new tribe is proposed based on the morphological differences with the current recognized tribes, and supported by molecular data provided here (Fig. 7A).

***Hamayulus*** Fialho, Chamorro-Rengifo & Lopes-Andrade **gen. nov.** urn:lsid:zoobank.org:act:7E56D71A-2B2E-44DF-B11A-933AE08CC21B

(Figures 8, 22A, S1)

#### Etymology

The genus name is derived from Hamãy, considered a mother of the forest and protector spirit of the animals by Pataxós Indians. The Pataxós live mostly in Porto Seguro, type-locality of *Hamayulus rufomaculatus* **sp. nov.**

#### Type species

*Hamayulus rufomaculatus* Fialho, Chamorro-Rengifo & Lopes-Andrade, **sp. nov.**

#### Description

**Head.** Fastigium triangular, shorter than the first antennomere of the flagellum, dorsally flattened and bearing a linear sulcus at the longitudinal midline. Eyes oval and frontally protruding, each inserted next to the upper edge of the subjacent antennal socket. Head twice as long as wide; appearing globose in frontal view, mainly due to the protruding vertex. Frons triangular, with a conspicuous ocellus. Surface of clypeus, genae and face smooth. Mandibles robust and elongated, bearing a basal process at the cutting area. Maxillary and labial palpi elongate; maxillary palpi reaching the third abdominal sternite; last three palpomeres almost with of same length. **Thorax.** Pronotum with anterior margin slightly curved inward; posterior margin almost straight. Prozona with a transverse furrow, not extending to the lateral lobes. Mesozona without a transverse furrow. Metazona with a marked transverse furrow extending to the lateral lobe, reaching the lower margin. Lateral lobes with lower margin slightly curved, posterior margin completely curved; corners rounded and without sinus humeralis. Each sternite with two stout spines (Fig. 2A). Meso- and metasternum with flattened spines. **Legs.** Each coxa bearing one ventral spine, acute or rounded, at the basal and another at the distal portion, ventrally (Fig. 2A). Legs slender and elongated; hind femora longer than the body. Fore and mid femora without a ventral longitudinal furrow. Both ventral margins of femora armed with spines. Fore tibiae straight, each with a spine at the outer side of the dorsum, below the tympanum. Hind tibiae with spines on the dorsal and ventral margins. Genicular lobes of legs ending in an acute tip. Fore tibiae with tympanal openings located dorsally; sclerites completely inflated; openings about three times as long as wide. **Male postabdomen**. Paraprocts elongated (Fig. 4A). Subgenital plate wide; apical portion emarginated (Fig. 5A). Phallus membranous, devoid of titillator (Fig. 3A). **Female postabdomen.** Subgenital plate three times longer than wide; apical portion triangular, without emargination (Fig. 5B). Ovipositor shorter than the length of the body, mostly straight; upper valve as wide as lower valve; apex of ovipositor acute (Fig. 8D).

This new genus includes *H. rufomaculatus* **sp. nov.** and a second species that is known only from immatures (one female and seven males) and therefore not described here. This second *Hamayulus* species was recognized based on molecular data (Fig. 7A–B).

***Hamayulus rufomaculatus*** Fialho, Chamorro-Rengifo & Lopes-Andrade **sp. nov.** urn:lsid:zoobank.org:act:6328C346-748D-481C-AD72-CC453C0F4119

(Figures 8, 22A, S1)

#### Etymology

The specific epithet refers to the red spots on the last two abdominal sternites.

#### Diagnosis

This species can be distinguished by the following combination of characters: (i) body mostly greenish, (ii) tegmina short, covering only the meso- and metanotum, (iii) stridulatory file and apical portion of each tegmen with a red mark, (iv) last two abdominal tergites with a red mark at the midline of about one-third the length of the tergite, (v) male paraprocts modified as an elongated structure with an acute tip.

#### Description (holotype ♂ & allotype ♀)

Holotype **♂**: TL 18.00; PL 3.00; PW 3.00; EyeW 2.00; TegL 1.30; SL 0.83; NT 161; minT 0.01; maxT 0.10; HF 18.00; HT 23.00; sFF, inner margin, large 4, outer large 6; sMF, inner margin, large 5, outer large 5; sHF, inner margin, large 11, outer large 14; sMTld 5; sHTd, inner margin 19, outer 21; sHTv, inner margin 11–25, outer 10–21. Allotype ♀: TL 17.00; PL 4.00; PW 3.00; EyeW 3.00; TegL 1.60; HF 20.00; HT 24.00; sFF, inner margin, large 4, outer large 7; sMF, inner margin, large 6, outer large 7; sHF, inner margin, large 14, outer large 15; sMTld 5; sHTd, inner margin 20–19, outer 22–20; sHTv, inner margin 12, outer 12; OL 12.00. **Head.** Antennal scape twice as long as pedicel. Antennae inserted at the middle of the face in frontal view. Frons subtriangular and slightly inflated. Clypeus short, about one-quarter the length of the labrum. Mandibles with blackish cutting edge. **Wings.** Tegmina with stridulatory file as shown in Fig. 6A. **Legs.** Femora bearing thick bristles over the entire dorsal surface, ventrally with spines decreasing in length from base to apex. Fore tibiae with nine spines in male and five in female. Tarsi with the fourth tarsomere as long as the first three together. **Male postabdomen.** Cerci cylindrical (Fig. 4A), width decreasing from middle to apex; apical portion narrower, with tubercles and more bristles than basal portion; tip rounded. Styli short, about one-quarter the length of the subgenital plate (Fig. 5A). Phallus as shown in Fig. 3A. **Female postabdomen**. Ovipositor slightly shorter than abdomen (Fig. 8D). **Coloration.** Body mostly greenish when alive (Fig. S1; after death specimens were stored in alcohol and lost the natural color). Male cerci reddish. Ovipositor with a blackish stripe at the outer lateral surface. **Alive,** individuals with eyes, abdominal sternites and lateral portion of last two abdominal tergites yellowish. Sclerites of tympana and tegmina reddish. Females are devoid of reddish spots at the abdominal apex (Fig. S1).

#### Variation

Measurements of males (n = 4, including the holotype): TL 15.00–19.00; PL 3.00; PW 2.00–3.00; EyeW 2.00; TegL 1.30–2.00; HF 15.00–20.00; HT 20.00–24.00; sFF, inner margin, large 4, outer large 6; sMF, inner margin, large 5–7, outer large 5–6; sHF, inner margin, large 11–12, outer large 14; sMTld 5; sHTd, inner margin 19–23, outer 21–22; sHTv, inner margin 11–25, outer 10–21. Measurements of females (n = 6, including the allotype): TL 16.00–21.00; PL 2.50–4.00; PW 2.50–3.00; EyeW 2.00–3.00; TegL 1.00–1.60; HF 18.00–20.00; HT 22.00–24.00; sFF, inner margin, large 4–5, outer large 5–7; sMF, inner margin, large 6, outer large 7; sHF, inner margin, large 11–14, outer large 13–15; sMTld 5; sHTd, inner margin 19–21, outer 18–22; sHTv, inner margin 12–15, outer 12–17; OL 12.00–15.00.

#### Type series

**Holotype male** (CELC) labeled \Brasil, BA, Porto Seguro, P.N. Pau Brasil. 4–6, II, 2012. J. Chamorro *leg*. [handwritten on white paper] \4/Listro/Pau [printed on white paper]\ *Hamayulus rufomaculatus* Fialho, Chamorro-Rengifo & Lopes-Andrade [handwritten on red paper]\. **Allotype female** (CELC) labeled \Brasil, BA, Porto Seguro, P.N. Pau Brail. 4–6, II, 2012. J. Chamorro *leg*. [handwritten on white paper] \1/Desc/Pau [printed on white paper]\ *Hamayulus rufomaculatus* Fialho, Chamorro-Rengifo & Lopes-Andrade [handwritten on blue paper]\. **Paratypes**: three adult males (CELC) labeled\Brasil, BA, Prado, P.N. Descobrimento. 13–15, I, 2012. J. Chamorro *leg*. [printed on white paper]\, additionally with the respective codes \14\, \22\, \16\ and labeled \listro/Desc [handwritten on white paper]\ *Hamayulus rufomaculatus* Fialho, Chamorro-Rengifo & Lopes-Andrade [handwritten on blue paper]\. Six females (CELC) labeled as follows: one adult female\Brasil, BA, Porto Seguro, P.N. Pau Brail. 4–6, I, 2012. J. Chamorro *leg*. [handwritten on white paper] \8/listro/Pau [printed on white paper]\; one immature female \6/listro/Pau\; four adult females\Brasil, BA, Prado, P.N. Descobrimento. 13–15, I, 2012. J. Chamorro *leg*. [handwritten on white paper]\codes: \5\, \15\, \17\ and \18/listro/Des [printed on white paper]\, and additionally labeled \*Hamayulus rufomaculatus* Fialho, Chamorro-Rengifo & Lopes-Andrade [handwritten on blue paper]\.

#### Distribution

The specimens were sampled in Parque Nacional (stands for National Park in Portuguese; abbreviated P.N. or PARNA) do Pau Brasil, PARNA do Descobrimento, localities 3 and 4 respectively (Figs. 1, 22A).

***Hamayulus*** **sp.**

(Figure 22A)

The distinction of *Hamayulus* sp. was based in molecular results (Fig. 7A–B). The sampled individuals are all immature and, therefore, morphological identification is not possible at this moment.

#### Specimens examined

Seven immature males (CELC) \Brasil, BA, P.N. do Descobrimento. J. Chamorro *leg*. 13–15, I, 2012 [printed on white paper] \*Hamayulus* sp. [handwritten on white paper]\, with the respective codes \7\, \9\, \10\, \13\, \19\, \20\, \24\, and additionally labeled \Listro/Descobrimento [printed on white paper]\. One immature female (CELC) \Brasil, BA, P.N. do Descobrimento. J. Chamorro *leg*. 13–15, I, 2012 [printed on white paper] \*Hamayulus* sp. [handwritten on white paper]\25\Listro/Descobrimento [printed on white paper]\.

#### Distribution

The specimens were sampled in PARNA do Descobrimento, locality 4 (Figs. 1, 22A).

**Listroscelidini Redtenbacher, 1891**

#### Type-genus

*Listroscelis* Serville, 1831

#### Diagnosis

The genera belonging to this tribe share the following combination of features: (i) body usually robust, (ii) fastigium laterally compressed, narrower and shorter than the first antennomere of the flagellum, usually sulcated (projecting upward and not sulcated in *Monocerophora*), (iii) antennal sockets usually not in contact at midline, (iv) thoracic auditory spiracle completely exposed, oval and enlarged, free from pronotum, (v) pro- and mesosternum with slender spines, usually flattened at the metasternum, (vi) tegmina covering at least the first two abdominal tergites, (vii) ventral portion of femora with large spines usually interspaced with minute ones, (viii) fore tibiae notably curved, (ix) tympana with elongated and wide openings, with (x) two dorsal rounded pits below each tympanal opening, one at the inner and another at the outer edge of the tibia and, (xi) male paraprocts semitriangular, wide, concealing the membranous portion of the phallus, with lateral outer corners of each plate with a down curved spine (arrows in Figure 4), (xii) titillator usually consisting of a longitudinal sclerite attached to the dorsal lobe of the phallus, with two projections protruding outwards.

#### Included taxa

*Carliella*, *Cerberodon*, *Isocarliella* Mello-Leitão, *Listroscelis*, *Macrometopon* Bruner and *Monocerophora*.

***Cerberodon*** **Perty, 1832**

(Figures 9, 10, 22B, S2)

**Type species:** *Cerberodon viridis* Perty, 1832

#### Redescription

**Head.** Eyes globose and frontally prominent, inserted separately from the ventral margin of antennal sockets. Antennal sockets separated by half the maximum width of the eyes. Head globose and wide in frontal view, due to the protruding vertex; about as wide as long in dorsal view. Frons subtriangular, with a tiny rounded projection in the middle of the longitudinal midline, where the inconspicuous ocellus is located (ocellus with the same color as the frons). Face, genae and basal portion of clypeus strongly wrinkled, with a brain-like appearance. Mandibles robust, with elongated apex; in male, the apical portion of the left mandible is strongly angulated, elongated and curved upwards. Mandibles bear a basal process at the cutting edge. Maxillary and labial palpi greenish. **Thorax**. Pronotum with anterior margin slightly curved inward, posterior margin almost straight. Prozona with a transverse furrow extending to the lateral lobe, reaching the anterior margin. Mesozona with a straight transverse furrow which is strongly curved forward on the lateral lobes; in lateral view this furrow forms a strong dorsal depression. Metazona with a transverse furrow extending to the lateral lobe, reaching the lower margin. Lateral lobes straight, except for their posterior portions; posterior margin slightly slanted; posterior corners rounded, without sinus humeralis. Each sternite with two stout spines, the ones on the metasternum flattened (Fig. 2B). **Legs.** Wings fully developed or shortened; tegmina devoid of distinctive marks. Coxae usually with an acute or rounded spine on the basal portion (Fig. 2B). Trochanters usually with an acute spine on the apical portion. Legs robust and short; hind femora shorter than body length. Fore and mid femora with a wide longitudinal ventral furrow. Both ventral margins of femora armed with long spines interspersed with more delicate ones. Fore tibiae slightly curved inwards, with a small spur or pit (when the spur is lost) dorsally, below and close to each tympanal opening. Mid tibiae with four to seven spines. Hind tibiae with spines on their dorsal and ventral margins. Genicular lobes of all legs ending in an acute spine. Tympanal openings located at the outer edge of tibiae; entire region of the tympana slightly inflated, with elongated tympanal openings, the width one-eleventh the length. **Male postabdomen.** Cerci bent in a somewhat acute angle, ending in a hook-like acuminate tip (Fig. 4B–C); apical portion as long as basal portion, with tubercles and long bristles. Supraanal plate triangular or rounded. Subgenital plate wide; apical portion with a V-shaped emargination (Fig. 5C, E). Styli short, about one-quarter the length of the plate or shorter. Phallus with prominent titillator, visible from dorsal and lateral views (Fig. 3C–D), projected outwards, ending in a rough lobe. The titillator is composed by two independent bars with only their bases attached to the dorsal lobe; at the midline, the titillator is close to two transversal secondary sclerites that form a kind of bridge, but are not fused (Fig. 3B). **Female postabdomen**. Subgenital plate triangular or subtriangular, with a V-shaped emargination of about one-quarter the length of the plate at the longitudinal midline (Fig. 5D, F). Ovipositor shorter than body length, mostly straight; upper valve about three times as wide as lower valve; apex of ovipositor acute (Figs. 9D, 10D).

#### Included species

This genus includes *C. viridis* Perty, 1832 and *C. portokalipes* **sp. nov.**

#### Excluded species

*Cerberodon angustifrons* Piza, 1960, transferred to *Listroscelis*; and *Cerberodon cuiabensis* Piza, 1982, new synonym of *Carliella mandibularis* Karny, 1911.

***Cerberodon viridis*** **Perty, 1832**

(Figures 9, 22B, S2)

#### Diagnosis

This species can be distinguished from *C. portokalipes* by the following combination of characters: (i) tegmina longer than abdomen, (ii) fore tibiae with ventral surface blackish.

#### Redescription

(Based on images of type material and examination of additional specimens). **Head.** Frons subtriangular and protruding (Fig. 9C, F). Mandibles with outer margin wrinkled. **Wings.** Tegmina with stridulatory file as shown in Fig. 6B. **Male postabdomen.** Cerci with basal portion wider than apical portion, strongly curved inwards and ending in an acute tip (Fig. 4B). Subgenital plate with a deep V-shaped emargination shorter than half the length of the plate (Fig. 5C). Phallus as shown in Fig. 3C–D. **Female postabdomen.** Subgenital plate with a V-shaped emargination (Fig. 5D) of about one-quarter the length of the plate. Ovipositor as long as abdomen (Fig. 9D). **Coloration.** Body mostly light greenish and dark brownish. Head dorsally greenish. Fastigium dark brownish. Scape and pedicel ventrally chestnut-colored and dorsally greenish; flagellum light brownish. Eyes light brownish, with cuticle around them blackish. Frons dark brownish. **Alive**, surfaces described as dark brownish or chestnut-colored are dark reddish (face, anterior and posterior margin of pronotum, spines on legs, mouthparts in ventral view, areas at sternum, abdominal spiracles, abdominal sternites, dorsal and ventral margin of ovipositor). Eyes pinkish frontally and whitish dorsally; labrum pinkish, lighter than after dead. Face, genae and clypeus dark brownish. Mandibles light brownish with cutting edge dark. Labrum brownish. Mouthparts in ventral view dark brownish. Pronotum greenish. Tegmina with primary and secondary veins greenish, surface between veins dark brownish, anal and stridulatory area dark brownish. Legs mostly greenish with spines dark brownish; coxae and trochanters chestnut-colored ventrally, with remaining surface greenish. Fore legs with ventral surface of femora and surface close to spines of tibiae blackish. Tarsi and tarsal claws dark brownish. Sternum greenish, with some chestnut-colored spots. Tergites greenish; sternites in male light reddish, and in female whitish (Fig. S2A–F).

#### Variation

Measurements of males (n = 2): TL 33.00; PL 8.00; PW 7.00; EyeW 5.00; TegL 26.00–27.00; SL 2.71–2.78; NT 65–67; minT 0.02; maxT 0.18–0.19; HF 22.00–23.00; HT 24.00–26.00; sFF, inner margin, large 7, small 9–13, outer large 6–7, small 2–19; sMF, inner margin, large 6–7, small 11–12, outer large 6–7, small 17–24; sHF, inner margin, large 10–14, small 0–2, outer large 9–14, small 0–3; sMTld 1–4; sHTd, inner margin 12–14, outer 16–; sHTv, inner margin 13–17, outer 11–13. Measurements of females (n = 5, including the allotype): TL 36.00–42.00 (including tegmina); PL 7.00–9.00; PW 7.00; EyeW 4.00–5.00; TegL 20.00–27.00; FF 15.00–16.00; FT 16.00–18.00; HF 22.00–24.00; HT 25.00–28.00; sFF, inner margin, large 6, small 0–3, outer large 6–7, small 0–4; sMF, inner margin, large 6, small 14–20, outer large 6–7, small 18–23; sHF, inner margin, large 11–13, small 0, outer large 9–12, small 0; sMTld 3–4; sHTd, inner margin 13–14, outer 15–17; sHTv, inner margin 14–17, outer 11–12; OL 25.50–28.00.

#### Specimens examined

Four specimens (CELC) \Brasil, RJ, RPPN Bacchus, 16–19, XI, 2011, J. Chamorro *leg*. [handwritten on white paper] \*Cerberodon viridis* [handwritten on white paper]\, and additionally labeled as follows: one adult female \3/Listro/Bacchus [printed on white paper]\, one immature male \4/Listro/Bacchus [printed on white paper]\, two immature females \1 and 2/Listro/Bacchus [printed on white paper]\. One male (MNRJ) \No. R. Anlé. Petrópolis. 1936 [handwritten on yellow paper] \*Cerberodon viridis* [handwritten on white paper]\; two males (MNRJ) \Petrópolis 1.52. Frey Thomaz [handwritten on blue paper] no verso Inst. Osvaldo Cruz [handwritten on yellow paper]\; one female (MNRJ) \Vista chinesa 79. col. OTERO [handwritten on yellow paper] \*Cerberodon viridis* [handwritten on white paper]\; two females (MNRJ) \Petrópolis. Est. do Rio. BRASIL. [typewritten] janeiro 1958. Herta [handwritten] \COLECÃO CAMPOS SEABRA [typewritten on white paper]\.

#### Distribution

The type-locality is “Brasilia aequatoriale”, but the exact locality is unknown. Based on new records and literature data, this species is distributed in the Atlantic Forest from Rio de Janeiro to Santa Catarina [21]. The specimens examined here were sampled in Reserva Particular do Patrimônio Natural (RPPN) Bacchus, locality 15 (Figs. 1B, 22B) or borrowed from the MNRJ.

***Cerberodon portokalipes*** Fialho, Chamorro-Rengifo & Lopes-Andrade **sp. nov.** urn:lsid:zoobank.org:act:E8F5BBA5-7DCD-4375-BCE9-343C406169F2

(Figures 10, 22B, S2)

#### Etymology

The specific epithet refers to the dark orange color on the ventral portion of the fore tibia.

#### Diagnosis

This species can be distinguished from *C. viridis* by the following combination of characters: (i) tegmina shorter than abdomen, not covering the last two abdominal segments in males, and not covering the last five abdominal segments in females, (ii) fore tibiae with ventral surface dark brownish when dead, dark orange when alive.

#### Description (holotype ♂ & allotype ♀)

Holotype **♂**: TL 27.00; PL 9.00; PW 7.00; EyeW 4.00; TegL 14.00; SL 2.39; NT 66; minT 0.04; maxT 0.08; HF 22.00; HT 26.00; sFF, inner margin 6, outer 6, small 0–2; sMF, inner margin, large 5, small 19, outer large 5, small 6; sHF, inner margin, large 8–10, small 2–8, outer large 10–12, small 0–1; sMT, inner margin 7, outer 4; sMTld 4; sHTd, inner margin 10–15, outer 12–13; sHTv, inner margin 9–12, outer 12. Allotype ♀ TL 35.00; PL 8.00; PW 8.00; EyeW 4.00; TegL 13.00; HF 22.00; HT 24.00; sFF, inner margin, large 5–6, small 2–3, outer large 5–6, small 1–2; sMF, inner margin, large 19, small 18, outer large 4, small 4; sHF, inner margin, large 11–13, small 0, outer large 11–12, small 0; OL 28.00. **Head.** Frons subtriangular, with an oval protruding tubercle in the middle where the ocellus is located (Fig. 10C, F). Mandibles with wrinkled outer margin. **Wings.** Tegmina with stridulatory file as shown in Fig. 6C. **Male postabdomen**. Cerci with basal portion wider than apical portion, which is flattened dorsoventrally, strongly curved inwards and ending in an acute tip (Fig. 4C). Subgenital plate with a deep V-shaped emargination (Fig. 5E) with half the length of the plate. **Female postabdomen.** Female subgenital plate with a V-shaped emargination (Fig. 5F) one-quarter the length of the plate. Ovipositor as long as abdomen (Fig. 10D). **Coloration.** Body coloration mostly light greenish and dark brownish. Head with dorsal surface light greenish. Fastigium dark brownish. Antennal scape dark brownish. Pedicel with frontal surface dark brownish and dorsal surface greenish. Flagellum with greenish antennomeres. Eyes dark brownish, with cuticle around the eyes blackish. Frons dark brownish, with a whitish ocellus. Face, genae and clypeus dark brownish. Mandibles light brownish with dark cutting edge. Clypeus dark brownish with darker apical portion. Labrum pinkish with basal portion dark brownish. Ventral portion of mouthparts dark brownish. Pronotum greenish. Tegmina with primary and secondary veins light greenish, surface between veins dark brownish; stridulatory area dark. Fore coxae with basal portion greenish and apical portion reddish; trochanters dark brownish. Fore femora greenish; lower margin reddish; spines whitish with inner base blackish. Fore tibiae greenish with darker base; spines light greenish. Mid femora greenish, lower margin and spines whitish, ventral surface greenish. Hind femora greenish, lower margin lighter, spines dark brownish. Hind tibiae greenish with light brownish spines. Tarsi dark brownish, the third one darker than the others; claws greenish with dark tip. Sternum mostly greenish, prosternum light greenish; meso- and metasternum with posterior margin reddish. Tergites dark brownish; sternites a bit reddish. **Alive**, the surfaces described as dark brownish are reddish, almost dark orange (face, mandibles, posterior margin of pronotum, ventral surface of fore femora, tibial spines, tarsi, some areas of sternum, abdominal tergites, abdominal spiracles, male cerci, dorsal and ventral margin of ovipositor). Eyes frontally pinkish and dorsally whitish; labrum lighter pinkish than after death (Fig. S2G–L).

#### Type series

**Holotype male** (CELC) \Brasil, MG, Alto Caparaó, P.N. Caparaó. 23–26, XI, 2011. J. Chamorro *leg*. [handwritten on white paper] \1/Listro/Caparao [printed on white paper]\ *Cerberodon portokalipes* Fialho, Chamorro-Rengifo & Lopes-Andrade [handwritten on red paper]\. **Allotype female** (CELC) \Brasil, MG, Alto Caparaó, P.N. Caparaó. 4–8, II, 2012. V. Fialho. [handwritten on white paper] \*Cerberodon portokalipes* Fialho, Chamorro-Rengifo & Lopes-Andrade [handwritten on blue paper]\.

#### Distribution

The specimens examined were sampled in PARNA do Caparaó, locality 11 (Figs. 1B, 22B).

#### Comments

Deimatic behavior was performed by the male holotype and is shown in Fig. S2J. Similar behavior has been observed in species of the listroscelidine *Neobarrettia* Rehn, and species of different subfamilies as in the conocephalines *Mygalopsis ferruginea* Redtenbacher and *Panacanthus pallicornis* (Walker) and the pseudophyline *Acanthodis curvidens* (Stål) ([47]–[49], J. Chamorro-Rengifo pers. obs., with images available in OSF).

***Listroscelis*** **Serville, 1831**

(Figures 11–18, 22C–D, S3)

#### Type species

*Listroscelis armata* Serville, 1831

#### Redescription

**Head.** Eyes globose and protruding, not touching the antennal sockets. Apex of antennal sockets in dorsal view shorter than half the length of eyes. Head elongated, width in dorsal view about twice as long as wide; vertex barely visible in frontal view, not protruding. Frons subtriangular or oval, with a rounded projection at middle, where the defined or undefined ocellus is located. Face with surface smooth or slightly wrinkled. Mandibles with apex elongated and curved inwards, with a basal process at the cutting edge, symmetric or asymmetric in males; when asymmetric, the left mandible has its lateral portion elongated and curved inward (different from *Cerberodon* species, in which the apex of the left mandible is elongated and is devoid of lateral projection). **Thorax.** Pronotum with anterior and posterior margins straight or slightly concave. Prozona with a transverse furrow extending to the lateral lobes, not reaching the lower margin. Mesozona with a transverse furrow, extending to the lateral lobes, sometimes reaching the lower margin. Metazona with a straight transverse furrow extending laterally to the lateral lobes, almost reaching the posterior margin. Lateral lobes with lower margin almost straight; posterior margin slightly oblique, with rounded corners and without sinus humeralis. Each sternite with two spines with rounded tips; prosternal spines comparatively slender; metasternal spines flattened or slender (Fig. 2C). **Wings.** Wings fully developed. Tegmina with a bright yellowish spot at the base. **Legs.** Hind coxae with ventral margin usually bearing one basal and one distal spine, the tip of the spines can be acute or rounded (Fig. 2C). Legs slender. Hind femora as long as the body or slightly longer. Fore and mid femora with a wide longitudinal furrow along the ventral surface. All femora armed with long stout spines interspersed or not with tiny spines. Fore tibiae with surface close to spines blackish, and a small rounded pit dorsally, below and close to each tympanal opening. Mid tibiae with four to six ventral spines. Hind tibiae with several spines on the dorsal and ventral margins. Genicular lobes of all legs ending in an acute tip. Tympanal openings located at the dorsal surface of the tibiae, the width of each opening about one-sixth the length; ear region conspicuously inflated. **Male postabdomen.** Tergite X unmodified or modified, elongated or bearing lobes, emarginated or not; when elongated, it covers epiproct and paraprocts (Fig. 4D, G, J). Cerci with bent apex, or slightly curved inward or downward (Fig. 4D–J); tubercles and bristles covering the entire surface. Epiproct triangular or rounded. Subgenital plate with emargination varying from deep to shallow (Fig. 5G, I, J, L, N, O, Q). Paraprocts triangular and concealing the phallus. Phallus with an entire titillator composed by a transversal bridge which arises from the basal portion of the dorsal lobe, and with two outward projections with rounded tips (Fig. 3E–F). **Female postabdomen.** Subgenital plate sometimes emarginated (Fig. 5H, K, M, P, R, S). Ovipositor slightly upcurved; upper valve about three times as wide as lower valve; apex of ovipositor acute (Figs. 11D, 13D, 14D, 16D, 17D, 18A).

#### Included species

This genus includes *L. angustifrons* (Piza, 1960) **comb. nov.**, *L. armata* Serville, 1831, *L. atrata* Redtenbacher, 1891, *L. carinata* Karny, 1907, *L. ferruginea* Redtenbacher, 1891, *L. magnomaculata* **sp. nov.**, *L. sooretama* **sp. nov.**, *L. cohni* **sp. nov.**, *L. fusca* **sp. nov.**, *L. monnei* **sp. nov.** and *L. itatiaia* **sp. nov**.

***Listroscelis carinata*** **Karny, 1907**

(Figures 11, 22C, S3A–C)

#### Diagnosis

This species can be distinguished from the other *Listroscelis* species by the following combination of features: (i) surface of body dark brownish and greenish, (ii) face, genae and clypeus slightly wrinkled (but not brain-like), (iii) left mandible of male slightly elongated inward, (iv) each tegmen with a yellowish spot of about one-eighth the tegmen length, (v) male tergite X elongated, its width decreasing abruptly from middle to apex, which has a deep median emargination.

**Redescription** (based on images of type material and examination of additional specimens). It is unknown how many specimens were measured by Karny [50], males (n = 2 syntypes): TL 22.00–24.00; PL 6.00; TegL 20.00; HF 20.00–21.00. Females (n = 4 syntypes, type material): TL 23.00–28.00; PL 5.90–6.20; TegL 17.50–21.00; HF 21.00–22.00; OL 16.00–23.00. **Head.** Frons oval and protruding, with well-defined ocellus. Head in frontal view apparently elongated because of the enlarged labrum. Mandibles with apical portion elongated and acuminate, in male the left mandible being more elongated than the right one (Fig. 11C, F). **Wings.** Tegmina with stridulatory file as shown in Fig. 6D. **Male postabdomen.** Cerci of male elongated, forceps-shaped (Fig. 4D), with tip curved downward. Subgenital plate with an emargination leading to a V-shaped cut that is less than half the length of the plate (Fig. 5G). Styli about one-third the length of the plate. **Female postabdomen.** Subgenital plate as shown in Fig. 5H. Ovipositor as long as abdomen (Fig. 11D). **Coloration.** Body mostly dark brownish and dark greenish. Dorsal surface of head dark brownish. Fastigium with dorsal portion yellowish and sides brownish. Eyes dark brownish; dorsal inner surface with a dark yellowish oval area. Sclerites of antennal sockets blackish; antennal scape and pedicel brownish; flagellum dark brownish. Frons dark brownish. Face and clypeus dark brownish. Labrum with basal portion light yellowish and remaining surface dark brownish. Mandibles, maxillary and labial palpi dark brownish. Pronotum dark brownish; lateral lobes without distinct marks. Epimeron with the surface close to the prothoracic spiracle light brownish. Tegmina dark brownish, with primary and secondary veins as well as surface between them brownish. Hind wings light brownish. Legs with coloration pattern as follows: coxae and trochanters dark brownish, femora dark greenish, tibiae dark brownish. Additionally, fore femora have a dark brownish stripe at the lower margin of the lateral portion; in females this stripe is weaker. Femoral and tibial spines dark brownish. Surface surrounding tympana dark brownish. Tarsi dark brownish, the third-one darker; claws dark brownish with darker tips. **Alive**, some parts are aquamarine blue, as follows: ventral portion of legs, pleura close to all coxae, abdominal sternites, pleurites, subgenital plate and cerci. Parts which are light greenish: dorsal and lateral area of legs, coxae, trochanters, and basal portion of the ovipositor. Femora with a whitish spot at the base of each spine. Abdominal spiracles whitish. Tergites somewhat light purple (Fig. S3A–C).

#### Variation

Type material data not included. Measurements of male (n = 1): TL 23.00; PL 8.00; PW 5.00; EyeW 2.50; TegL 21.00; SL 2.82; NT 158; minT 0.03; maxT 0.08; HF 21.00; HT 25.00; sFF, inner margin, large 3, small 0–6, outer large 3, small 4–0; sMF, inner margin, large 4, small 20–17, outer large 5, small 24–18; sHF, inner margin, large 11–12, small 5, outer large 10, small 7–9; sMTld 5; sHTd, inner margin 16, outer 16–18; sHTv, inner margin 13–16, outer 13–15. Measurements of females (n = 6): TL 24.00–31.00; PL 6.00–7.00; PW 4.00–5.00; EyeW 2.00–3.00; TegL 18.00–20.00; HF 22.00–33.00; HT 23.00–25.00; sFF, inner margin, large 3–4, small 10–14, outer large 4, small 15–17; sMF, inner margin, large 4, small 13–21, outer large 4–5, small 19–21; sHF, inner margin, large 10–12, small 0–7, outer large 9–12, small 3–5; sMTld 5; sHTd, inner margin 16–17, outer 16–17, sHTv, inner margin 13–15, outer 10–14; OL 18.00–22.00.

#### Specimens examined

One adult male (MNRJ) \Collatina. E. do. E. Santo. M. Rosa, Out. 36 [printed on yellowish paper] \ No. Proc. 58/512 [type- and handwritten on yellow paper] \ *Listroscelis carinata* Karny, 1907 [handwritten on white paper]\. One adult female (MNRJ) \Collatina. E. do. Santo. M. Rosa, Out. 36 [printed on yellowish paper] \ No. Proc. 58/510 [type- and handwritten on yellow paper] \ *Listroscelis carinata* Karny, 1907 [handwritten on white paper]\; one adult female (MNRJ) \Collatina. E. do. Santo. M. Rosa, Out. 36 [printed on yellowish paper] \ No. Proc. 58/511 [type- and handwritten on yellow paper] \ *Listroscelis carinata* Karny, 1907 [handwritten on white paper]\. Four adult females and two immature females (CELC) \Brasil, MG, P.E. Rio Doce, J. Chamorro *leg*. [handwritten on white paper] \ with the respective codes \1\, \2\, \4\, \7\, \5\ and \6/Listro/Riodoce [printed on white paper] \ *Listroscelis carinata* Karny, 1907 [handwritten on white paper]\.

#### Distribution

The specimens examined were sampled in Parque Estadual (P.E.) do Rio Doce, locality 7 (Figs. 1B, 22C) or borrowed from MNRJ. The accurate type locality of *L. carinata* is unknown. The only available information is: “Minas Gerais, Espirito Santo”.

#### Comments

Examined specimens from MNRJ have altered coloration, with dark brownish face and abdominal sternites of males. *Listroscelis carinata* is most similar to *L. itatiaia* **sp. nov**.

***Listroscelis angustifrons*** **(Piza, 1960) comb. nov.**

*Cerberodon angustifrons* Piza, 1960

(Figures 12, 22C)

The species is here transferred to *Listroscelis*. It cannot remain in *Cerberodon* because species of that genus have strongly wrinkled face, genae and basal portion of the clypeus, with a conspicuous brain-like appearance (Figs. 9C, F, 10C, F), while in *L. angustifrons* **comb. nov.** they are smooth or slightly wrinkled (Fig. 12B). Each tegmen of *Listroscelis angustifrons* **comb. nov.** has a bright spot on the basal portion (Fig. 12A), similar to other *Listroscelis* species (e.g. Figs. 11A, D, 13A, D, 16A, D), while species of *Cerberodon* are devoid of such spots at tegmina (Figs. 9A, D, Figs. 10A, D). In *L. angustifrons* **comb. nov.** the titillator is a single, undivided sclerite, a feature common to all examined *Listroscelis* species (Fig. 3E), while in *Cerberodon* the titillator has two separated sclerites (Fig. 3C).

#### Diagnosis

This species can be distinguished from the other *Listroscelis* species by the following combination of features: (i) surface of body mostly chestnut-colored and blackish, (ii) surface of face, genae and clypeus slightly wrinkled, not brain-like, (iii) left mandible of male elongated and held beyond the frontal plane of the head, tip upcurved, (iv) each tegmen with a yellowish spot of about one-tenth its length, (v) male tergite X unmodified.

#### Redescription (holotype ♂)

Measurements of the holotype ♂: TL 29.00; PL 8.00; TegL 25.00; FF 20; HF 26.00. **Head.** Frons triangular and protruding, with a well-developed ocellus (Fig. 12B). **Wings.** Tegmina with stridulatory file as shown in Fig. 6E. In some specimens the coxae bear a ventral spine with acute or rounded tip, on their basal and distal portions. **Male postabdomen.** Cerci triangular, with tip curved inward (Fig. 4E). Subgenital plate with a U-shaped emargination of about one-sixth its length (Fig. 5I). Styli long, of about four-fifths the length of the plate. **Coloration.** Body mostly chestnut-colored and blackish. Dorsal surface of head chestnut-colored. Fastigium with a yellowish longitudinal dorsal stripe. Eyes blackish with a yellowish oval area on their dorsal inner surface. Sclerites of antennal sockets blackish; antennal scape and pedicel frontally blackish; frontal and dorsal surface with diffuse yellowish and blackish marks; flagellum blackish. Frons with ocellus yellowish. Face chestnut-colored. Mouthparts in ventral view blackish, but palpi yellowish. Clypeus with yellowish lateral stripes and median stripe. Labrum with basal portion yellowish, the remaining surface chestnut-colored. Mandibles chestnut-colored. Maxillary and labial palpi light brownish. Pronotum chestnut-colored, lateral lobes without distinctive marks. Epimeron with surface close to upper and anterior margins of the prothoracic spiracle blackish, and lower and posterior margins dark brownish. Tegmina dark brownish with primary and secondary veins as well as surface between them light brownish. Hind wings light brownish. Legs with the following coloration pattern: coxae and trochanters chestnut-colored; femora ventrally blackish. Tibiae light brownish. Femoral spines dark brownish. Additionally, the spines on the fore and mid legs have a yellowish oval spot at their inner base. Tibial spines light brownish. Ear region darker. Tarsi light brownish, the third-one darker; claws dark brownish, the tip darker. Sternites chestnut-colored. Abdominal tergites dark brownish and sternites blackish.

#### Variation

Measurements of males (n = 3, including the holotype): TL 25.00–26.00; PL 7.00–8.00; PW 5.00; EyeW 3.00; TegL 21.00–25.00; HF 24.00–26.00; HT 26.00; sFF, inner margin, large 4, small 9–13, outer large 4–6, small 17–26; sMF, inner margin, large 4, small 19–13, outer large 5–5, small 13–20; sHF, inner margin, large 10–12, small 2–5, outer large 9–13, small 0–13; sMTld 0–6; sHTd, inner margin 16–17, outer 17; sHTv, inner margin 15–15, outer 13–13. (n = 1): SL 1.68; NT 62; minT 0.02; maxT 0.011.

#### Specimens examined

**Holotype male** (ESALQ) labeled as follow: \Espirito Santo, E Garbe leg. [type- and handwritten on yellow paper]\*Listroscelis angustifrons* (Piza, 1960) [printed on white paper]\*Cerberodon angustifrons* Piza, tipo [handwritten on yellow paper]\MZLQ-I 0062, E.S.A. Luiz de Queiroz – U.S.P., ZOOLOGIA, Piracicaba – S.P. Brasil [type- and handwritten on yellow paper] \ 92.752 [typewritten on white paper]\. One adult male (UFES), specimen labeled as follow: \Brasil: ES, D. Martins. Zona rural. 12. XXX.1999 - manual. M.V. Amado col. [handwritten on white paper] \ *Listroscelis angustifrons* (Piza, 1960) [printed on white paper]\. One adult male (in alcohol) (CELC), specimen labeled as follow: \Alagados do Itabapoana – Mata. Presidente Kennedy – ES. Fevereiro – 2012. Furieri, K. S & Loiola, G. R [handwritten on white paper] \ *Listroscelis angustifrons* (Piza, 1960) [printed on white paper]\.

#### Distribution

The type-locality is “Espírito Santo”, but the exact locality is unknown. The examined specimens are registered from Alagados do Itabapoana and Domingos Martins, in Espírito Santo (Fig. 22C). These are the first accurate records of geographic distribution for the species. Two males collected from Espírito Santo (Museum of Vienna) were erroneously identified by Redtenbacher as *L. atrata*. The correct identification is *L. angustifrons* **comb. nov**. The species is most similar to *L. atrata* and *L. magnomaculata* **sp. nov**.

***Listroscelis magnomaculata*** Fialho, Chamorro-Rengifo & Lopes-Andrade **sp. nov.** urn:lsid:zoobank.org:act:B9810483-60EF-4AA2-9518-F5DEE60F90A3

(Figures 13, 22D, S3D–F)

#### Etymology

The specific epithet refers to the large yellowish spot at the base of each tegmen.

#### Diagnosis

This species can be distinguished from the other *Listroscelis* species by the following combination of features: (i) surface of body mostly dark chestnut-colored and light and dark greenish, (ii) face, genae and clypeus with transverse wrinkles arranged horizontally, parallel to the vertex, (iii) left mandible of male with pre-apical portion elongated and bent upward, (iv) base of each tegmen with a yellowish spot of about one-quarter the length of tegmen, (v) male tergite X unmodified.

#### Description (holotype ♂ & allotype ♀)

Holotype ♂: TL 25.00; PL 7.00; PW 5.00; EyeW 3.00; TegL 14.00; SL 2.11; NT 73; minT 0.02; maxT 0.09; HF 23.00; HT 26.00; sFF, inner margin, large 4–5, small 24–25, outer large 5, small 30–32; sMF, inner margin, large 5, small 23–26, outer large 4, small 28; sHF, inner margin, large 11–12, small 21, outer large 12–13, small 12–19; sMTld 4; sHTd, inner margin 16–17, outer 15; sHTv, inner margin 10–13, outer 10–13. Allotype ♀: TL 28.00; PL 6.00; PW 5.00; EyeW 3.00; TegL 14.00; HF 23.00; HT 26.00; sFF, inner margin, large 4, small 11–14, outer large 5–6, small 25–26; sMF, inner margin, large 5–6, small 29–30, outer large 4–5, small 22–23; sHF, inner margin, large 7–15, small 5–12, outer large 11, small 4–10; sMTld 4; sHTd, inner margin 18, outer 15–16; sHTv, inner margin 9–10, outer 10–11; OL 21.00. **Head.** Frons oval and protruding, with a well-defined ocellus. Face and basal portion of the clypeus with transversal wrinkles arranged parallel to vertex (Fig. 13C, F). **Wings.** Tegmina with stridulatory file as shown in Fig. 6F. **Male postabdomen**. Cerci stout and elongated, tapering from base to apex; tip curved inward (Fig. 4F). Subgenital plate (Fig. 5J) with a U-shaped emargination of about one-quarter its length in the midline. Styli about one-third the length of the plate. **Female postabdomen.** Subgenital plate as shown in Fig. 5K. Ovipositor slightly longer than abdomen (Fig. 13D). **Coloration.** Body mostly chestnut-colored and greenish. Dorsal surface of head chestnut-colored. Vertex dorsally dark yellowish and laterally blackish. Sclerites of antennal sockets blackish; antennal scape and pedicel blackish with indistinct dark brownish spots; flagellum blackish. Eyes dark yellowish with diffuse dark brownish spots, and a dark yellowish oval area at the inner dorsal surface. Frons dark brownish. Face and basal portion of clypeus chestnut-colored, disc and apical portion of clypeus yellowish, with diffuse dark brownish spots. Labrum somewhat blackish, with basal portion dark brownish. Mandibles blackish. Ventral portion of mouthparts dark brownish. Maxillary and labial palpi light greenish, with blackish spots at the apical portion of each palpus. Pronotum chestnut-colored, lateral lobes without distinctive marks. Epimeron close to the prothoracic spiracle fluorescent yellowish. Tegmina with portion between costal region and R vein, and between Sc vein and the lower margin, greenish fluorescent and blackish; region between R and Sc veins brownish. Primary veins brownish, secondary veins greenish, surface between veins blackish. Hind wings dark brownish. Sternum blackish; spines and furrows light yellowish. Legs with ventral portion of coxae blackish; mid and hind coxae with undefined dark yellowish spots, and lateral and ventral portions light brownish. Trochanters with color similar to that of coxae. Femora and tibiae of all legs with coloration pattern as follows: dorsal portion light brownish, ventral portion blackish and apical portion of ventral edge light brownish. Large spines of each femur with a whitish oval spot at the inner base. Tibiae light greenish with surface close to the spines light brownish; spines dark brownish. Hind femora with lateral portion bearing a brownish stripe parallel to the ventral margin. Surface surrounding tympana light brownish. Tarsi with first, second and fourth tarsomeres light brownish; apical portion of each tarsus dark brownish; third tarsomere blackish, with undefined dark brownish spots; claws dark brownish. Abdominal tergites with disc light brownish, and sides dark brownish. Abdominal sternites blackish. Male subgenital plate with basal portion dark brownish and the remaining light brownish. **Alive**, specimens with coloration similar to that described above (Fig. S3D–F).

#### Variation

Measurements of males (n = 8, including the holotype): TL 23.00–26.00; PL 7.00; PW 5.00–6.00; EyeW 2.00–3.00; TegL 13.00–14.00; HF 22.00–24.00; HT 24.00–26.00; sFF, inner margin, large 4–5, small 27–35, outer large 5, small 28–37; sMF, inner margin, large 4, small 29–33, outer large 3–4, small 24–35; sHF, inner margin, large 4–15, small 21–25, outer large 10–13, small 9–22; sMTld 4; sHTd, inner margin 16–18, outer 15–17; sHTv, inner margin 10–17, outer 9–13. Stridulatory file (n = 2) SL: 2.11–2.23; NT: 73–78; minT 0.02–0.03. Measurements of females (n = 2, including the allotype): TL 27.00–28.00; PL 6.00–7.00; PW 5.00; EyeW 3.00; TegL 12.00–14.00; HF 22.00–23.00; HT 25.00–26.00; sFF, inner margin, large 4–5, small 10–14, outer large 5–6, small 25–26; sMF, inner margin, large 5–6, small 14–30, outer large 4–6, small 22–24; sHF, inner margin, large 7–15, small 1–12, outer large 9–11, small 0–10; sMTld 4–4; sHTd, inner margin 16–18, outer 15–16; sHTv, inner margin 9–12, outer 9–11; OL 20.00–21.00.

#### Type series

All specimens collected at single locality and labeled as follows: \Brasil, Camacan, BA, RPPN Serra Bonita, 10–12, I, 2012, J. Chamorro *leg*. [handwritten on white paper]\. **Holotype male** (CELC) additionally labeled \9–Listro–Bonita [printed on white paper] \ *Listroscelis magnomaculata* Fialho, Chamorro-Rengifo & Lopes-Andrade 2014 [handwritten on red paper]\. **Allotype female** (CELC) additionally labeled \7/Listro/Bonita \ *Listroscelis magnomaculata* Fialho, Chamorro-Rengifo & Lopes-Andrade 2014 [handwritten on blue paper]\. **Paratypes** (CELC) additionally labeled \*Listroscelis magnomaculata* Fialho, Chamorro-Rengifo & Lopes-Andrade 2014 [handwritten on blue paper]\. Seven adult males with the respective codes \1\, \2\, \3\, \4\, \5\, \13\, \14\; one immature male \26\; one adult female \15\ and additionally labeled \Listro/Bonita\.

#### Distribution

The specimens were sampled in RPPN Serra Bonita, locality 2 (Figs. 1B, 22D). Individuals of this species were abundant in the field, unlike other species of the genus.

***Listroscelis sooretama*** Fialho, Chamorro-Rengifo & Lopes-Andrade **sp. nov.** urn:lsid:zoobank.org:act:32500099-AA13-403C-B149-1DB783B7DA20

(Figures 14, 22D, S3G–H)

#### Etymology

The specific epithet refers to “Sooretama”, a word of the Tupi indigenous language that means “land” and “refuge of animals of the forest”. It comes from the word “soo” or “çoó” (animal, hunt) and “retama” (place, native land, homeland). It is also the name of the type-locality and the conservation unit where the species was collected.

#### Diagnosis

This species can be distinguished from the other *Listroscelis* by the following combination of features: (i) surface of body light greenish with few light brownish areas, (ii) face, genae and clypeus smooth, (iii) mandibles symmetric, (iv) each tegmen with yellowish spot about one-twelfth its length, (v) male tergite X elongated, its width decreasing abruptly from middle to apex; posterior margin with a deep oval-shaped emargination at middle.

#### Description (holotype ♂ & allotype ♀)

Holotype ♂: TL 26.00; PL 7.00; PW 5.00; EyeW 3.00; TegL 22.00; SL 2.22; NT 136; minT 0.01; maxT 0.06; HF 23.00; HT 25.00; sFF, inner margin, large 4–5, small 23–27, outer large 5, small 36; sMF, inner margin, large 5, small 32, outer large 4, small 27; sHF, inner margin, large 13–15, small 18–21, outer large 12–13, small 7–10; sMTld 5; sHTd, inner margin 16–18, outer 15; sHTv, inner margin 10, outer 12–13. Allotype ♀: TL 27.00; PL 6.00; PW 5.00; EyeW 3.00; TegL 22.00; HF 12.00; HT 28.00 sFF, inner margin, large 4–5, small 19–21, outer large 4–5, small 26–28; sMF, inner margin, large 5, small 31, outer large 4, small 30; sHF, inner margin, large 13–15, small 12–13, outer large 12, small 7–10; sMTld 6; sHTd, inner margin 18, outer 16–17; sHTv, inner margin 10–11, outer 13; OL 26.00. **Head.** Frons triangular and protruding, ocellus weakly defined (Fig. 14C, F). Face somewhat smooth, barely wrinkled. **Wings.** Tegmina with stridulatory file as shown in Fig. 6G. **Male postabdomen**. Cerci with apical portion slightly curved downwards (Fig. 4G). Subgenital plate wide, its posterior margin with a V-shaped emargination at middle (Fig. 5L). Styli of about one-fifth the length of the plate. **Female postabdomen.** Subgenital plate as shown in Fig. 5M. Ovipositor a bit longer than abdomen (Fig. 14D). **Coloration.** Body mostly light greenish and light brownish. Dorsal surface of head light greenish. Fastigium dorsally whitish and laterally brownish. Sclerites of antennal sockets blackish; antennal scape and pedicel blackish; flagellum dark brownish. Eyes dark brownish with diffuse blackish spots. Face light brownish; sides light greenish. Clypeus and labrum light brownish. Mandibles with basal portion and sides light brownish; remaining surface, including cutting edge, blackish. Ventral portion of mouthparts light brownish. Maxillary and labial palpi greenish. Pronotum light greenish, lateral lobes without distinctive marks. Sternites and spines light greenish. Tegmina, including primary and secondary veins, light greenish; surface between veins dark brownish. Legs, including coxae and trochanters, light greenish; spines of femora and tibiae light brownish. Ear region light brownish. Tarsi light greenish, the claws light greenish with dark brownish tips. Abdominal tergites and sternites light greenish. **Alive**, individuals had more vivid greenish coloration (Fig. S3G–H).

#### Type series

**Holotype male** (CELC) \Brasil, ES, Linhares, ReBio de Sooretama. 29–XI – 2–XII, 2011. J. Chamorro *leg.* [handwritten on white paper] \ 6–Listro–Sooretama [printed on white paper] \ *Listroscelis sooretama* Fialho, Chamorro-Rengifo & Lopes-Andrade 2014 [handwritten on red paper]\. **Allotype female** (CELC), same locality label as holotype and additionally labeled \1–Listro–Sooretama [printed on white paper]\ *Listroscelis sooretama* Fialho, Chamorro-Rengifo & Lopes-Andrade 2014 [handwritten on blue paper]\.

#### Distribution

The specimens were sampled in Reserva Biológica (ReBio) de Sooretama, locality 9 (Figs. 1B, 22D). We have also seen images from a specimen deposited in the Museum of Vienna, collected at “Reserva do Rio Doce” in Espírito Santo, near the type-locality.

***Listroscelis cohni*** Fialho, Chamorro-Rengifo & Lopes-Andrade **sp. nov.** urn:lsid:zoobank.org:act:D46ADB06-E50E-4F93-B98E-CE52B537648F

(Figures 15, 22C)

#### Etymology

The specific epithet is in honor after Theodore Cohn, katydid specialist who recently passed away. He had a special interest in Listroscelidinae and studied *Neobarretia*, the only genus which represents this subfamily in North America.

#### Diagnosis

This species can be distinguished from the other *Listroscelis* species by the following combination of features: (i) body mostly dark to light brownish and light greenish, (ii) face, genae and clypeus almost smooth, slightly wrinkled, (iii) mandibles symmetric, (iv) each tegmen with a yellowish spot of about one-sixteenth its length, (v) male tergite X unmodified.

#### Description (holotype ♂)

Holotype ♂: TL 26.00; PL 6.00; PW 5.00; EyeW 3.00; TegL 22.00; SL 2.68; NT 178; minT 0.02; maxT 0.08; HF 22.00; HT 24.00; sFF, inner margin, large 5–6, small 27, outer large 5, small 35–38; sMF, inner margin, large 5, small 29, outer large 5, small 28; sHF, inner margin, large 10–12, small 22–25, outer large 16–17, small 12–22; sMTld 4; sHTd, inner margin 18, outer 16–18; sHTv, inner margin 9–12, outer 11. **Head.** Frons triangular, with well-defined ocellus (Fig. 15C). **Wings.** Tegmina with stridulatory file as shown in Fig. 6H. **Male postabdomen**. Cerci with apical portion bend inward (Fig. 4H). Subgenital plate with a deep V-shaped emargination (Fig. 5N); styli one-third the length of the plate. **Coloration.** Body surface mostly dark to light brownish and light greenish. Dorsal surface of head dark brownish. Fastigium light yellowish dorsally and dark brownish laterally. Sclerites of antennal sockets dark brownish. Antennal scape and pedicel light brownish; flagellum dark brownish. Frons dark brownish. Eyes reddish. Face dark brownish. Mouthparts in ventral view whitish. Maxillary and labial palpi light greenish. Pronotum dark brownish, lateral lobes without distinctive marks. Prothoracic spiracle with the upper and lateral margin dark brownish. Sternum yellowish with spines light greenish. Tegmina with primary and secondary veins light greenish; surface between veins dark brownish; stridulatory area darker. Hind wings light greenish with primary veins dark brownish. Fore legs, including coxae and trochanters light brownish. Femora light brownish, lower margin lighter. Femoral spines and tibiae dark brownish with darker tips. Tibiae with apical portion dark brownish and remaining surface light greenish; surface close to spines blackish. Ear region blackish. Mid and hind coxae and trochanters yellowish. Mid legs light greenish. Hind femora and hind tibiae light greenish; apical and basal portions dark brownish. Spines of mid and hind legs light brownish. Tarsi light brownish, the third one darker than the others; claws light brownish with darker tips. Abdominal tergites light brownish; sternites dark brownish.

#### Type series

**Holotype male** (CELC) \Brasil, BA, P.N. do Descobrimento. J. Chamorro *leg*. 13–15, I, 2012 [handwritten on white paper] \ 4/Listro/Descobrimento [printed on white paper] \ *Listroscelis cohni* Fialho, Chamorro-Rengifo & Lopes-Andrade 2014 [handwritten on red paper]\.

#### Distribution

The specimen was sampled in PARNA do Descobrimento, locality 4 (Figs. 1B, 22C).

***Listroscelis fusca*** Fialho, Chamorro-Rengifo & Lopes-Andrade **sp. nov.** urn:lsid:zoobank.org:act:D3B42C42-A832-4260-A70B-0C6AABB8CD13

(Figures 16, 22D, S3I–J)

#### Etymology

The specific epithet refers to the brownish body surface of specimens.

#### Diagnosis

This species can be distinguished from the other *Listroscelis* species by the following combination of features: (i) surface of body dark and light brownish, (ii) face, genae and clypeus smooth in females and slightly wrinkled in males, (iii) left mandible of male bent before apex and with tip projecting upward, (iv) each tegmen with a yellowish spot of about one-eleventh its length, (v) male tergite X unmodified.

#### Description (holotype ♂ & allotype ♀)

Holotype ♂: TL 28.00; PL 7.00; PW 5.00; EyeW 3.00; TegL 20.00; SL 2.20; NT 92; minT 0.04; maxT 0.11, HF 20.00; HT 21.00; sFF, inner margin, large 4–5, small 15–18, outer large 5, small 17; sMF, inner margin, large 5, small 22, outer large 5, small 21; sHF, inner margin, large 13, small 0–3, outer large 12, small 0; sMTld 5; sHTd, inner margin 16, outer 14–15; sHTv, inner margin 10, outer 11–13. Allotype ♀: TL 24.00; PL 6.00; PW 4.00; EyeW 3.00; TegL 20.00; HF 19.00; HT 21.00; sFF, inner margin, large 4–5, small 14–16, outer large 4–5, small 20–27; sMF, inner margin, large 4, small 20, outer large 4–5, small 17; sHF, inner margin, large 0–13, small 0, outer large 0, small 0; sMTld 4; sHTd, inner margin 16, outer 14; sHTv, inner margin 7–9, outer 10; OL 16.00. **Head.** Frons triangular and protruding, with a weakly defined ocellus (Fig. 16C, F). Face slightly wrinkled in male and smooth in female. **Wings.** Tegmina with stridulatory file as shown in Fig. 6I. **Male postabdomen.** Cerci stout, with tip strongly curved inwards (Fig. 4I). Subgenital plate with a V-shaped emargination at midline (Fig. 5O); styli of about half the length of the plate. **Female postabdomen.** Subgenital plate as shown in Fig. 5P. Ovipositor slightly longer than abdomen (Fig. 16D). **Coloration.** Surface of body mostly dark and light brownish. Dorsal surface of head dark brownish, with a whitish stripe extending from the tip of the fastigium to the posterior margin of head. Fastigium with lateral portions dark brownish. Eyes dark brownish with diffuse darker spots. Sclerites of antennal sockets blackish. Antennal scape and pedicel in male somewhat blackish; pedicel with the inner area yellowish, in female dark brownish with diffuse blackish spots. Flagellum light brownish. Frons and face dark brownish. Clypeus in male whitish with yellowish margin, in female with basal portion close to the angles blackish; labrum blackish with basal portion yellowish. Mandibles blackish. Ventral surface of mouthparts yellowish. Maxillary and labial palpi light greenish. Pronotum dark brownish; each lateral lobe with a blackish stripe from the lower to the posterior margin. Epimeron with surface close to the prothoracic spiracle light brownish; prothoracic spiracle with blackish margins. Thoracic sternites and spines yellowish. Tegmina light brownish with primary veins light brownish, secondary veins light greenish, surface between veins light brownish. Legs with coxae and trochanters yellowish and with diffuse dark brownish spots; mid and hind coxae and trochanters darker than those of fore legs. Femora and tibiae dark brownish. Apical portion of femora darker. Femoral spines dark brownish. Hind femora with lower margin blackish. Tibial spines light brownish. Tarsi dark brownish; claws dark brownish with blackish tips. Abdominal tergites light brownish and sternites dark brownish. **Alive**, specimens with abdominal tergites and sternites light greenish; whitish stripe at the dorsal surface of head more vivid (Fig. S3I–J).

#### Type series

**Holotype male** (CELC) \Brasil, MG, Araponga, P.E. Brigadeiro. 12–15, XII, 2011. J. Chamorro *leg.* [handwritten on white paper] \ 2–Listro–Brigadeiro [printed on white paper] \ *Listroscelis fusca* Fialho, Chamorro-Rengifo & Lopes-Andrade 2014 [handwritten on red paper]\. **Allotype female** (CELC), same locality and data as the holotype, and additionally labeled \1–Listro–Brigadeiro [printed on white paper] \ *Listroscelis fusca* Fialho, Chamorro-Rengifo & Lopes-Andrade 2014 [handwritten on blue paper]\.

#### Distribution

The specimens were sampled in P.E. Serra do Brigadeiro, locality 10 (Figs. 1B, 22D).

***Listroscelis monnei*** Fialho, Chamorro-Rengifo & Lopes-Andrade **sp. nov.** urn:lsid:zoobank.org:act:DE59EBB6-1FD9-48B6-8B85-60A9716E4145

(Figures 17, 22D, S3K)

#### Etymology

The specific epithet is dedicated to Miguel Angelo Monné, great taxonomist of Neotropical Cerambycidae (Coleoptera), curator of the entomological collection and Emeritus Research at the Museu Nacional do Rio de Janeiro, Brazil.

#### Diagnosis

This species can be distinguished from the other *Listroscelis* species by the following combination of features: (i) body mostly dark and light brownish, (ii) face, genae and clypeus mostly smooth, with few wrinkles, (iii) mandibles symmetric, (iv) each tegmen with a yellowish spot of about one-tenth its length, (v) male tergite X wide, with an oval-shaped emargination of about one-quarter the length of the plate at middle.

#### Description (holotype ♂ & allotype ♀)

Holotype ♂: TL 32.00; PL 8.00; PW 6.00; EyeW 4.00; TegL 29.00; SL 3.86; NT 153; minT 0.04; maxT 0.16; HF 25.00; HT 26.00; sFF, inner margin, large 4–5, small 24–27, outer large 6, small 30–39; sMF, inner margin, large 5, small 37, outer large 5, small 27; sHF, inner margin, large 14, small 1–4, outer large 14, small 2–4; sMTld 0; sHTd, inner margin 16–19, outer 15–17; sHTv, inner margin 11–14, outer 11–13. Allotype ♀: TL 36.00; PL 8.00; PW 6.00; EyeW 3.00; TegL 29.00; HF 27.00; HT 28.00; sFF, inner margin, large 4–5, small 21–22, outer large 5, small 31–32; sMF, inner margin, large 4, small 32, outer large 4, small 27; sHF, inner margin, large 14, small 10–14, outer large 13–14, small 4–13; sMTld 3; sHTd, inner margin 16–17, outer 15–16; sHTv, inner margin 10–11, outer 13–14; OL 25.00. **Head**. Frons triangular with a well-defined ocellus (Fig. 17C, F). Face mostly smooth, with few wrinkles. **Wings.** Tegmina with stridulatory file as shown in Fig. 6J. **Male postabdomen**. Cerci stout, apical portion narrowed and curved downwards (Fig. 4J). Subgenital plate wide, with a V-shaped emargination (Fig. 5Q). Styli of about one-fifth the length of the plate. **Female postabdomen**. Subgenital plate as shown in Fig. 5R. Ovipositor a bit longer than abdomen (Fig. 17D). **Coloration.** Body mostly light and dark brownish. Dorsal surface of head dark brownish. Fastigium yellowish dorsally and light brownish laterally. Eyes dark brownish with diffuse darker spots. Sclerites of antennal sockets blackish; antennal scape and pedicel light brownish with indistinct dark brownish spots; flagellum dark brownish. Clypeus yellowish, labrum light brownish with dark, almost black apical portion. Mandibles blackish. Ventral surface of mouthparts light brownish. Maxillary and labial palpi yellowish. Pronotum dark brownish; lateral lobes without distinctive marks. Sternum and spines light brownish. Epimeron close to the prothoracic spiracle light brownish; prothoracic spiracle with blackish margin. Tegmina light brownish; primary veins dark brownish, secondary veins light brownish, surface between veins dark brownish. Hind wings light brownish. Legs, including coxae and trochanters, light brownish; femora darker than tibiae; femoral spines dark brownish; tibial spines dark brownish with lighter tips. Ear region dark brownish. Tarsi light brownish, the third one darker; claws light brownish with darker tip. Abdominal tergites and sternites light brownish. **Alive**, they have almost the same colors as preserved specimens (Fig. S3K).

#### Variation

Measurements of females (n = 2, including the allotype): TL 36.00–39.00; PL 8.00–9.00; PW 6.00; EyeW 3.00; TegL 29.00–31.00; HF 27.00–30.00; HT 20.00–33.00; sFF, inner margin, large 4–5, small 21–27, outer large 5, small 31–39; sMF, inner margin, large 5–4, small 32–33, outer large 4, small 27–34; sHF, inner margin, large 10–14, small 10–17, outer large 10–14, small 4–15; sMTld 6; sHTd, inner margin 16–18, outer 15–16; sHTv, inner margin 10–11, outer 13–16; OL 25.00–27.00.

#### Type series

Specimens labeled \Brasil, BA, P.N. do Descobrimento. J. Chamorro *leg*. 13–15, I, 2012 [handwritten on white paper]\. Additionally labeled: **Holotype male** (CELC) \23–Listro–Descobrimento [printed on white paper] \ *Listroscelis monnei* Fialho, Chamorro-Rengifo & Lopes-Andrade 2014 [handwritten on red paper]\. **Allotype female** (CELC) \1–Listro–Descobrimento [printed on white paper] \ *Listroscelis monnei* Fialho, Chamorro-Rengifo & Lopes-Andrade 2014 [handwritten on blue paper]\. **Paratype females** (CELC), one labeled \2–Listro–Descobrimento [printed on white paper] \ *Listroscelis monnei* Fialho, Chamorro-Rengifo & Lopes-Andrade 2014 [handwritten on blue paper]\, another labeled \ Brasil, Camacan, BA, RPPN Serra Bonita, 10–12, I, 2012, J. Chamorro *leg*. [printed on white paper] \ *Listroscelis monnei* Fialho, Chamorro-Rengifo & Lopes-Andrade 2014 [handwritten on white paper] \ 10/Listro/Bonita [printed on white paper]\.

#### Distribution

The specimens were sampled in PARNA do Descobrimento, locality 4 and RPPN Serra Bonita, locality 2 (Figs. 1B, 22D).

***Listroscelis itatiaia*** Fialho, Chamorro-Rengifo & Lopes-Andrade **sp. nov.** urn:lsid:zoobank.org:act:5429196A-F654-4431-B176-E033812601C3

(Figures 18, 22D, S3L)

#### Etymology

The specific epithet is from the Tupi indigenous language and means “pointy stone”. It comes from the words “itá” (stone) and “atîaî” (pointy). Itatiaia is also the name of the oldest national park of Brazil, where this species was found.

#### Diagnosis

It can be distinguished from the other *Listroscelis* species by the following combination of features: (i) surface of body dark and light brownish and light greenish, (ii) face, genae and clypeus smooth, (iii) each tegmen with a yellowish spot of about one-quarter its length.

#### Description (holotype ♀)

Holotype ♀: TL 25.00; PL 7.00; PW 4.00; EyeW 3.00; TegL 19.00; HF 22.00; HT 23.00; sFF, inner margin, large 4, small 6–8, outer large 4–5, small 8–9; sMF, inner margin, large 4, small 20, outer large 4, small 15; sHF, inner margin, large 14, small 5–7, outer large 11–12, small 0; sMTld 3; sHTd, inner margin 16, outer 16; sHTv, inner margin 10–11, outer 13–14; OL 19.00. **Head**. Frons oval, with undefined ocellus (Fig. 18C). **Female postabdomen**. Subgenital plate as shown in Fig. 5S. Ovipositor slightly shorter than abdomen (Fig. 18A). **Coloration.** Surface of body mostly dark to light brownish and light greenish. Dorsal surface of head dark brownish. Fastigium dorsally whitish and laterally brownish. Eyes dark brownish, slightly reddish, with diffuse blackish spots. Antennal sockets blackish; antennal scape, pedicel and remaining antennomeres dark brownish. Frons dark brownish. Face dark brownish, lighter at sides; below eyes yellowish. Clypeus dark brownish with diffuse blackish spots. Labrum yellowish with basal portion dark brownish. Mandibles blackish. Ventral portion of mouthparts yellowish. Maxillary and labial palpi whitish. Pronotum dark brownish; lateral lobes with a dark stripe at the posterior margins. Sternites and spines light greenish. Tegmina greenish; primary veins light brownish, secondary veins light greenish, surface between veins blackish. Legs with coxae and trochanters light greenish. Fore femora light greenish, with lower margin bearing a black stripe; spines light greenish with blackish tips; tibiae dark brownish. Mid femora light greenish with lower margin whitish; spines dark brownish with inner base whitish; tibiae dark brownish. Hind femora dark brownish, with lower margin light greenish; spines light greenish with dark brownish tips; tibiae light brownish. Tarsi light brownish, the third one darker; claws light brownish with darker tip. Abdominal tergites dark brownish with diffuse reddish marks; sternites light brownish. **Alive**, as shown in Fig. S3L.

#### Variation

Measurements of females (n = 3, including the holotype): TL 22.00–29.00; PL 6.00–7.00; PW 4.00–5.00; EyeW 3.00; TegL 19.00–22.00; HF 22.00; HT 23.00–24.00; sFF, inner margin, large 4, small 6–20, outer large 4–5, small 8–24; sMF, inner margin, large 4–5, small 20–24, outer large 4, small 15–18; sHF, inner margin, large 12–14, small 1–7, outer large 11–13, small 0; sMTld 4; sHTd, inner margin 13–17, outer 14–16; sHTv, inner margin 10–13, outer 10–14; OL 16.00–19.00.

#### Type series

**Holotype female** (CELC) \Brasil, RJ, P.N. Itatiaia. 7–13, XI, 2011 J. Chamorro *leg*. [handwritten on white paper] \ 6/Listro/Itatiaia [printed on white paper] \ *Listroscelis itatiaia* Fialho, Chamorro-Rengifo & Lopes-Andrade 2014 [handwritten on red paper]\. **Two female paratypes** (CELC) labeled as the holotype, with the respective codes \3\,\8\ and additionally labeled \Listro/Itatiaia [printed on white paper]\.

#### Distribution

The specimens were sampled in PARNA do Itatiaia, locality 13 (Figs. 1B, 22D).

***Monocerophora*** **Walker, 1869**

(Figures 19, 20, 22B, S4)

#### Type species

Monocerophora longispina (Burmeister, 1838)

#### Redescription

**Head.** Eyes globose, located laterally, inserted at the level of the ventral margin of antennal sockets. Apex of antennal sockets as high as dorsal margin of eyes. Face apparently slender, due to the fastigium and frons that are protruding upward; vertex barely visible in frontal view; width six-tenths the length of the head. Frons long and triangular, usually exceeding the apex of sclerites of antennal sockets; lower portion of the frons with a defined ocellus. Face smooth. Mandibles robust, with tip elongated. Mandibles without a basal process at the cutting edge. **Thorax.** Pronotum dorsally with anterior and posterior margins slightly concave. Prozona with a transverse furrow extending to the lateral lobe, not reaching the lower margin. Anterior margin of the mesozona with a transverse furrow extending to the lateral lobe but not reaching the ventral margin. Metazona with a straight transversal furrow reaching the posterior margin of lateral lobes. Lateral lobes with ventral margin straight; posterior margin straight. Each sternite with two slender spines with rounded tips (Fig. 2D). **Wings.** Wings well-developed; tegmina without a bright spot. **Legs.** Coxae bearing acute or rounded spines on the ventral margin, one at the basal and another at the distal portion (Fig. 2D). Legs slender; hind femora as long as the body. Fore and mid femora ventrally sulcated with broad longitudinal furrow. Femora of all legs with both ventral margins armed with long spines. Fore tibiae with a small rounded pit below and close to each tympanal opening. Hind tibiae with several spines on the dorsal and the ventral margins. Genicular lobes of the legs ending in an acute tip. Tympanal openings located dorsally on the tibiae; openings fairly wide, the width one-tenth the length; area surrounding ear region not inflated. **Male postabdomen.** Cerci slightly curved downward; tubercles and bristles covering the entire surface (Fig. 4K). Supraanal plate triangular. Subgenital plate elongated, with a shallow emargination; styli as long as the plate (Fig. 5T). Phallus with a single titillator formed by a transversal bridge which arises from the basal portion of the dorsal lobe, and with two free projections oriented outward, with rounded tips (Fig. 3G–H). **Female postabdomen.** Female subgenital plate wide, with a short U-shaped emargination at the posterior margin (Fig. 5U, V). Ovipositor shorter than body length, mostly straight; upper valve about three times as wide as lower valve; apex of ovipositor acute (Figs. 19D, 20D).

This genus includes *M. longispina* (Burmeister, 1838), *M. minax* Walker, 1869, **reinstated status** and *M. spinosa* (Karny, 1907). Molecular results showed three clades defined by the COI gene (Fig. 7A): the first clade from southern Bahia (localities 1, 2 and 4, Fig. 1), corresponding to *M. minax* **reinstated status**; a second from Espírito Santo (ReBio de Sooretama, locality 9), where all specimens are immature, making species-level identification impossible (*Monocerophora* sp.) and the third from the border of Minas Gerais and Rio de Janeiro (PARNA do Itatiaia, locality 13), corresponding to *M. spinosa*. Although the type-locality of *M. spinosa* is the state of Espírito Santo, the precise locality is unknown. However, as the specimens collected in Espírito Santo are grouped in a different clade than those from Rio de Janeiro, at this time the best option is to consider them *Monocerophora* sp. Specimens from PARNA do Itatiaia are all females and morphologically similar to the described male *M. spinosa*. It is important to carry out additional surveys to confirm the identity and distribution of *M. spinosa* and *Monocerophora* sp.

***Monocerophora minax*** Walker, 1869, **reinstated status**

(Figures 19, 22B, S4A–C)

Redtenbacher [2] synonymized *M. longispina* and *M. minax* based on specimens from Rio de Janeiro. Males of *M. minax* bear conspicuous long spine-shaped frontal fastigium (in the frons), and in *M. longispina* the frons is only slightly longer than antennal sockets according to the original description. Additionally, specimens from Rio de Janeiro checked by Redtenbacher have a brown face, different from the blackish face of *M. minax*. As *Monocephora minax* was described based on a male, this is the first time that females are described and registered.

#### Diagnosis

This species can be distinguished from *M. spinosa* by the following combination of features: (i) fastigium projected forward, with a rounded tip, (ii) male with frons strongly projected upward, reaching the tip of the antennal pedicel; in female, frons is about one-third the length of antennal scape, (iii) face and mandibles blackish.

#### Redescription

(Based on images of the holotype of *M. minax* **♂**). Holotype **♂**: LT 33.80. **Head.** Face mostly smooth, central portion close to clypeal suture wrinkled. **Male postabdomen.** Cerci stout, the tip slightly curved inward. **Coloration.** Dorsal surface of head blackish. Sclerites of antennal sockets, antennal scape, pedicel and flagellum blackish. Eyes dark brownish with diffuse blackish spots. Face and clypeus blackish. Labrum with basal portion yellowish, the remaining blackish. Maxillary and labial palpi yellowish. Pronotum dark brownish. Tegmina mostly light brownish; primary and secondary veins light brownish; surface between veins darker; stridulatory area blackish. Fore and mid legs with the following coloration pattern: femora dark brownish with lighter apical portion, ventral portion and spines blackish; tibiae with basal portion yellowish, central portion light brownish, distal portion and spines blackish. Hind legs dark brownish; femora with a blackish stripe at the ventral margin, extending from base to middle; dorsal surface blackish. Tarsi dark brownish, the third one darker.

#### Observations on collected males and females

**Head.** Mandibles stouter in male than in female (Fig. 19C, F). Clypeus slightly wrinkled. **Wings.** Tegmina with stridulatory file as shown in Fig. 6K. **Male postabdomen**. Supraanal plate trapezoidal, with the posterior margin rounded (Fig. 4K). Cerci as shown in Fig. 4K. Subgenital plate twice as long as wide (Fig. 5T); styli slightly longer than the plate. **Female postabdomen.** Subgenital plate as shown in Fig. 5U. Ovipositor as long as abdomen (Fig. 19D). **Coloration.** Body mostly light to dark brownish and blackish. Fastigium laterally dark brownish. Eyes with inner dorsal surface light brownish. Clypeus with basal suture yellowish; basal portion blackish; disc and apical portion chestnut-colored; basal portion in the middle whitish. Pronotum with anterior, posterior and rear half of the lateral margins blackish. Sternum blackish, with furrows of meso- and metasternum whitish; spines dark brownish. Hind wings and veins yellowish. Tarsal claws light brownish with darker tips. First to third abdominal sternites in male light brownish, the remaining ones blackish; sternites dark brownish in females. **Alive**, specimens with lighter coloration. Eyes blackish, with inner dorsal surface lighter and separated by a yellowish stripe. Antennal scape with a longitudinal whitish stripe at the outer portion. Spiracle of the third thoracic segment yellowish, more conspicuous than the others. Costal margin of the tegmina greenish (Figs. S4A–C).

#### Variation

Measurements of males (n = 2, not including the holotype): TL 38.00–42.00; PL 8.00–11.00; PW 7.00–8.00; EyeW 4.00; TegL 45.00–55.00; SL 3.53–3.12; NT: 111–118; maxT 0.08–0.09; HF 35.00–37.00; HT 39.00–43.00; sFF, inner margin, large 4, outer large 3–4; sMF, inner margin, large 3, outer large 3; sHF, inner margin, large 8–11, outer large 8–9; sMTld 3; sHTd, inner margin 11–14, outer 10–12; sHTv, inner margin 16–20, outer 16–19. Measurements of females (n = 3): TL 36.00–43.00; PL 9.00–10.00; PW 6.00–8.00; EyeW 3.00–4.00; TegL 46.00–51.00; HF 34.00–35.00; HT 37.00–40.00; sFF, inner margin, large 4, outer large 4; sMF, inner margin, large 3, outer large 3; sHF, inner margin, large 9–10, outer large 10–11; sMTld 3; sHTd, inner margin 11–12, outer 10–12; sHTv, inner margin 16–17, outer 17–18; OL 29.00–35.00. Size of mandibles and head, and length of fastigium in males are distinctly variable (n = 4). Males may also have stouter mandibles, wider head, and a short frons tip that reaches only the apex of antennal scape.

#### Specimens examined

One adult male, four adult females and one immature female CELC) \Brasil, BA, Jussari, RPPN Teimoso. 7–9, I, 2012. J. Chamorro *leg*. [handwritten on white paper]\, with the respective codes \1A\, \2B\, \3A\, \3B\, \6\ and \5\ and additionally labeled/Listro/Teimoso [printed on white paper] \ *Monocerophora minax* Walker [printed on white paper]\. One adult male, three adult females, one immature female (CELC) \Brasil, BA, Camacan, RPPN Bonita. 10–12, I, 2012. J. Chamorro *leg*. [handwritten on white paper] \, with the respective codes \17\, \11\, \16\, \25\ and \18\ and labeled/Listro/Bonita [printed on white paper] \ *Monocerophora minax* Walker [printed on white paper]\. One adult male and one adult female (CELC) \Brasil, BA, Porto Seguro, P.N. Pau Brasil, J. Chamorro *leg*. [handwritten on white paper]\, with the respective codes \9A\ and \2\ and labeled/Listro/Pau [printed on white paper] \ *Monocerophora minax* Walker [printed on white paper]\. Two immature males and one immature female (CELC) \Brasil, BA, Prado, P.N. Descobrimento. 13–15, I, 2012. J. Chamorro *leg*. [handwritten on white paper]\, with the respective codes \3\, \8\, \12\ and labeled/Listro/Descobrimento [printed on white paper] \ *Monocerophora minax* Walker [printed on white paper]\.

#### Distribution

The specimens were sampled in RPPN Serra do Teimoso, RPPN Serra Bonita, Parque Nacional do Pau Brasil, and Parque Nacional do Descobrimento, localities 1, 2, 3 and 4 respectively (Figs. 1B, 22B). The accurate type locality of *M. minax* **reinstated status** is unknown. The only available information is: “Pernambuco”.

***Monocerophora spinosa*** **(Karny, 1907)**

*Listroscelis spinosa* Karny, 1907

(Figures 20, 22B, S4D–F)

#### Diagnosis

This species can be distinguished from *M. minax* **reinstated status** by the following combination of features: (i) fastigium triangular with rounded tip, and about one-third the length of the scape, surpassing the apex of sclerites of antennal sockets, (ii) frons triangular, as high as the antennal sockets, (iii) face dark brownish, and mandibles blackish.

#### Redescription

(Based on images of the holotype **♂**). Measurements of the holotype **♂**: TL 30.00; PL 9.00; TegL 50.50; FF 28.00; HF 32.00; HT 36.00. **Head.** Face smooth, as shown in Fig. 20C. **Male postabdomen**. Cerci slightly cylindrical and curved downward. **Coloration.** Body mostly light to dark brownish and blackish. Dorsal surface of head with median portion blackish, except for a fine light brownish line extending from the fastigium tip to the posterior portion of head, sides light brownish. sides and frontal portion blackish. Eyes light brownish with diffuse blackish spots; inner dorsal surface yellowish. Sclerites of antennal sockets, scape and pedicel blackish. Frons dark brownish, with a well-defined light yellowish ocellus. Face dark brownish. Mandibles blackish with basal portion dark brownish. Maxillary and labial palpi brownish. Pronotum dark brownish, with a blackish median stripe. Tegmina light brownish; primary and secondary veins light brownish, with surface between them dark brownish; coastal margin blackish. Fore and mid femora dark brownish, apical portion lighter. Femora with light brownish spines, each spine with a whitish spot at the outer basal portion. Hind femora with a dark brownish stripe at the outer margin. Tibiae light brownish, basal portion lighter. Fore and mid tibial spines with light brownish tips. Tarsi light brownish, the third tarsomere darker. Abdominal sternites dark brownish.

#### Observations on collected females

**Female postabdomen**. Subgenital plate as shown in Fig. 5V. **Coloration.** Body mostly light to dark brownish and blackish. Antennomeres of flagellum light brownish, distal margin of each antennomere with a dark brownish stripe. Clypeus dark brownish with whitish disc (Fig. 20F). Mouthparts ventrally yellowish, except for the light brownish labial palpi. Pronotum with a blackish mark on the posterior portion of lateral lobes. Sternum blackish; areas close to the furrows of the pro- and metasternum black. Hind wings light brownish, veins of the same color. Legs with coxae and trochanters dark brownish, with diffuse blackish spots. Fore coxae and trochanters darker. Abdominal sternites dark brownish and pleurites blackish. **Alive**, individuals (only females were observed) lighter. Eyes dorsally light yellowish. Disc of clypeus more vividly whitish. Tibiae reddish. Abdominal pleura light purple. Basal portion of ovipositor pinkish (Fig. S4E). Immature female as shown in Fig. S4F.

#### Variation

Measurements of females (n = 3): TL 33.00–39.00; PL 9.00; PW 7.00–8.00; EyeW 3.00–4.00; TegL 46.00–49.00; HF 16.00–32.00; HT 18.00–36.00; sFF, inner margin, large 4–5, outer 4–5 large; sMF, inner margin, large 3–4, outer large 3–4; sHF, inner margin, large 10–12; outer large 11–10; sMTld 3; sHTd, inner margin 11–13, outer 10–11; sHTv, inner margin 16–17, outer 17–17; OL 26.00–27.00.

#### Specimens examined

Three adult females and two immature females (CELC) \Brasil, RJ, Itatiaia, P.N. Itatiaia, 7–13, XII, 2011, J. Chamorro *leg*. [printed on white paper] \ *Monocerophora spinosa* (Karny, 1907) [printed on white paper]\, with the respective codes \2\, \4\, \7\, \1\ and \5\ and additionally labeled/Listro/Itatiaia [printed on white paper]\.

#### Distribution

The specimens were sampled in PARNA do Itatiaia, locality 13 (Figs. 1B, 22B). Individuals were captured mostly in Arecaceae plants. The accurate type locality of *M. spinosa* is unknown, the only available information being “Espirito Santo”.

#### Comment

As *M. spinosa* was described based on a male, this is the first time that the female is described and registered.

***Monocerophora*** **sp.**

(Figure 22B)

The distinction of *Monocerophora* sp. was based on molecular data (Fig. 7). The sampled individuals are all immature and, therefore, morphological identification is not possible at this moment.

#### Specimens examined

Five immature males (CELC) \Brasil, ES, Linhares, ReBio de Sooretama. 29–XI – 2–XII, 2011. J. Chamorro *leg.* [printed on white paper] \ *Monocerophora* sp. [handwritten on white paper] \, and with the respective codes \2\, \3\, \4\, \5\, \7\, and additionally labeled \Listro/Sooretama [printed on white paper]\.

#### Distribution

The specimens were sampled in ReBio de Sooretama, locality 9 (Figs. 1B, 22B).

**Terpandrini Gorochov, 1990**

The classification of *Megatympanon* is controversial. The genus was originally described by Piza [51] as a Tympanophorinae. Riek [52] considered that the metasternal spines are typical of Listroscelidinae and Saginae, but because of the presence of at least one dorsal apical spur on hind tibiae, *Megatympanon* was transferred to Listroscelidinae (as “Listroscelinae”). Gorochov [53] proposed a new tribe, named Terpandrini, inside Saginae and including *Neobarrettia* and *Terpandrus* Stål. Later, Rentz [54] proposed another new tribe also named “Terpandrini”, based on *Terpandrus* and a few Australian genera, but inside Listroscelidinae. The first proposal for the name Terpandrini is the valid one, and so Gorochov is the author of the tribe. The inclusion of *Terpandrus* in Listroscelidinae, rather than in Saginae, however, is the most recent opinion on the position of the genus and consequently transfers the tribe it names to Listroscelidinae. *Megatympanon* is very similar to some *Terpandrus* species [54]. Regardless of subfamily assignment, the monospecific *Megatympanon* is the only known genus of Terpandrini in South America. As we have not observed specimens of other genera of Terpandrini, a diagnosis of this tribe is not provided. However, a redescription of the species is necessary and is provided here, for future discussion on this matter.

***Megatympanon*** **Piza, 1958**

#### Type species

*Megatympanon speculatum* Piza, 1958

Description of *Megatympanon* is in Piza [51].

***Megatympanon speculatum*** **Piza, 1958**

(Figures 21, 22A)

#### Diagnosis

This species can be distinguished from the remaining Listroscelidinae by the following combination of features: (i) sclerites of antennal sockets touching in midline, concealing the tip of fastigium, (ii) Thoracic auditory spiracle oval and enlarged, partially covered by the paranotum, free from pronotum, (iii) each sternite with two spine-shaped processes, (iv) each tegmen with identical stridulatory area, (v) phallus devoid of titillator.

#### Redescription (holotype ♂, allotype ♀ & paratypes 2♂)

Holotype **♂**: TL 36.00; PL 11.00; TegL 68.00; HF 35.00. Allotype **♀**: TL 47.00; PL 12.50; TegL 42.00; HF 37.50. **Head**. Fastigium laterally compressed, narrower and shorter than the first antennomere of the flagellum (Fig. 21C, F). Eyes globose and laterally protruding, inserted close to the outer edge of antennal sockets. Apex of antennal sockets in frontal view as high as eyes. Fastigium of frons triangular. Face, genae and clypeus smooth. Mandibles not robust and without modifications. Maxillary and labial palpi yellowish. **Thorax.** Pronotum with anterior margin straight; posterior margin strongly convex. Mesozona covering bases of tegmina dorsally. Prozona with a transverse curved furrow, extending to the lateral lobe but not reaching the ventral or anterior margin. Mesozona with a V-shaped transverse sulcus, extending to the lateral lobes, not reaching the ventral margins. Metazona with a transverse furrow extending laterally, not reaching the ventral margin. Lateral lobes with ventral margins curved, posterior margins oblique; corners rounded. Each sternite with two long and acute spines (Fig. 2E). **Wings.** Wings developed. Tegmina devoid of bright spot; stridulatory file as shown in Fig. 6L. **Legs.** Legs slender. Hind coxae with ventral margin bearing one basal acute spine (Fig. 2E). Hind trochanter with ventral margin bearing one apical rounded spine (Fig. 2E). Hind femora as long as body. Fore and mid femora with a broad longitudinal ventral furrow. Both ventral margins of femora armed with spines interspersed with thin spines. Fore tibiae straight, devoid of spur or pit below tympanal openings. Mid tibiae with seven to nine spines. Hind tibiae with spines on dorsal and ventral margins. Each genicular lobe with two acute spines. Tympanal openings located in the dorsal plane of fore tibiae; ear region not inflated; tympanal openings narrowed, and partly covered by lateral sclerites. **Male postabdomen.** Cerci cylindrical and curved inward, with acute tips (Fig. 4L). Supraanal plate short and subtriangular (Fig. 4L). Paraprocts short, triangular, without modifications. Subgenital plate wide; apical portion with a V-shaped emargination (Fig. 5W). Styli short, about one-quarter the length of the plate. Phallus devoid of titillators. **Female postabdomen.** Subgenital plate with a U-shaped cut emargination about one-third the length of the plate (Fig. 5X). Ovipositor shorter than abdomen, slightly upcurved; upper valve about four times as wide as lower valve; apex of ovipositor acute, lower valve with apical portion serrate ventrally (Fig. 21D). **Coloration.** There is no information on color of live individuals. Old specimens deposited in museums are yellowish, slightly greenish.

#### Variation

Measurements of male including holotype (n = 3): TL 35.00–38.00; PL 11–12.00; PW 6.00; EyeW 4.00; TegL 67.00–69.00; HF 33.00–35.00; HT 34.00–36.00; sFF, inner margin, large 6–7 small 0–3, outer large 7, small 3–6; sMF, inner margin, large 7–8, small 0–5, outer large 7–9, small 0–3; sHF, inner margin, large 12–14, small 0, outer large 12–14, small 0; sMTld 3–4; sHTd, inner margin 26–31, outer 23–30; sHTv, inner margin 22–24, outer 20–24. Stridulatory file (n = 1): SL 5.56; NT 64; min 0.05; maxT 0.45. Measurements of females, including allotype (n = 2): TL 42.00–47.00; PL 12.00–12.50; PW 7.00; EyeW 5.00; TegL 39.00–42; HF 34.00–37.50; HT 36.00; sFF, inner margin, large 6, small 3–5, outer large 9–8, small 7–5; sMF, inner margin, large 7, small 3–8, outer large 6–7, small 6–9; sHF, inner margin, large 14, small 7, outer large 12, small 4; sMTld 2; sHTd, inner margin 28, outer 29; sHTv, inner margin 17, outer 16; OL 26.00.

#### Specimens examined

**Holotype male** (ESALQ) \BR. – S.P. – SALESÓPOLIS, BORACEA 6-?. MAR. 1948, TRAVASSOS F., BRAZ, RABELLO & BOKERMANN [typewritten on yellow paper] \ *Megatympanon speculatum* Piza Tipo [handwritten on yellow paper] \ MZLQ-I 0054, E. S. A. “Luiz de Queiroz”- U.S.P., ZOOLOGIA, Piracicaba – S.P., Brasil [hand- and typewritten on white paper]\. **Allotype female** (ESALQ) \ Rio. *Cerberodon viridis* Perty [handwritten on yellow paper] \ *Megatympanon speculatum* Piza alótipo [handwritten on yellow paper] \ MZLQ-I0054, E.S.A. “Luiz de Queiroz”- U.S.P., ZOOLOGIA, Piracicaba – S.P. Brasil [hand- and typewritten on white paper] \ 92.141 [typewritten on white paper]. **Two male paratypes** (ESALQ), with similar collection data and labels as the holotype, and additionally labeled \ *Megatympanon speculatum* Paratipo [handwritten on yellow paper] \ MZLQ-I 0054, E.S.A. “Luiz de Queiroz”- U.S.P., ZOOLOGIA, Piracicaba – S.P. Brasil [hand- and typewritten on white paper]\. **Non-type material.** One male (MNRJ) \ Petropolis E. Rio. Fev.- Março 1958. D'Albuquerque [handwritten on yellow paper] \ *Megatympanon speculatum* [handwritten on white paper]\; one male (MNRJ) \Le Vallon Alto Mosela. Petrópolis – II a III. 958. Dalcy. col. [handwritten on yellow paper] \ *Megatympanon speculatum* [handwritten on white paper]\. One female (MNRJ) \Serra da Caveira, 600 m. M. Itaguay. Est. do Rio. 25-2-1948. W. Zikán, col. [printed on yellowish paper] \. One male (IBB) \ Brasil, SP, Salesópolis, Est. Biol. De Boracéia 20–27. iv. 2011. F. A. G de Mello, col. CNPq-SISBIOTA [printed on white paper] \ *Megatympanon speculatum* [handwritten on white paper]\.

#### Distribution

Collections from Estação Biológica (Est. Biol.) de Boracéia and Petrópolis (Fig. 22A) are new records for the species. The species was originally described based on specimens from Salesópolis and Rio de Janeiro.

#### Nomenclatural changes for Listroscelidinae not recorded from the Atlantic Forest

We have located type specimens of *Cerberodon cuiabensis* Piza (ESALQ), a species not recorded from the Atlantic Forest. This species was previously described by Karny [4] as *Carliella mandibularis* Karny. In order to stabilize the nomenclature of Listroscelidinae, we proposed a new subjective synonym here.

***Carliella mandibularis*** **Karny, 1911**

*Cerberodon cuiabensis* Piza, 1982 **syn. nov.**

*Carliella* differs from *Cerberodon* and the other genera by having the vertex of the head not protruding, face slightly wrinkled, pronotum without a deep furrow at the metazona and an abdomen with blackish sternites. Male cerci are stout, with apical portion abruptly curved inward. Male subgenital plate is as wide as long, with a short U-shape emargination. Female subgenital plate is short and triangular, without emargination.

#### Distribution

Male and female *Cerberodon cuiabensis* from the original type series were collected at Cuiabá, Mato Grosso, Brazil, the same type-locality where the holotype of *C. mandibularis* was collected. It was assigned to the wrong genus, and its morphological characteristics clearly place it in *Carliella*. Type material of *C. cuiabensis* was examined by JCR in 2008 at Museu de Entomologia da ESALQ (Piracicaba, Brazil), and images are available in OSF.

#### Classification and composition of Listroscelidinae

In this work, we proposed two main modifications for classifying Listroscelidinae: (i) Hamayulini **trib. nov.**, comprising *Hamayulus* **gen. nov.**, which does not fit in any of the current tribes of the subfamily and forms an independent group in our COI tree (Fig. 7A); (ii) We added *Carliella*, *Cerberodon*, *Isocarliella*, *Macrometopon* and *Monocerophora* to Listroscelidini, which previously consisted of *Listroscelis* and is partially equivalent to Listroscelidinae sensu Gorochov [55] that included *Carliella*, *Cerberodon*, *Listroscelis* and *Monocerophora*. Hereafter, the term “Listroscelidini” will refer to the tribe as defined here. The second column of Table 1 summarizes our proposed classification of Listroscelidinae. We based our new classification on several key morphological aspects of Listroscelidinae:

#### Head

In Listroscelidini, the head is elongated, which is most evident in frontal view. In this tribe and in *Neobarretia* (Terpandrini), the labrum and mandibles are long. Indeed, the shape and development of the mandibles, which are related to sexual dimorphism and occur in other katydids, are outstanding features in Listroscelidini. It is unknown whether this dimorphism is an ancestral condition retained by few living taxa or has independently arisen in different groups. Such features are also observed in *Arachnoscelis*, which has different developmental degrees of the mandibles. However, the way of development, as described by us, differs within Listroscelidini. Also, the mandible in *Carliella*, *Cerberodon*, *Listroscelis* and *Hamayulus* **gen. nov.** (Hamayulini **trib. nov.**) bears a large, basal, ventral process, a state unique among Tettigoniidae [21], while *Monocerophora*'s mandibles lacks this process, which may be a recent evolutionary loss. **Fastigium.** Listroscelidini, as well as the only North American listroscelidine *Neobarrettia*, have a compressed and narrowed fastigium. *Monocerophora* has an overdeveloped frons (as a result, in *M. minax* **reinstated status**, the frons is slightly directed upward), which has triangular or subtriangular shape; while in *Cerberodon*, *Listroscelis* and *Isocarliella*, the frons is a sulcated triangle, with the central portion protruding. In Terpandrini (*Neobarretia* and *Megatympanon*) and *Arachnoscelis* (incertae sedis), the fastigium is not markedly differentiated. In Terpandrini, the fastigium grows from the anterior portion of the occiput, between antennae (in dorsal view). In *Hamayulus* **gen. nov.** the fastigium is sulcated. **Vertex.** The vertex is remarkably protruded, mainly in *Cerberodon* (Listroscelidini), *Neobarretia* (Terpandrini) and *Hamayulus* **gen. nov.** (Hamayulini **trib. nov.**). It is related to the frontal or frontolateral position of eyes and possibly an adaptation to predatory habits. The position of eyes is similar to that observed in predatory Meconematinae, Saginae and Hexacentrinae.

#### Thorax

In Listroscelidini, the metazona is typically elevated from the sulcus, which is more evident in *Monocerophora* but also occurring in *Neobarrettia* (Terpandrini). In other Terpandrini, such as *Megatympanon*, *Burnuia* Rentz and *Chlorobalius* Tepper, the metazona is so elongated that it covers the basal dorsal portion of wings. **Auditory Spiracle.** The auditory spiracle of Listroscelidini is typical of Conocephaloid [8]: elongated and with a wide opening, free from pronotum and completely exposed; while in *Megatympanon* the spiracle is partially covered by the lateral lobes of pronotum. **Sterna.** Listroscelidinae are characterized by the presence of sternal spines. Within Listroscelidini, except for *Monocerophora*, the spines on the pro- and mesosternum are slender, and on the metasternum they are usually a bit flattened and similar to the ones of *Neobarrettia* (Terpandrini) species. In *Monocerophora*, all spines are slender with rounded tip; and in *Megatympanon* (Terpandrini), they are long and acute. Listroscelidini also have another type of armature at the ventral margin of coxae, each possessing one spine at the distal border and another at the proximal border, with different degrees of development. Sternal and coxal spines can be used for catching preys [13], [56].

#### Legs

Only Listroscelidini have the fore tibiae notably curved with long movable spines, which are longer than the spines in Terpandrini species. Listroscelidini species also have two dorsal tiny spurs or rounded pits below each tympanum, one close to the inner and the other close to the outer tibial margin, which is an important characteristic of the tribe. Although living and preserved individuals are usually devoid of such dorsal spurs (we observed only one *L. cohni* **sp. nov.** with intact spurs), it is possible to observe their small attachment cavities (pits) and confirm their existence. Cohn (1957) reported that none of the Listroscelidinae checked by him (*Cerberodon*, *Listroscelis* and *Macrometopon*) bore intact dorsal spurs, which confirms that these structures are easily lost. In *Hamayulus* **gen. nov.**, as in Decticini and *Neobarrettia*, there is only one spur (or cavity) close to the outer margin, while in *Megatympanon* there are neither spurs nor cavities. Cohn [13] stated that it is not clear whether the presence of two spurs below each tympanum is a derived and convergent or a more ancestral feature. In the latter case, it would have been retained in Listroscelidini and independently lost in other Listroscelidinae tribes. At least in Listroscelidini + Hamayulini **trib. nov**., the couple of spurs may be an ancestral condition. The ventral spines on all femora interspaced with minute spines is also a characteristic of Listroscelidini, but it is different in *Monocerophora*. **Tympana.** In most Listroscelidini, tympanal openings are elongated and wide, usually located at the dorsal surface of fore tibiae; while in Terpandrini, the openings are long, but narrow and barely visible. The tympana in *Hamayulus* **gen. nov**.are most similar to those of *Arachnoscelis*.

#### Postabdomen

Cerci, supraanal and subgenital plates are very variable among Listroscelidini species. Some patterns were found at genus level, e.g. *Monocerophora* and *Cerberodon*, but there are no apparent patterns in *Listroscelis*. Male paraprocts and phallus are remarkable shared characteristics: the paraprocts are wide, concealing the membranous portion of the phallus, and bear a tiny spine at the outer vertex; and the phallus consists of a conspicuous dorsal lobe and developed titillators. Cohn [13] dissected the phallus of a *Macrometopon* species, but did not describe its titillators. We decided to include *Macrometopon* in Listroscelidini because its single species has modified paraprocts, as in other species of the tribe, while in Terpadrini the paraprocts are simple. **Ontogenetic differences.** It is difficult to differentiate immature individuals of *Listroscelis*, *Monocerophora* and *Cerberodon*. While immature of *Listroscelis* and *Monocerophora* have a dark longitudinal midline at the pronotum, absent in adult *Listroscelis* (Figs. S3, S4), the asymmetric lengthened mandibles of *Cerberodon* and some species of *Listrosceli*s are exclusive of adults. The lack of differentiation among immatures suggests a common origin of these genera (supporting their inclusion within the same tribe), while the differences among adults probably result from chronologic developmental differences. Curiously, the first characteristic cited, the dark longitudinal midline at the pronotum, is conserved in adults of other subfamilies of Tettigoniidae, such as Conocephalinae and Pseudophyllinae, as well as in *Meconema* Serville (Meconematinae), a genus that Zeuner [9] pointed out as a sister group of *Listroscelis*. This author suggested that the morphological differences between these genera are superficial and result only from different feeding habits.

#### Further taxonomic comments

The next logical step in the taxonomic revision of Listroscelidini is to review Terpandrini. As indicated, *Neobarrettia* is more similar in several features to Listroscelidini than to Terpandrini. In addition, it is the only North American genus of the tribe, while the others (except for the Brazilian *Megatympanon*) occur in Australia. The similarities between *Megatympanon* and *Terpandrus* (from Australia) were first exposed by Rentz [54].

### Identification key to Listroscelidinae of the Brazilian Atlantic Forest

1 Sclerites of the antennal sockets in contact at midline. Tympanal openings narrow and concealed by lateral sclerites. Fore tibiae without a small pit or spur dorsally, below and close to each tympanal opening. Sternal spines ending in an acute tip. Paraprocts simple, triangular, devoid of spines… ***Megatympanon speculatum*** (Figs. 2E, 21)

1′ Sclerites of the antennal sockets not in contact at midline. Tympanal openings wide, not concealed by lateral sclerites. Fore tibiae with a small pit or spur dorsally, below and close to each tympanal opening (two or only one at the outer side). Sternal spines ending in a rounded tip. Paraprocts modified… **2**

2 (1′) Paraprocts modified, the whole outer portion spine-like, or as a long process. Fore tibiae with a small pit or spur dorsally, below and close to the outer tympanal opening… ***Hamayulus rufomaculatus*** **sp. nov.** (Figs. 4A, 8)

2′ Paraprocts triangular or subtriangular, with a tiny spine at the vertex of the outer portion. Fore tibiae with a small pit or spur dorsally, below and close to each tympanal opening… **3**

3 (2′) Face strongly wrinkled, brain-like. Male's left mandible with apical portion strongly elongated and curved upward. Female's subgenital plate elongated, with a deep V-shape emargination (***Cerberodon***, Figs. 9, 10, 5D, F)… **5**

3′ Face smooth or barely wrinkled, the wrinkles being linear, never brain-like. Male's left mandible with apical or lateral portion elongated or not. Female's subgenital plate without a deep V-shape emargination… **4**

4 (3′) Basal portion of tegmina with a bright yellowish spot. Mandibles symmetric or asymmetric. If asymmetric, the lateral portion of the left mandible is conspicuously enlarged (***Listroscelis***, Figs. 11–18)… **6**

4′ Basal portion of tegmina devoid of yellowish spot. Mandibles ever symmetric (***Monocerophora***, Figs. 19–20)… **15**

5 (3) Fore tibiae with ventral area blackish; tegmina, in both sexes, surpassing the length of the abdomen… ***C. viridis*** (Figs. 9, S2A–F)

5′ Fore tibiae with ventral surface dark brownish (in dead specimens) or dark orange (live specimens); tegmina shorter than abdomen, not covering the last two abdominal tergites in males and the last five abdominal tergites in females… ***C. portokalipes*** **sp. nov.** (Figs. 10, S2G–L)

6 (4) Face darker than the lateral portion of the head; scape and pedicel never blackish… ***L. ferruginea***

6′ Face as dark as the lateral portion of the head. If darker, scape and pedicel blackish… **7**

7 (6′) Fore femora with a blackish stripe at the ventral margin… **8**

7′ Fore femora without a blackish stripe at the ventral margin… **9**

8 (7) Pronotum with a blackish stripe at the posterolateral margin… ***L. itatiaia*** **sp. nov.** (Fig. 18)

8′ Pronotum without a blackish stripe at the posterolateral margin… ***L. carinata*** (Fig. 11)

9 (7′) Mandibles asymmetric, in which the left mandible has the preapical lateral portion elongated and bent, and apical portion projected upward… **10**

9′ Mandibles symmetric… **13**

10 (9) Surface of body mostly brownish; femora with ventral surface not blackish… ***L. fusca*** **sp. nov.** (Fig. 16)

10′ Body mostly chestnut-colored; femora with ventral surface blackish… **11**

11 (10′) Length of tegmen 21.00–23.00 mm… ***L. angustifrons*** **comb. nov.** (Fig. 12)

11′ Length of tegmen 13.00–14.00 mm… **12**

12 (11′) Tegmina completely chestnut-colored… ***L. atrata***

12′ Tegmina mostly dark greenish…. ***L. magnomaculata*** (Fig. 13)

13 (9′) Abdominal tergite X unmodified, short… ***L. cohni*** **sp. nov.** (Fig. 4H)

13′ Abdominal tergite X enlarged… **14**

14 (3′) Abdominal tergite X markedly tapering from middle to apex… ***L. sooretama*** **sp. nov.** (Fig. 4G)

14′ Tenth abdominal tergite not tapering to apex… ***L. monnei*** **sp. nov.** (Fig. 4J)

15 (4′) Frons projected like a spine, surpassing the length of antennal scape or only reaching its tip; face blackish… ***M. minax*** **reinstated status** (Fig. 19)

15′ Frons triangular, acuminated, the tip reaching only the apex of sclerites of antennal sockets. Face brownish… ***M. spinosa*** (Fig. 20)

## Discussion

After more than a century since Litroscelidinae katydids were recognized as a suprageneric taxon, they remain barely known to science, which may be attributed to the difficulties in collecting these animals. In addition, some species are aggressive (e.g. *Monocerophora* and *Cerberodon* species) and others are very fast (e.g. *Hamayulus* spp.), making it difficult to observe and collect them in the field. In addition to the high number of individuals collected (104), our samples had approximately five females to each male, making it difficult to identify and describe the species, which are preferably identified by males. No males of *L. itatiaia* **sp. nov**., *L. carinata* **sp. nov**. or *M. spinosa* were collected.

Despite these difficulties, we identified 14 distinct morphospecies, 12 of which have been treated taxonomically here. We collected a maximum of three genera from each sampled site. Except for *Listroscelis monnei* **sp. nov**. and *L. cohni* **sp. nov**. (both collected in locality 4; see Figs. 1B, 22C–D), each genus was represented by a single species in the same sample site, indicating a high rate of endemism of Listroscelidini in Atlantic Forest remnants. In fact, all Listroscelidinae species sampled in this work have a narrow distribution and were primarily collected in well-preserved forest remnants with low anthropogenic impacts, especially in areas close to water bodies, 50 cm to 2 m above the ground. The restricted distribution and high rate of endemism indicate that Listroscelidinae species may be at serious risk of extinction.

Our 18S tree contains the 14 morphospecies recognized here, while the COI tree contains only 12, since we found evidence for the presence of numts in many individuals and eliminated them to avoid confusion [37], [57]. In order to circumvent these problems, we are investigating the specimens suspected of containing numts to quantify the numts and pursue a strategy for sequencing only mitochondrial sequences (Fialho & Yotoko, unpublished data).

Although limited by the smaller number of taxa, the COI tree shows evidence of 13 distinctive mitochondrial lineages, as shown in Fig. 7A. Moreover, these strains are well separated into genera and suggest two tribes within Listroscelidinae: Listroscelidini (composed by *Cerberodon*, *Monocerophora* and *Listroscelis*) and Hamayulini **trib. nov**. (composed of two species of *Hamayulus* **gen. nov**.). In turn, all sequences of *Monocerophora* and *Listroscelis* were identical in the 18S tree (Fig. 7B). The topology differences between genes used here were expected and resulted from inherent differences in mitochondrial and nuclear genomes. While mitochondrial DNA have higher rates of nucleotide substitution, which makes it an excellent tool to distinguish closely related species [58], [59]; nuclear DNA evolves more slowly, making it useful for resolving deeper phylogenetic branches [60], [61].

Beyond confirming our morphological findings, our molecular results gave us clues regarding a major division in Listroscelidinae. Both sequences revealed two significantly different lineages within *Hamayulus* **gen. nov**. (although both strains were collected in Bahia, most in locality 4, with only one exception, collected at site 3). That the sequences of 18S revealed differences between strains of *Hamayulus* **gen. nov**, while no differences were detected between *Listroscelis* and *Monocerophora*, indicates that the separation of these strains must be very ancient, and we are possibly dealing with two distinct genera. Unfortunately, we have collected only immature individuals of *Hamayulus* sp., therefore new sampling will be needed to test this hypothesis.

The COI tree allowed us to suggest *Monocerophora* be subdivided into four distinct mitochondrial lineages: *M. spinosa* (sampled at locality 13), two different lineages within *M. minax* **reinstated status** (the first sampled in localities 1 and 2 and the second in locality 4) and a lineage named *Monocerophora* sp. (sampled in locality 9). Neither *Monocerophora* sp. nor the second lineage of *M. minax* **reinstated status** were morphologically identified because all individuals were immature.

A different result was found in the *L. fusca* and *L. itatiaia* pair. In this case, the diagnosis based on morphology is clear, but the molecular differentiation is subtle. Again, these species were collected in different localities (10 and 13), suggesting geographic distribution should be taken into account in taxonomic studies of Listroscelidinae.

Our dendrogram (Fig. S6), based on the region chosen as the DNA Barcode (final portion of the COI, Fig. S5) showed significant differentiation between species [44], and is very similar to the phylogenetic hypothesis of Fig. 7A. This result indicates it is possible to identify known species of Listroscelidinae with one sequencing reaction using the primer COI_Orth_1R (Table S1).

Though powerful, DNA Barcoding should be used with caution. Morphological analyses are laborious and require extensive training. Our results suggest convergence of morphological and molecular results. Therefore, we recommend sequencing the DNA Barcode region of any new listroscelidine specimen, but if any evidence of numts is detected (see the checklist suggested by Cristiano et al. [37]), the sequence should be removed from the analysis and treated separately.

Taxonomy has much to gain from combining morphological and molecular approaches, provided they are done conscientiously, so that technical complications (such as the possibility of numts, for instance) do not affect the quality of final results. Such an approach can boost taxonomic studies within this group, allowing studies on the diversity and distribution of listroscelidine species in the Atlantic Forest and other biomes in a relatively short time.

## Conclusions

This is the first broad taxonomic and molecular work on Neotropical Listroscelidinae. Based on specimens collected in the Brazilian Atlantic Forest, as well as some specimens deposited in museums, we added a new tribe, a new genus and eight new species to the subfamily and redefined Listroscelidini. We redescribed and added new geographic records for six species, synonymized two species and built a provisional identification key for Listroscelidinae occurring in the Atlantic Forest. Only four Listroscelidinae genera remain unclassified: *Arachnoscelis*, with six described species from southern Central America and the westernmost portion of the southern Neotropics; the monospecific *Liostethomimus* Karny, described from southern Brazil (but the type-specimen is considered lost) and the monospecific *Paralistroscelis* Carl and *Poecilomerus* Karny, both occurring in Madagascar.

Our results suggest that the species of this subfamily, at least those restricted to the Atlantic Forest, are seriously endangered. This is because they have high rates of endemism, restricted distribution and occur mostly in highly preserved forest remnants. As such, it is important that future collection efforts take into account the use of barcodes in this article, in order to accelerate the identification of known species and the detection of possible new ones.

## Supporting Information

Figure S1 **Live individuals of** ***Hamayulus rufomaculatus*** **sp. nov.** (A–C) Male, (D) Female.
(TIF)

Figure S2 **Live individuals of** ***Cerberodon*** **Perty.** A–F *Cerberodon viridis* Perty, (A–C) Female adult, (D–E) Immature female, (F) Immature male immature. G–L *Cerberodon portokalipes* **sp. nov.**, (G–J) Holotype male, (K–L) Allotype female.
(TIF)

Figure S3 **Live individuals of** ***Listroscelis*** **Serville.** A–C *L. carinata* Karny, female. D–F *L. magnomaculata* **sp. nov.**, (D) Female, (E–F) Male. G–H *L. sooretama* **sp. nov.**, female. I–J *L. fusca* **sp. nov.**, male. K *L. monnei* **sp. nov.**, female. L *L. itatiaia* **sp. nov.**, female.
(TIF)

Figure S4 **Live individuals of** ***Monocerophora*** **Walker.** A–C *M. minax* Walker, **reinstated status** (A–B) Male, (C) Female. D–F *M. spinosa* (Karny), (D–E) adult female, (F), immature female.
(TIF)

Figure S5 **COI fragment primers position.** Schematic presentation of primers used to amplify the COI fragment effectively used in Fig. 7A and the fragment offered as DNA Barcode of Listroscelidinae. Numbers correspond to the position in the complete mitochondrial genome of the species *Oxya chinensis* (Thunberg) (Orthoptera: Acrididae; GenBank: NC010219.1).
(TIF)

Figure S6 **Listroscelidinae Barcode dendrogram.** Dendrogram based on Barcode region (Fig. S1) using the Neighbor-joining method [45] with the substitution model Kimura 2-parameter [46]. Values alongside internal nodes correspond to bootstrap values, calculated through 1000 replications. Vertical bars mean that a set of sequences belong to one species or genus, identified at right of each bar. Numbers besides species' names correspond to sampled localities (Fig. 1). Outgroup: *Oxya chinensis* (Thunberg) (GenBank: NC010219.1).
(TIFF)

Table S1 **Primers sequences used in this work.**
(DOCX)

Table S2 **Geographic coordinates of the sampled conservation units.** Locality numbers are the same shown in Fig. 1.
(DOCX)
