# Supplementary figures and images for: Systematics of Spiny Predatory Katydids (Tettigoniidae: Listroscelidinae) from the Brazilian Atlantic Forest Based on Morphology and Molecular Data
Source: PLoS One. 2014 Aug 13;9(8):e103758. doi: 10.1371/journal.pone.0103758 (PMC4131907; doi:10.1371/journal.pone.0103758)

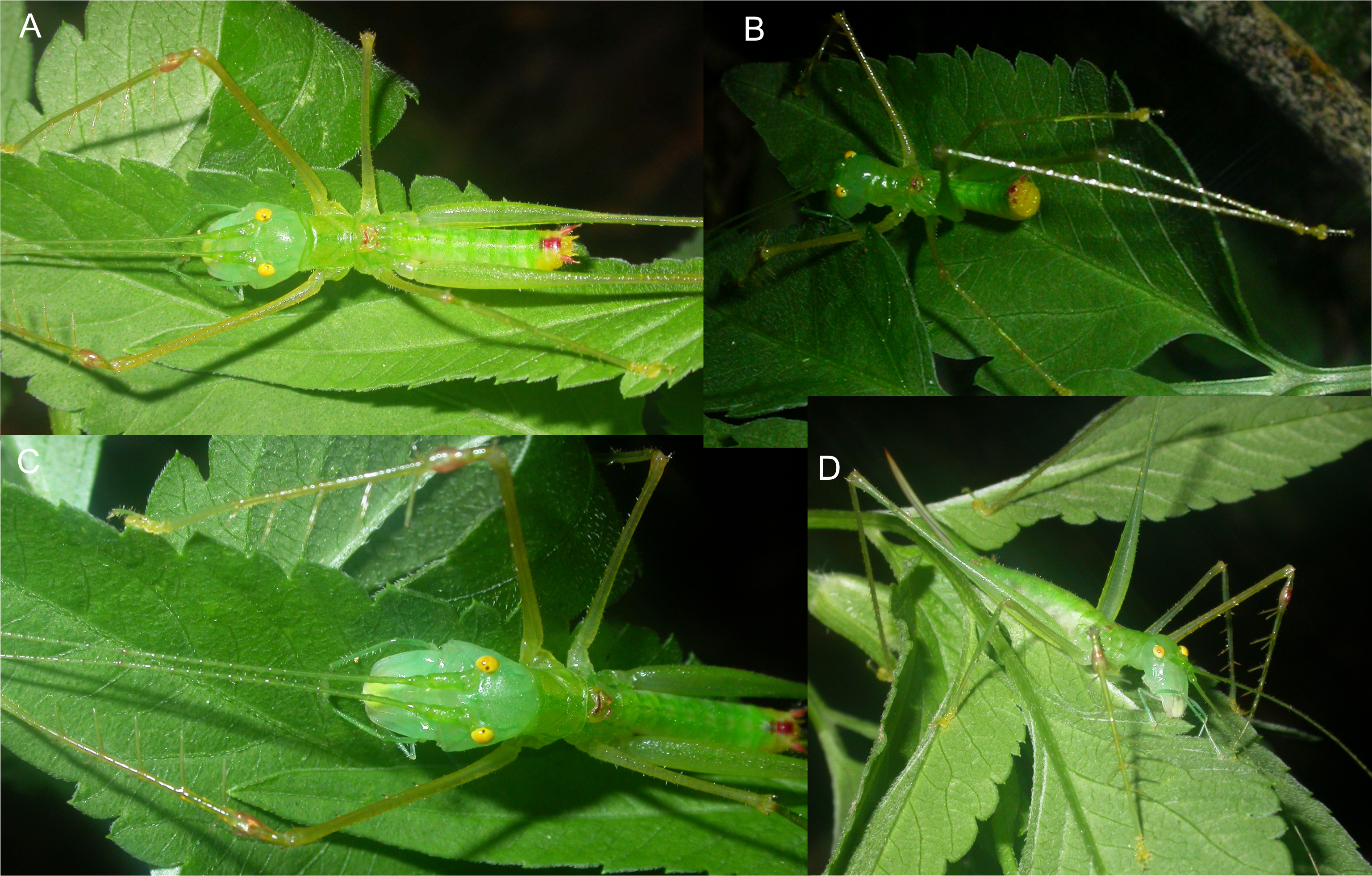

Supplement: Figure S1 — Live individuals of Hamayulus rufomaculatus sp. nov. (A–C) Male, (D) Female. (TIF) [file pone.0103758.s001.tif]

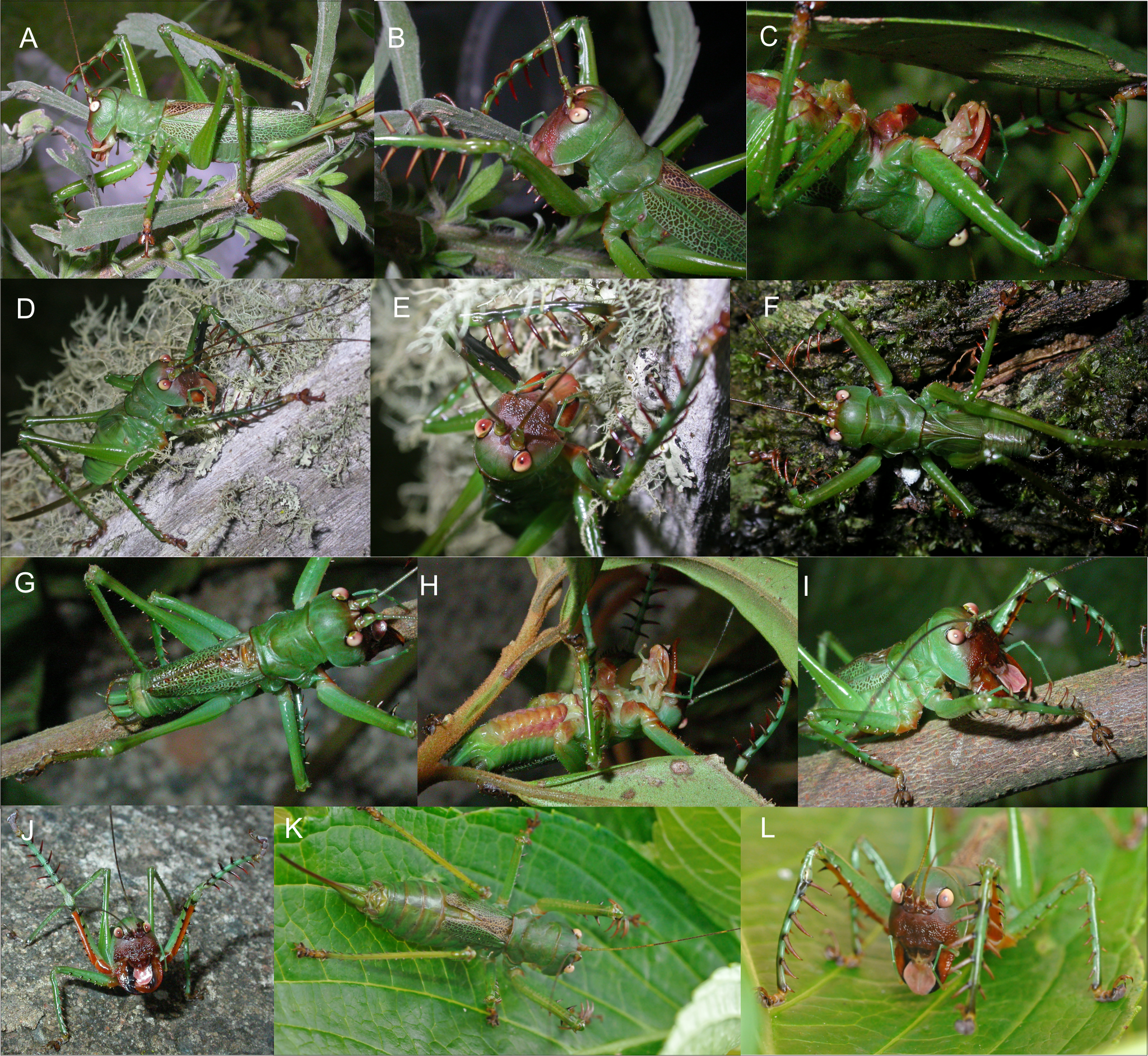

Supplement: Figure S2 — Live individuals of Cerberodon Perty. A–F Cerberodon viridis Perty, (A–C) Female adult, (D–E) Immature female, (F) Immature male immature. G–L Cerberodon portokalipes sp. nov., (G–J) Holotype male, (K–L) Allotype female. (TIF) [file pone.0103758.s002.tif]

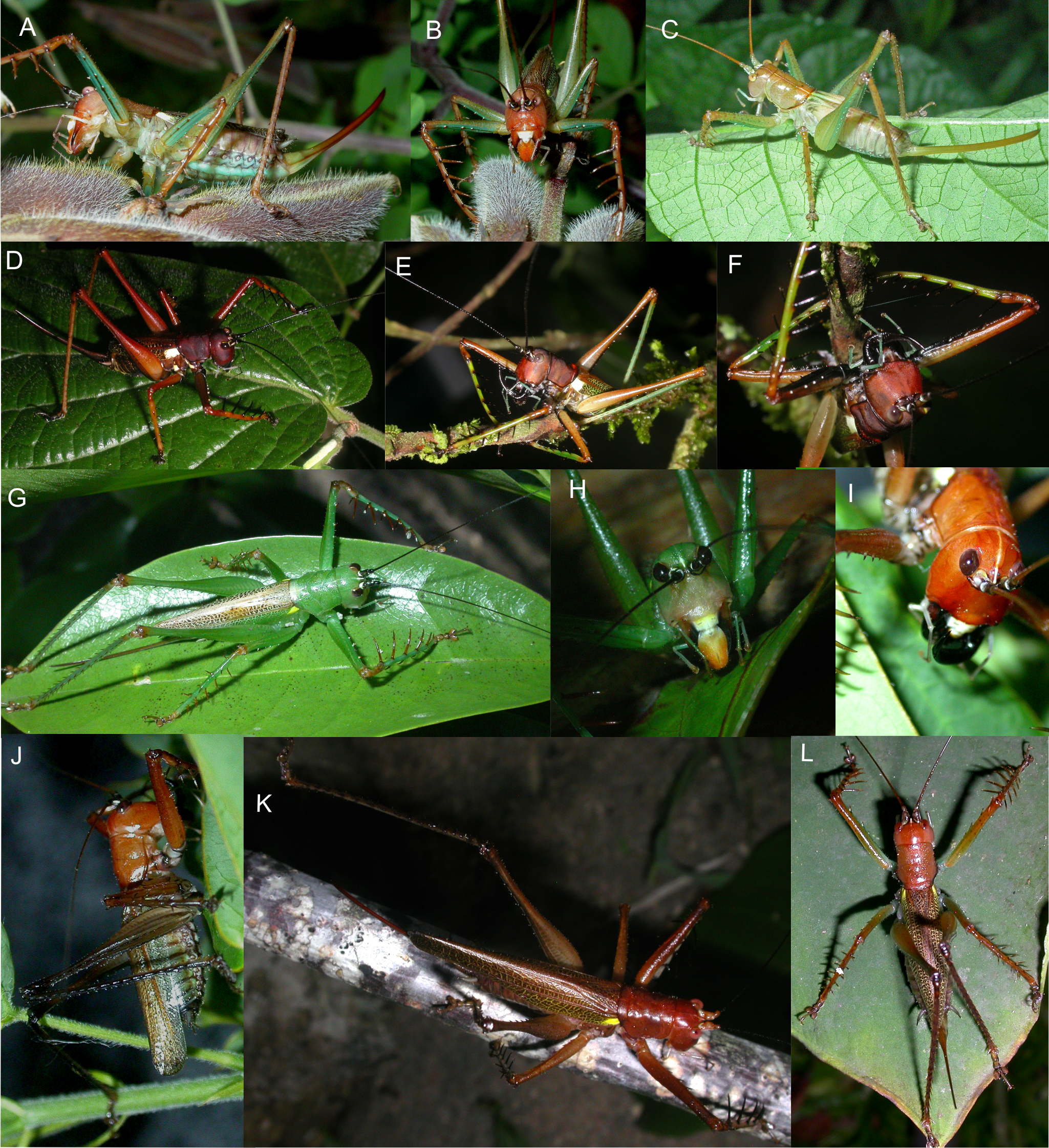

Supplement: Figure S3 — Live individuals of Listroscelis Serville. A–C L. carinata Karny, female. D–F L. magnomaculata sp. nov., (D) Female, (E–F) Male. G–H L. sooretama sp. nov., female. I–J L. fusca sp. nov., male. K L. monnei sp. nov., female. L L. itatiaia sp. nov., female. (TIF) [file pone.0103758.s003.tif]

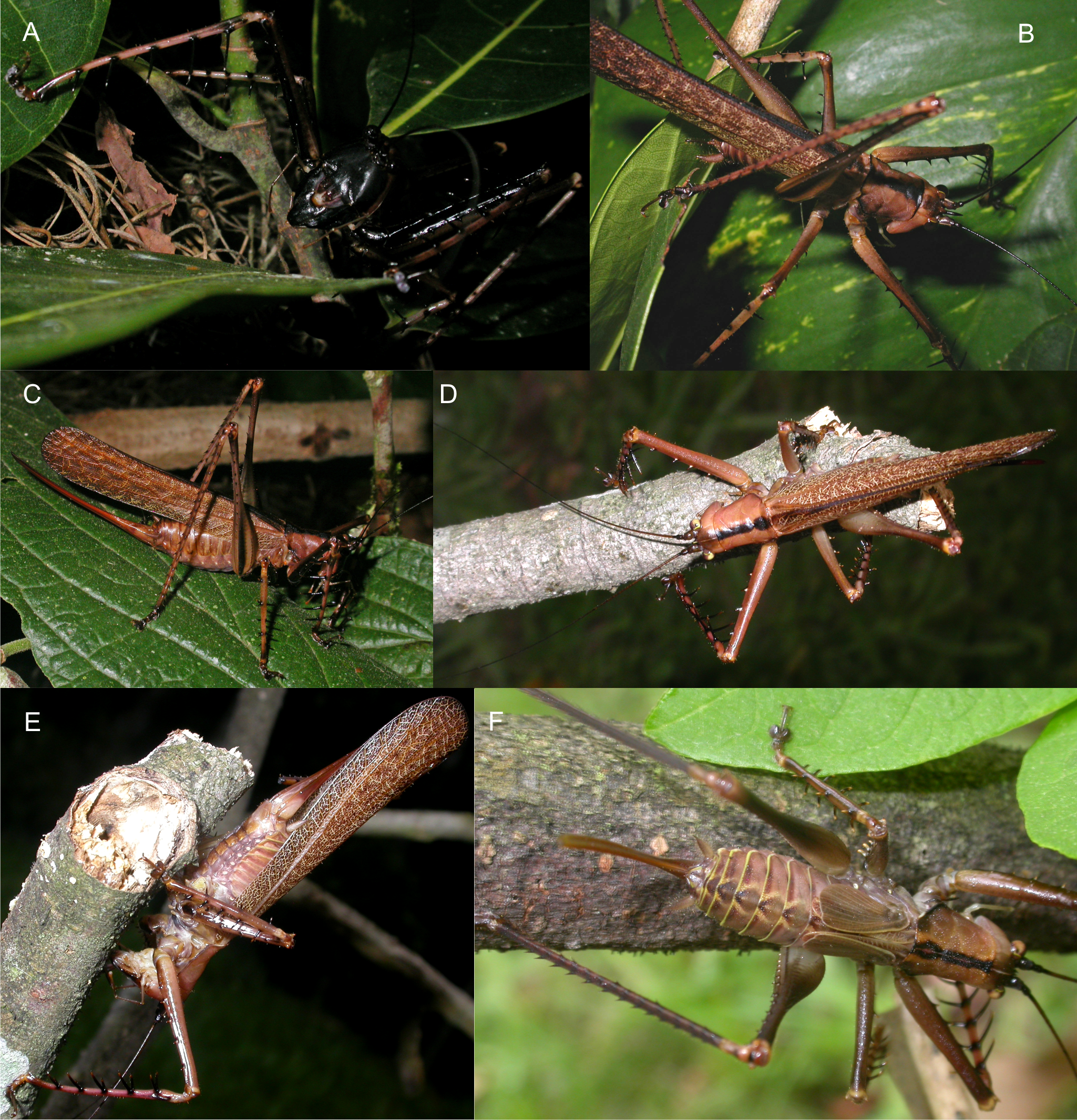

Supplement: Figure S4 — Live individuals of Monocerophora Walker. A–C M. minax Walker, reinstated status (A–B) Male, (C) Female. D–F M. spinosa (Karny), (D–E) adult female, (F), immature female. (TIF) [file pone.0103758.s004.tif]

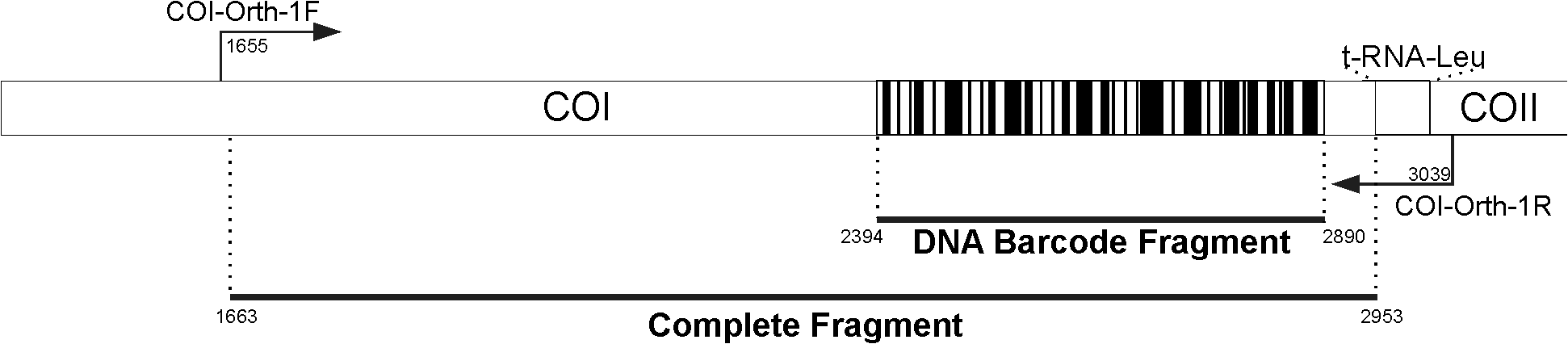

Supplement: Figure S5 — COI fragment primers position. Schematic presentation of primers used to amplify the COI fragment effectively used in Fig. 7A and the fragment offered as DNA Barcode of Listroscelidinae. Numbers correspond to the position in the complete mitochondrial genome of the species Oxya chinensis (Thunberg) (Orthoptera: Acrididae; GenBank: NC010219.1). (TIF) [file pone.0103758.s005.tif]

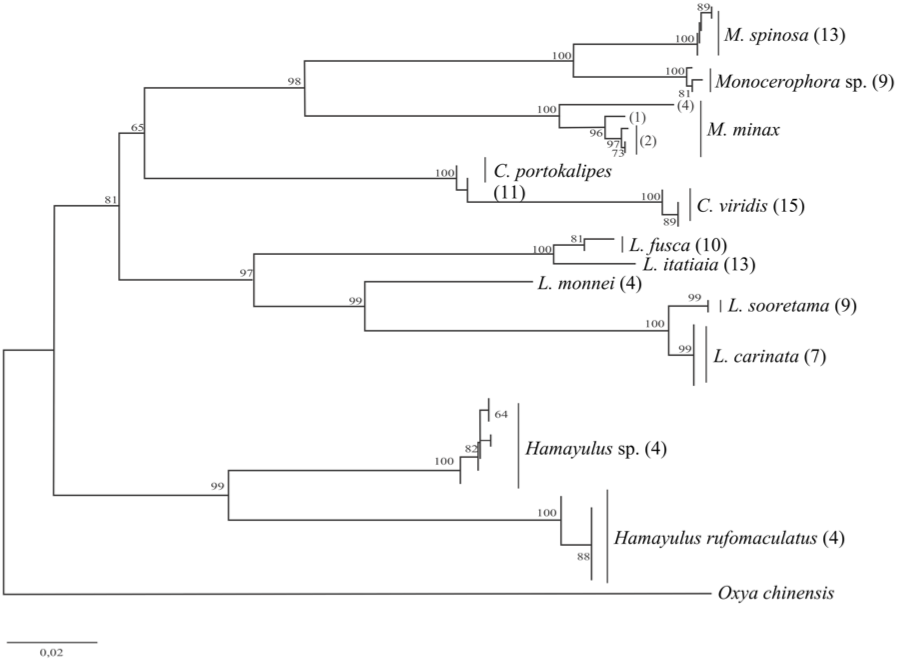

Supplement: Figure S6 — Listroscelidinae Barcode dendrogram. Dendrogram based on Barcode region (Fig. S1) using the Neighbor-joining method [45] with the substitution model Kimura 2-parameter [46]. Values alongside internal nodes correspond to bootstrap values, calculated through 1000 replications. Vertical bars mean that a set of sequences belong to one species or genus, identified at right of each bar. Numbers besides species' names correspond to sampled localities (Fig. 1). Outgroup: Oxya chinensis (Thunberg) (GenBank: NC010219.1). (TIFF) [file pone.0103758.s006.tiff]
