# Supplementary material for: Systematics of Spiny Predatory Katydids (Tettigoniidae: Listroscelidinae) from the Brazilian Atlantic Forest Based on Morphology and Molecular Data
Source: PLoS One. 2014 Aug 13;9(8):e103758. doi: 10.1371/journal.pone.0103758 (PMC4131907; doi:10.1371/journal.pone.0103758)
Supplement: Table S1 — Primers sequences used in this work. (DOCX) [file pone.0103758.s007.docx]

**Table S1.** **Primers sequences used in this work.**

| **Primer name** | **Primer sequence 5’ – 3’** |
| --- | --- |
| COI_Orth_1F | ATACCTATTATAATTGGAGG |
| COI_Orth_1R | TARRTTTRATCAKGTWGCCA |
| 18S_Orth_1F | CGCGAATGGCTCATTAAATC |
| 18S_Orth_1R | CCTCACTAAATCATTCAATCGG |
