# Supplementary material for: Systematics of Spiny Predatory Katydids (Tettigoniidae: Listroscelidinae) from the Brazilian Atlantic Forest Based on Morphology and Molecular Data
Source: PLoS One. 2014 Aug 13;9(8):e103758. doi: 10.1371/journal.pone.0103758 (PMC4131907; doi:10.1371/journal.pone.0103758)
Supplement: Table S2 — Geographic coordinates of the sampled conservation units. Locality numbers are the same shown in Fig. 1. (DOCX) [file pone.0103758.s008.docx]

**Table S2.** **Geographic coordinates of the sampled conservation units.** Locality numbers are the same shown in Fig. 1.

| **Locality** | **Conservation Unit** | **Coordinate** | |
| --- | --- | --- | --- |
|  |  | **Latitude** | **Longitude** |
| 1 | Reserva Particular do Patrimônio Natural Serra do Teimoso | -15.133 | -39.517 |
| 2 | Reserva Particular do Patrimônio Natural Serra Bonita | -15.046 | -39.565 |
| 3 | Parque Nacional do Pau Brasil | -16.493 | -39.251 |
| 4 | Parque Nacional do Descobrimento | -17.100 | -39.330 |
| 5 | Floresta Nacional do Rio Preto | -18.357 | -39.851 |
| 6 | Reserva Particular do Patrimônio Natural Sítio do Zaca | -19.419 | -42.620 |
| 7 | Parque Estadual do Rio Doce | -19.760 | -42.610 |
| 8 | Estação Biológica Santa Lúcia | -19.968 | -40.545 |
| 9 | Reserva Biológica de Sooretama | -19.055 | -40.146 |
| 10 | Parque Estadual Serra do Brigadeiro | -20.720 | -42.470 |
| 11 | Parque Nacional do Caparaó | -20.446 | -41.834 |
| 12 | Parque Estadual do Ibitipoca | -21.717 | -43.917 |
| 13 | Parque Nacional do Itatiaia | -22.500 | -44.609 |
| 14 | Parque Nacional da Serra dos Órgãos | -22.452 | -42.991 |
| 15 | Reserva Particular do Patrimônio Natural Bacchus | -22.370 | -42.490 |
